# Risk factors for eating disorders: findings from a rapid review

**DOI:** 10.1186/s40337-022-00717-4

**Published:** 2023-01-17

**Authors:** Sarah Barakat, Siân A. McLean, Emma Bryant, Anvi Le, Peta Marks, National Eating Disorder Research Consortium, Phillip Aouad, Sarah Barakat, Robert Boakes, Leah Brennan, Emma Bryant, Susan Byrne, Belinda Caldwell, Shannon Calvert, Bronny Carroll, David Castle, Ian Caterson, Belinda Chelius, Lyn Chiem, Simon Clarke, Janet Conti, Lexi Crouch, Genevieve Dammery, Natasha Dzajkovski, Jasmine Fardouly, Carmen Felicia, John Feneley, Amber-Marie Firriolo, Nasim Foroughi, Mathew Fuller-Tyszkiewicz, Anthea Fursland, Veronica Gonzalez-Arce, Bethanie Gouldthorp, Kelly Griffin, Scott Griffiths, Ashlea Hambleton, Amy Hannigan, Mel Hart, Susan Hart, Phillipa Hay, Ian Hickie, Francis Kay-Lambkin, Ross King, Michael Kohn, Eyza Koreshe, Isabel Krug, Anvi Le, Jake Linardon, Randall Long, Amanda Long, Sloane Madden, Sarah Maguire, Danielle Maloney, Peta Marks, Sian McLean, Thy Meddick, Jane Miskovic-Wheatley, Deborah Mitchison, Richard O’Kearney, Shu Hwa Ong, Roger Paterson, Susan Paxton, Melissa Pehlivan, Genevieve Pepin, Andrea Phillipou, Judith Piccone, Rebecca Pinkus, Bronwyn Raykos, Paul Rhodes, Elizabeth Rieger, Sarah Rodan, Karen Rockett, Janice Russell, Haley Russell, Fiona Salter, Susan Sawyer, Beth Shelton, Urvashnee Singh, Sophie Smith, Evelyn Smith, Karen Spielman, Sarah Squire, Juliette Thomson, Marika Tiggemann, Stephen Touyz, Ranjani Utpala, Lenny Vartanian, Andrew Wallis, Warren Ward, Sarah Wells, Eleanor Wertheim, Simon Wilksch, Michelle Williams, Stephen Touyz, Sarah Maguire

**Affiliations:** 1 InsideOut Institute for Eating Disorders, University of Sydney, Sydney Local Health District, Sydney, Australia; 2 School of Psychology and Public Health, La Trobe University, Melbourne, Australia; 3 Healthcare Management Advisors, Melbourne, Australia; 4 Faculty of Medicine and Health, Charles Perkins Centre (D17), InsideOut Institute, University of Sydney, Level 2, Sydney, NSW 2006 Australia; 5 School of Psychology, Faculty of Science, University of Sydney, Sydney, NSW Australia; 6 School of Psychology and Public Health, La Trobe University, Melbourne, VIC Australia; 7 School of Psychology, Western Australia, Perth, Australia; 8 Eating Disorders Victoria, Melbourne, VIC Australia; 9 Perth, WA Australia; 10 Medicine, Dentistry and Health Sciences, University of Melbourne, Melbourne, VIC Australia; 11 School of Life and Environmental Sciences, University of Sydney, Sydney, NSW Australia; 12 Eating Disorders Queensland, Brisbane, QLD Australia; 13 Sydney Local Health District, New South Wales Health, Sydney, Australia; 14 Westmead Hospital, Sydney, NSW Australia; 15 Translational Health Research Institute, Western Sydney University, Sydney, NSW Australia; 16 Brisbane, QLD Australia; 17 School of Psychology, University of New South Wales, Sydney, NSW Australia; 18 University of Sydney, Sydney, NSW Australia; 19 New South Wales Health, Sydney, NSW Australia; 20 School of Psychology, Faculty of Health, Deakin University, Geelong, VIC Australia; 21 School of Population Health, Faculty of Health Sciences, Curtain University, Perth, Australia; 22 Hollywood Clinic, Ramsay Health Care, Perth, Australia; 23 Melbourne School of Psychological Sciences, University of Melbourne, Victoria, Australia; 24 Queensland Eating Disorder Service, Brisbane, QLD Australia; 25 Hunter New England Local Health District, Sydney, NSW Australia; 26 St Vincent’s Hospital Network Local Health District, Sydney, NSW Australia; 27 Brain and Mind Centre, University of Sydney, Sydney, Australia; 28 School of Medicine and Public Health, University of Newcastle, Sydney, NSW Australia; 29 Westmead Hospital, Sydney, Australia; 30 Healthcare Management Advisors, Victoria, Australia; 31 College of Medicine and Public Health, Flinders University, Adelaide, SA Australia; 32 Exchange Consultancy, Redlynch, NSW Australia; 33 Eating Disorders Service, Children’s Hospital at Westmead, Sydney, NSW Australia; 34 The Bouverie Centre, School of Psychology and Public Health, La Trobe University, Victoria, Australia; 35 Clinical Excellence Queensland, Mental Health Alcohol and Other Drugs Branch, Brisbane, QLD Australia; 36 College of Health and Medicine, Australian National University, Canberra, ACT Australia; 37 ADHD and BED Integrated Clinic, Melbourne, VIC Australia; 38 Department of Psychology and Counselling, La Trobe University, Melbourne, VIC Australia; 39 School of Health and Social Development, Faculty of Health, Deakin University, Geelong, VIC Australia; 40 Swinburne Anorexia Nervosa (SWAN) Research Group, Centre for Mental Health, School of Health Sciences, Swinburne University, Melbourne, VIC Australia; 41 Children’s Health Queensland Hospital and Health Service, Brisbane, QLD Australia; 42 Centre for Clinical Interventions, Western Australia Health, Perth, WA Australia; 43 Central Clinical School Brain and Mind Research Institute, University of Sydney, Sydney, NSW Australia; 44 Ramsay Health Care, Perth, Australia; 45 Department of Paediatrics, The University of Melbourne, Melbourne, Australia; 46 National Eating Disorders Collaboration, Melbourne, VIC Australia; 47 The Hollywood Clinic Hollywood Private Hospital, Ramsey Health, Perth, Australia; 48 Sydney, NSW Australia; 49 The Butterfly Foundation, Sydney, Australia; 50 College of Education, Psychology and Social Work, Flinders University, Adelaide, SA Australia; 51 Eating Disorder Service, The Sydney Children’s Hospital Network, Westmead Campus, Sydney, Australia; 52 Department of Psychiatry, University of Queensland, Brisbane, Australia; 53 University of Tasmania, Hobart, TAS Australia; 54 Royal Hobart, Tasmanian Health Service, Hobart, TAS Australia

**Keywords:** Risk factors, Eating disorders, DSM-5, Aetiology, review

## Abstract

**Background:**

Risk factors represent a range of complex variables associated with the onset, development, and course of eating disorders. Understanding these risk factors is vital for the refinement of aetiological models, which may inform the development of targeted, evidence-based prevention, early intervention, and treatment programs. This Rapid Review aimed to identify and summarise research studies conducted within the last 12 years, focusing on risk factors associated with eating disorders.

**Methods:**

The current review forms part of a series of Rapid Reviews to be published in a special issue in the Journal of Eating Disorders, funded by the Australian Government to inform the development of the National Eating Disorder Research and Translation Strategy 2021–2031. Three databases were searched for studies published between 2009 and 2021, published in English, and comprising high-level evidence studies (meta-analyses, systematic reviews, moderately sized randomised controlled studies, moderately sized controlled-cohort studies, or population studies). Data pertaining to risk factors for eating disorders were synthesised and outlined in the current paper.

**Results:**

A total of 284 studies were included. The findings were divided into nine main categories: (1) genetics, (2) gastrointestinal microbiota and autoimmune reactions, (3) childhood and early adolescent exposures, (4) personality traits and comorbid mental health conditions, (5) gender, (6) socio-economic status, (7) ethnic minority, (8) body image and social influence, and (9) elite sports. A substantial amount of research exists supporting the role of inherited genetic risk in the development of eating disorders, with biological risk factors, such as the role of gut microbiota in dysregulation of appetite, an area of emerging evidence. Abuse, trauma and childhood obesity are strongly linked to eating disorders, however less conclusive evidence exists regarding developmental factors such as role of in-utero exposure to hormones. Comorbidities between eating disorders and mental health disorders, including personality and mood disorders, have been found to increase the severity of eating disorder symptomatology. Higher education attainment, body image-related factors, and use of appearance-focused social media are also associated with increased risk of eating disorder symptoms.

**Conclusion:**

Eating disorders are associated with multiple risk factors. An extensive amount of research has been conducted in the field; however, further studies are required to assess the causal nature of the risk factors identified in the current review. This will assist in understanding the sequelae of eating disorder development and in turn allow for enhancement of existing interventions and ultimately improved outcomes for individuals.

**Supplementary Information:**

The online version contains supplementary material available at 10.1186/s40337-022-00717-4.

## Plain English summary

Research into the risk factors associated with eating disorders (EDs) is necessary in order to better understand the reasons why people develop EDs and to inform programs which aim to reduce these risk factors. In the current study we reviewed studies published between 2009 and 2021 which had researched risk factors associated with EDs. This study is one review of a wider Rapid Review series conducted as part the development of Australia’s National Eating Disorders Research and Translation Strategy 2021–2031. The findings from this review are grouped into nine main risk factor categories. These include (1) genetics, (2) gastrointestinal microbiota and autoimmune reactions, (3) childhood and early adolescent exposures, (4) personality traits and comorbid mental health conditions, (5) gender, (6) socio-economic status, (7) ethnic minority, (8) body image and social influence, and (9) elite sports. Further research is needed to better understand the relationship between the risk factors, in particular the ways in which they may interact with each other and whether they cause the ED or are just associated with the ED.

**Supplementary Information:**

The online version contains supplementary material available at 10.1186/s40337-022-00717-4.

## Introduction

Eating disorders (ED) are complex psychiatric conditions associated with significant psychological and physical impairment. Individuals with EDs are at greater risk of suicide attempts, mortality, and poorer quality of life relative to both the general population and individuals with other psychiatric conditions [1–3]. Central to addressing the pervasive nature of EDs is understanding the circumstances which make individuals more vulnerable to developing these psychiatric conditions. The development of an ED is dependent on a myriad of variables ranging from sociocultural, to biological and genetic, and psychological factors. Despite the variation and complexity present in the aetiology of EDs, efforts have been made by researchers to identify risk factors which commonly predict onset [4–6]. Understanding the range of risk factors and their potential contribution to onset of an ED is crucial to identifying at risk groups and providing effective screening and prevention programs, as well as targeted interventions [7, 8].

EDs can be severe and are often chronic in nature, particularly if not addressed in a timely manner. A recent study of ED patients identified an average delay of 5.28 years between ED symptom onset and treatment-seeking [9]. A factor considered to contribute to this delay is health professionals’ lack of awareness of indicators of disordered eating behaviours, meaning EDs often go unrecognised by treating clinicians [10]. Identification of risk factors for EDs offers an opportunity for targeted education of health professionals to assist in distinguishing patterns of psychosocial, biological, and genetic vulnerabilities for disordered eating even in the absence of any overt weight or dietary concerns [11].

Knowledge of the risk factors for EDs offers the opportunity for early identification of high-risk groups and in turn a timely and tailored response via avenues such as public policy development or initiation of targeted prevention programs [12]. Prevention and early intervention programs based upon aetiological models may help to prevent movement along the spectrum from at-risk to full threshold disorder [13]. Additionally, EDs are complex psychiatric conditions with a somewhat limited range of efficacious evidence-based interventions [14, 15]. In addition, a significant number of patients with EDs do not respond to current evidence-based treatments [16–20]. As such, attempts to better understand the role of risk factors in aetiological and causal pathways of EDs are necessary in order to form more nuanced conceptualisations of these illnesses. This may inform the development of more effective treatments, especially for those with persistent and chronic course [21].

The current Rapid Review paper forms part of a series of reviews commissioned by the Australian Federal Government to inform the Australian National Eating Disorders Research and Translation Strategy 2021–2031[22]. This paper aims to identify and explore the risk factors associated with EDs by summarising the existing evidence related to aetiological underpinnings. Importantly, the review is inclusive of research which considers risk factors to be either causal in nature or associated with the onset of ED.

## Methods

The Australian Government Commonwealth Department of Health funded the InsideOut Institute for Eating Disorders (IOI) to develop the Australian Eating Disorders Research and Translation Strategy 2021–2031 [1] under the Psych Services for Hard to Reach Groups initiative (ID 4-8MSSLE). The strategy was developed in partnership with state and national stakeholders including clinicians, service providers, researchers, and experts by lived experience (including consumers and families/carers). Developed through a two-year national consultation and collaboration process, the strategy provides the roadmap to establishing EDs as a national research priority and is the first disorder-specific strategy to be developed in consultation with the National Mental Health Commission. To inform the strategy, IOI commissioned Healthcare Management Advisors (HMA) to conduct a series of RRs to broadly assess all available peer-reviewed literature on the six DSM-5 listed EDs.

A RR Protocol [23] was utilised to swiftly synthesise evidence in order to guide public policy and decision-making [24]. This approach has been adopted by several leading health organisations including the World Health Organisation [25] and the Canadian Agency for Drugs and Technologies in Health Rapid Response Service [26], to build a strong evidence base in a timely and accelerated manner, without compromising quality. A RR is not designed to be as comprehensive as a systematic review – it is purposive rather than exhaustive and provides actionable evidence to guide health policy [27].

The RR is a narrative synthesis and sought to adhere to the PRISMA guidelines [28]. It is divided by topic area and presented as a series of papers. Three research databases were searched: ScienceDirect, PubMed and Ovid/Medline. To establish a broad understanding of the progress made in the field of EDs, and to capture the largest evidence base from the past 12 years (originally 2009–2019, but expanded to include the preceding two years), the eligibility criteria for included studies into the rapid review were kept broad. Therefore, included studies were published between 2009 and 2021, in English, and conducted within Western healthcare systems or health systems comparable to Australia in terms of structure and resourcing. The initial search and review process was conducted by three reviewers between 5 December 2019 and 16 January 2020. The re-run for the years 2020–2021 was conducted by two reviewers at the end of May 2021.

The RR had a translational research focus with the objective of identifying evidence relevant to developing optimal care pathways. Searches therefore used a Population, Exposure, Outcome (PEO) approach [29] whereby search terms are specified to identify literature relating to the population or group of interest (i.e., individuals of any age or background with the propensity to develop and eating disorder), exposure to the risk factors that are associated with the development of an eating disorder, and the outcome of interest (i.e., the development of an eating disorder). By using the three PEO components to guide the search strategy, the PEO approach aims to facilitate a thorough and systematic examination of existing literature. Purposive sampling focused on high-level evidence studies such as: meta-analyses; systematic reviews; moderately sized randomised controlled studies (RCTs) (*n* > 50); moderately sized controlled-cohort studies (*n* > 50), or population studies (*n* > 500). However, the diagnoses ARFID and UFED necessitated a less stringent eligibility criterion due to a paucity of published articles. As these diagnoses are newly captured in the DSM-5 (released in 2013, within the allocated search timeframe), the evidence base is emerging and fewer studies have been conducted. Thus, smaller studies (n =  < 20) and narrative reviews were also considered and included. Grey literature, such as clinical or practice guidelines, protocol papers (without results) and Masters’ theses or dissertations, was excluded. Other sources (which may not be replicable when applying the current methodology) included the personal libraries of authors, yielding four additional studies (see Additional File 1). This extra step was conducted in line with the PRISMA-S: an extension to the PRISMA Statement for Reporting Literature Searches in Systematic Reviews [30].

Full methodological details including eligibility criteria, search strategy and terms and data analysis are published in a separate protocol paper [31]. The full RR included a total of 1320 studies (see Additional File 1 for PRISMA flow diagram). Data from included studies relating to risk factors for EDs were synthesised and are presented in the current review.

## Results

The Rapid Review identified 284 studies for inclusion in the ‘Risk Factors’ category. When referring to ‘risk factors’ in this review, we are not always referring to *causal* risk factors. Accordingly, some of the risk factors included in this review are correlated or associated with increased risk of an ED, without evidence of causation. As the aim of a Rapid Review is to broadly synthesise findings, we did not narrow to studies only providing evidence regarding the causal relationship of risk factors. Rather, the current review focused on a range of research including prospective, experimental and correlational studies to identify a large number of potential correlates which have risk capacity for EDs. According to the Kraemer et al. (2001) criteria, this review covers research related to the following technical terms: “correlate” (a measure associated with the outcome), “risk factor” (a measure which precedes the outcome), and “causal risk factor” (a risk factor, which when manipulated, causes a change in the outcome) [32]. Therefore, the factors identified in this review are associated or predictive factors, unless in cases where a causative link has been demonstrated. A summary of the key risk factors associated with EDs is provided in Table 1 and are discussed in this section. Results are subdivided into nine categories: (1) genetics, (2) gastrointestinal microbiota and autoimmune reactions, (3) childhood and early adolescent exposures, (4) personality traits and comorbid mental health conditions, (5) gender, (6) socio-economic status, (7) ethnic minority, (8) body image and social influence, and (9) elite sports. A full list of included studies for this topic, including population, aims, design, and outcome measures is available in Additional File 1.

**Table 1:** Risk factors associated with EDs

Risk factor category	Features of risk factor	Associated ED
Genetic	See Sect. Introduction and Table 2 for details	AN, BN, BED
Gut microbial dysbiosys	*Escherichia Coli (*CIpB)	AN
Autoimmune disease	Diabetes, inflammatory gastrointestinal disease	AN, BN, EDNOS
Childhood weight status	Low BMI	AN
	High BMI	BN, BED
Relationship with parents	Parent perception that the child is overweight	AN-BP, BN, BED, PD
	Parental teasing about weight	AN-BP, BN, BED
	Perceived pressure from parents to eat	ARFID
Neglect/abuse/trauma		AN, BN, BED, PD
	Post-traumatic stress disorder	BED
Personality traits	Perfectionism	AN-R, A-AN
	Obsession	AN-R, A-AN
	Impulsiveness	AN-BP, BN, BED, PD
Comorbid conditions	Obsessive compulsive disorder	AN
	Social anxiety disorder	BN, AN
	Borderline personality disorder	BN, BED, PD
	Bipolar disorder	BN, BED
	Depression	All EDs
Social/environmental	Exposure to ‘thin ideal’	All ED
	Body dissatisfaction	BN, BED, PD
	Early puberty development	BN, AN
	Food insecurity	Binge eating behaviours
	High educational attainment	Restricting type ED behaviours
	Involvement in elite sports	All ED

*ED* eating disorder; *AN* anorexia nervosa; *BN* bulimia nervosa; *BED* binge eating disorder; *EDNOS* eating disorder not otherwise specified; *BMI* body mass index; *AN-BP* anorexia nervosa (binge-purge subtype); *PD* personality disorder; *ARFID* avoidant restrictive food intake disorder; *AN-R* anorexia nervosa (restrictive subtype); *A-AN* atypical AN

## 1. Genetics: endocrines and neurotransmitters

Genetic risk factors and polymorphisms (variations in gene expression), relating to core EDs have been widely studied. Research conducted within twins and family groups as well as large-scale genomic studies have indicated a genetic component to risk of Anorexia Nervosa (AN), Bulimia Nervosa (BN) and Binge Eating Disorder (BED) [33]. Incidence rates in individuals with a parent with a history of ED have been found to be over twice as high compared to individuals with parents with no history of an ED [34]. Familial studies have demonstrated a strong genetic association for AN in particular. An individual is 11 times more likely to develop AN if they have a relative with the disorder as compared to someone with no family history. Similarly, an individual is 9.6 times more likely to develop BN, and 2.2 times more likely to develop BED if they have a relative with the disorder [33]. Evidence of genetic risk factors for other EDs is growing [33], although there have been no genetic studies to date conducted with Avoidant Restrictive Food Intake Disorder (ARFID) [35].

### Anorexia nervosa and bulimia nervosa

Genetic factors have been shown to strongly contribute to both AN and BN [36]. There is evidence to suggest approximately half of the genetic factors implicated in AN and BN are shared between the disorders, with the remaining 50% being unique to one or the other [36]. An older study of Norwegian twins found some support for different features of AN being more heritable than others; having found weight/shape concern to have greater genetic association than low BMI and amenorrhea [37]. In contrast the landmark 2019 study by two international genome-wide association consortiums found that both metabolic and anthropometric related genetic loci associated with BMI lowering alleles have strong correlations with AN [38].

#### Gender

Hereditary patterns of EDs have been shown to disproportionately affect females [34]. In a sample of adolescent twins aged 15 to 17, Baker et al. (2009) found females were at greater genetic risk for disordered eating than males [39]. This is consistent with earlier evidence suggesting drive for thinness and body dissatisfaction showed lower heritability in males [40]. Baker et al. [39] found that only half of the genetic risk factors predicting drive for thinness and body dissatisfaction in females predicted the same traits in males. A possible explanation for this difference was offered in a study of French and German cohorts whereby inherited variations in an estrogen receptor gene (ESR1) significantly increased risk of restrictive eating and subsequently development of AN restrictive subtype (AN-R) [41].

#### Comorbidities

Genetic risk has been implicated in co-occurrence of EDs and other psychiatric diagnoses. Genetic associations have been found between Attention-Deficit/Hyperactivity Disorder (ADHD) and all EDs, with the strongest correlation to binge/purge-type ED behaviours [42, 43]. Strong positive genetic associations have also been identified between AN and other psychiatric comorbidities, including Obsessive Compulsive Disorder (OCD), major depressive disorder, suicidality, schizophrenia, neuroticism, autism, and neurodevelopmental delay [44–48]. Genetic risk for comorbid AN and Generalised Anxiety Disorder (GAD) has also been identified [46, 47].

The contribution of comorbid mental health disorders to ED risk and outcomes are further discussed in Sect. Results and in another topic paper of the Rapid Review, ‘Psychiatric Comorbidities and Medical Complications.’

#### Genes and polymorphisms

Several genomic studies have attempted to locate specific gene loci implicated in the development of EDs. See Table 2 for a summary of genes and polymorphisms identified in ED genomic studies. A recent genome-wide association study published in 2021 has suggested that there is a distinct difference in the underlying biology between binge-type EDs (BN and BED) and AN. The study reported that both BN and BED shared genomic variant with overweight and obesity, whereas the directions of these associations were reversed for AN [49].

**Table 2:** Genes and polymorphisms identified in the development of EDs

ED	Gene	Polymorphism (allele)	Function
AN	*CADM1*		Cell adhesion molecule 1
	*MGMT*		O-6-methylguanine-DNA methyltransferase
	*FOXP1*		Cell and tissue-specific gene transcription
	*PTBP2*		Polypyrimidine tract binding protein primarily expressed in the brain
	*HTR1B*	G861	5-hydroxytryptamine receptor 1B
	*AGRP*	rs13338499	Agouti-related protein (appetite stimulator)
	*SLC6A4*	5-HTTLPR (S)	Serotonin transporter
	*OXT-R*	rs2254298	Oxytocin receptor
	*ESR1*	rs3798577	Estrogen receptor
	*EPHX2*	rs2291635	Cholesterol metabolism/BMI
	*CNTF*	rs550942	Ciliary neurotrophic factor receptor
BN	*ESR1*	rs928554	Estrogen receptor
	*CNR1*	Rs1049353	Cannabinoid receptor
	*SLC6A4*	5-HHTLPR	Serotonin receptor
	*DRD2*	Rs1800497(Taq1A)	Dopamine receptor D2
	*DRD4*		
	*COMT*	Rs4680 (Va1158Met)	Catechol-O-methyltransferase
	*FTO*	Rs9939609	BMI and fat mass
	*NR3C1*	Bc11	Glucocorticoid receptor
	*NTRK2*	rs1078947	Tyrosine receptor kinase (obesity and mood disorder related)
	*GR*	rs6198	Glucocorticoid receptor
BED	*GHRL*	Rs696217	Ghrelin
	*MC4R*		Melanocortin 4 receptor
	*DRD2*	rs6277	Dopamine receptor D2
		rs1800497	
	*ANKK1*	Rs1800497 (Taq1A)	Ankyrin repeat and kinase domain containing 1
	*DAT1*	Rs2270912	Dopamine transporter 1
		Rs2863130	
	*SLC6A4*	5-HHTLPR	Serotonin transporter
	*OPRM1*	Rs1799971 (118A/G)	Dopamine receptor D2
	*BNDF*	Rs6265 (Val66Met)	Brain derived neurotropic factor
	*FTO*	rs1558902	BMI and fat mass

*ED* eating disorder; *AN* anorexia nervosa; *BN* bulimia nervosa, *BED* binge eating disorder

Genetic susceptibility to AN was explored in a landmark meta-analysis of 33 datasets from international genome-wide association studies. Watson et al. [38] compared the DNA of almost 17,000 individuals with AN to the DNA of 55,000 people without AN around the world. Eight loci associated with significant risk of developing AN were identified [38, 50], including genetic correlations with certain psychiatric, anthropometric, and metabolic traits, as well as physical activity. Positive associations were found for physical activity, anxiety and schizophrenia disorders, and HDL cholesterol. Negative associations were found for metabolic (including glycemic), lipid, and anthropometric traits including fat mass, fat-free mass, BMI, obesity, type 2 diabetes, fasting insulin, insulin resistance, and leptin [48]. Analysis of causality revealed a bi-directional relationship between potential AN genes and risk for low body mass index (BMI). However, there is stronger evidence that low-BMI-causing alleles increase risk of AN than there is for AN-risk genes leading to low BMI [38].

A study of Norwegian adolescents found an association between poor appetite and undereating, and the COMT gene, which is responsible for regulating dopamine levels through the production of the COMT enzyme [51]. Brain studies of patients with AN have indicated that, due to disturbances in regular serotonin and dopamine reward pathways, individuals with AN may use restricted eating as a mechanism to reduce anxiety [52]. In one study of patients with AN and BN, mutations in genes with heightened expression in brain tissue (CNTF, NTRK) were associated with a higher minimum lifetime BMI and earlier ED onset [53].

Six genetic polymorphisms have been associated with the development of BN in people with obesity [54]. Of the six genetic polymorphisms, three are thought to be related to the neuroendocrine receptors of dopamine, serotonin, and cannabinoid. This association is supported by evidence that genetic variations which lead to low dopamine production and neurotransmission are associated with an increased risk of binge/purge type EDs [55]. The remaining three polymorphisms identified in BN aetiology were associated with an estrogen receptor, the production of an enzyme expressed in brain tissue, and the FTO gene (which has a role in BMI regulation) [54]. While dopamine and serotonin receptor genes (DRD2 and SLC6A4, respectively) are implicated in the development of both BN and BED, differing polymorphisms in these genes appear to be associated with increased risk of developing one disorder over the other [54]. Further, triallelic^1^ variations in a serotonin receptor allele (5-HTTLPR) have also been observed to contribute to compulsive personality traits and the development of AN, BN, and eating disorder not otherwise specified (EDNOS) [56, 57]. A polymorphism of the oxytocin receptor gene (OXT-R) was also found to distinguish between risk of onset for restricting type EDs or binge/purge type EDs, indicating the potential role of oxytocin in the development and maintenance of EDs [58]. Additional research has identified an association between a polymorphism in a neurotransmitter inhibition gene (HTR1B) and an increased risk of developing BN as well as greater severity of AN symptoms, including low BMI [59].

Expression of genes associated with the production of appetite and weight control endocrines (leptin, melanocortin, and neurotrophin) are thought to have a role in ED development and severity [45]. A case–control study by Zeeland et al. [60] found a significant number of AN participants with a polymorphism in a cholesterol metabolism gene (EPHX2), which was also associated with lower BMI (see Table 2). Yilmaz et al. (2014) examined 20 single-nucleotide polymorphisms^2^ (SNPs) in the endocrine system genes in a sample of individuals with BN (n = 745) and AN (n = 245). Although no significant differences were observed between either ED diagnosis or control participants, two SNPs associated with regulation of BMI were found to have an impact on disease severity (See Table 2) [61].

Consequences of variations in endocrine signalling in individuals with ED also include reduced capacity for interoception^3^ particularly relating to gastric interoception. A systematic review of interoception in individuals with ED found the strongest correlations were observed in individuals with AN who consistently had lower gastric interoception relating to satiety and self-reported fullness, while individuals with BN were found to have lower pain interoception resulting in higher pain thresholds. However, researchers were unable to ascertain whether lack of gastric interoception in individuals with AN was a result of conscious processing of satiety cues or disruptions in endocrine signalling [62].

#### Non-shared vs. shared environments

A Swedish study of female monozygotic (identical) and dizygotic (fraternal) twins aged between 20 and 47 found that nonshared environmental factors between twins had a greater impact on ED risk than shared environmental factors [36]. This finding was further supported by a study of an Australian twin sample, which concluded that nonshared environmental factors contributed to the genetic factors associated with weight loss behaviours and overeating behaviours in AN and BN, respectively [63]. Shared environmental factors were not observed to have an impact on disordered eating behaviours [63].

Exposure to childhood trauma has been linked to polymorphisms in genes expressed in the glucocorticoid receptor pathway which are associated with increased risk of developing BN, binge eating, and loss of control over eating [51, 64–66]. This finding is supported by research conducted by Monteleone et al. [67], who found significantly lower levels of cortisol in individuals with AN and BN with a history of childhood maltreatment than healthy controls and those ED patients with no history of childhood trauma. Exposure to childhood trauma was also found to interact with gene expression through creating higher levels of DNA methylation^4^ in women with BN [68]. Analysis of evidence from seven studies found a strong additive effect for serotonin transporter 5-HTTLPR polymorphism combined with childhood experiences of physical and sexual abuse in the development of BN [69]. Childhood trauma and abuse as a risk factor for EDs, particularly related to environmental influence, will be further discussed in Sect. Results.

### Binge eating disorder

Variation in genes linked to appetite and satiety modulating hormones such as ghrelin are often implicated in the development of BED, as well as several genes related to regulation of BMI and fat storage. A study of 4,360 adolescents aged 14 or 16 found that frequency of binge eating was associated with expression of a polymorphism in the FTO gene, thought to play a role in BMI and obesity [70]. Further, mutations of the MC4R gene, involved in metabolism and feeding, is also associated with BED and obesity [71, 72].

As previously discussed, polymorphisms in genes responsible for the production of neuroendocrine receptors such as dopamine and serotonin are also commonly associated with BN and BED [54]. Reward responses to food have long been implicated in the development and perpetuation of BED. The expression of two alleles in the dopamine D2 receptor has been found to be positively associated with BED in a sample of 230 individuals with obesity [73]. The authors concluded that expressions of these alleles was associated with hypersensitivity to reward, likely having a causal relationship with BED [73]. In a study of female twins in the US, increased binge eating frequency was also found to be associated with genetic factors related to the personality traits neuroticism and conscientiousness [74].

### Night eating syndrome

Genetic research relating to Night Eating Syndrome (NES) is less developed than the primary EDs. Work in animal models has implicated variants of the VGF, a gene responsible for production of a neuropeptide precursor in NES aetiology [75, 76]. One familial study was identified assessing the heritability of NES involving families where at least one parent had obesity. Night eating symptoms in mothers were strongly associated with similar behaviours in their sons and daughters, while no such correlation was observed for fathers [77]. Interestingly, the association was slightly stronger in sons (r = 0.19) than in daughters (r = 0.15), whereas heritability relationships are typically stronger in female offspring in other ED diagnoses [34, 77]. This finding was further supported by evidence from a Swedish twin registry study where males were more likely to endorse night eating traits associated with genetic factors, while females were more likely to endorse binge eating [76]. Further research is required to understand any potential genetic risk factors associated with NES.

### Summary

There is considerable evidence pointing to genetic risk in the development of EDs, with the highest heritability conferred for AN [33, 34]. Females are also at greater genetic risk for disordered eating in comparison to males [39]. When considering the specific genetic variations thought to contribute to increased ED risk, genetic associations have been found between EDs and other psychiatric comorbidities, however the type of comorbidity differs according to the ED diagnosis. For binge-type EDs (BN and BED) strongest genetic correlations are observed with ADHD [42, 43] whilst AN has strong correlations with OCD, MDD, suicidality, schizophrenia, neuroticism, autism, and neurodevelopmental delay [44–48]. In a similar manner, genetic correlations with metabolic traits appear to differ between ED diagnoses, such that BN and BED have been found to share genomic variants with overweight and obesity [49] whereas potential AN genes uphold a bi-directional relationship with low BMI [38]. Genes associated with other metabolic functions, including appetite and weight control endocrines (leptin, melanocortin, neurotrophin) have also been implicated in ED development and severity, however fewer differences between ED diagnoses are apparent. Polymorphisms in the genetic loci responsible for neurotransmitters associated with reward processing and appetite regulation hormones, including dopamine, serotonin, and cannabinoid have been identified as a risk factor across several ED diagnoses including AN, BN, and EDNOS [45, 50–62]. Additionally, genetic polymorphisms in the glucocorticoid receptor pathway responsible for the stress response have been linked to individuals who have experienced trauma and are associated with increased risk for BN [51, 65, 66].

## 2. Gastrointestinal microbiota and autoimmune reactions

### Gastrointestinal microbiota

The role of gut microbiota and immune system reactions in the development and perpetuation of EDs is an emerging field, however is receiving growing attention. Endocrines produced in the gastrointestinal (GI) tract communicate with the brain to regulate functions of appetite and satiety. Given the role of these functions in EDs, it is thought that dysregulation of the gut microbiome may be partially responsible for ED psychopathology [78–80]. A review of evidence on the gut microbiome suggests that the growth cycle of gut bacteria and their metabolites^5^ may contribute to patterns of accelerated and/or prolonged satiety in AN and periodic lack of satiation in BN [78]. In a study of 33 AN patients undergoing refeeding, Hanachi et al. [81] found the AN patients to have significant gut microbial dysbiosis compared with 22 healthy controls.

Several studies of AN have investigated the role of a protein (CIpB) produced by the *Escherichia Coli* (*E. Coli*) bacteria. The CIpB protein has a similar structure to the human hormone responsible for simulating secretion of satiation peptide YY. The peptide YY has been detected in high levels in the blood plasma of individuals with AN compared to healthy controls [78, 82, 83]. Peptide YY levels have also been found to be elevated among individuals with AN-R as compared to those with AN-BP and healthy controls [84]. Intestinal infections and chronic inflammation can lead to large increases in the number of *E. coli* bacteria in the GI tract, therefore increasing the levels of peptide YY and potentially increasing risk of ED [83]. The CIpB protein produced by *E. Coli* also prompts an immune reaction whereby autoantibodies are created. The position on the receptor for this autoantibody has been shown to differentiate between risk for BN and BED or AN [78]. Despite such emerging evidence indicating a role for gut microbiome dysregulation in EDs, researchers consider much of the evidence to be in an observational phase or using murine models^6^ and lacking the capacity to explain aspects of ED pathology [79, 85].

### Autoimmune and autoinflammatory diseases

Gut microbiota are also known to interact with autoimmune responses, which have been investigated as a potential risk factor for EDs. In a large population-based cohort study, autoimmune and autoinflammatory diseases were identified as a significant predictor in the development of EDs and were associated with a 36% increased chance of developing AN. Interestingly, risk of BN and EDNOS was much higher at 73% and 72%, respectively [86]. Among a sample of patients hospitalised for EDs in Finland, higher prevalence of type 1 diabetes and Crohn’s disease was observed compared with healthy controls [87]. A recent meta-analysis has also identified a bidirectional association between coeliac disease and EDs. In particular, patients with AN are at a significantly greater risk of coeliac disease than healthy adults without AN [88]. Further, researchers argue that symptoms of ED commonly mimic those of chronic inflammatory GI and endocrine disease, including inflammatory bowel disease and diabetes type 1 and 2, emphasising the importance of screening for possible co-occurrence [89]. Unlike the vast majority of other risk factors associated with EDs, autoimmune and autoinflammatory diseases represented a greater risk for male participants as compared to females [86].

### Diabetes

As a type of autoimmune disease, diabetes is commonly associated with EDs. There is a substantial evidence base indicating an increased prevalence of disordered eating behaviours among individuals with both type 1 and type 2 diabetes [90, 91]. However, much of the evidence is observational and there are limitations in distinguishing between avoidance of certain food groups due to presence of an ED versus a feature of diabetes management [92, 93]. Nevertheless, high rates of ED behaviours not related to food restriction (e.g., excessive exercise, vomiting, and laxative abuse) have been observed in adolescents and adults with diabetes [94, 95]. Insulin manipulation or restriction has also been observed in adolescents with diabetes resulting in poor glycaemic control and poorer outcomes [89, 90, 94–96]. Interestingly, a study of adults has revealed that weight/shape overvaluation was lower in participants with diabetes (31.5%) compared to those who did not have diabetes (41.2%). The authors suggest that this may indicate that BED, as an ED for which weight/shape overvaluation is not a diagnostic criteria, may be of particular concern among adults with diabetes [97].

### Summary

In terms of biological risk factors, evidence has largely focused upon proteins produced by gut bacteria, which have been implicated in dysregulation of appetite and satiety in individuals with EDs. The metabolites of gut bacteria are thought to play a role in disordered eating patterns, including prolonged satiety in AN and periodic absence of satiety in BN [78–80]. For example, a protein produced by *E. Coli* bacteria has been found to mimic the structure of the satiation peptide YY, a protein that is higher in individuals with AN as compared to healthy controls [83, 84]. Findings such as these have led researchers to consider intestinal infections and chronic inflammation as a potential risk factor for EDs. However, research in this field is emerging, with further studies needed to better understand the association between gut microbiome dysregulation and EDs. Large studies have indicated that having an autoimmune or autoinflammatory disease, such as Crohn’s disease, inflammatory bowel disease, diabetes type 1 and 2, and coeliac disease, is also significantly associated with increased risk of BN and EDNOS, and to a lesser extent, AN [90–95].

## 3. Childhood and early adolescent experiences

A range of childhood experiences have been linked to the development of EDs later in life, including in-utero exposures, family dynamics and parental characteristics, childhood weight, and experiences of abuse and trauma.

### In utero exposures

There is evidence to suggest that exposure to certain levels of hormones during foetal development could increase risk of ED development later in life. In a large cohort study of women in the UK, daughters whose mothers had a lifetime diagnosis of BN were found to have been exposed to high levels of prenatal testosterone in the womb, which was implicated in an increased risk of BN and binge eating [98]. However, a large multinational twin study was unable to find any link to in utero exposure to sex hormones and ED onset later in life [99].

Research has indicated that in-utero exposure to high levels of cortisol through maternal stress is associated with later development of ED [100, 101]. A further study in the UK found that individuals who were born preterm had an increased risk of ED associated with structural brain alterations linked to underdevelopment [102]. Additional risk factors include the use of substances during pregnancy (e.g., nicotine) and maternal illness leading to malnutrition (e.g., anaemia), which have also been linked to an increased risk of AN and BN in the child later in life [103].

Risk factors conferred during foetal development are further supported by findings that risk of BED is associated with high weight at birth or being large for gestational age, while AN was associated with low weight at birth. No significant foetal developmental risk factors have been identified for BN [104]. Moreover, stressful events experienced by mothers in the year prior or during pregnancy, in particular the death of a close relative in the six months preceding pregnancy, have been shown to have an impact on the development of feeding or EDs in infants and toddlers [105]. Feeding issues in babies of mothers who had an ED diagnosis during pregnancy were also noted in this cohort [106].

A recent systematic review identified an association between AN and older maternal age, preterm birth (< 32 weeks), lower birth size, and maternal health complications (e.g., preeclampsia, eclampsia). The review also reported an association between BN and maternal stress during pregnancy [107].

There appears to be an impact of pregnancy upon the eating behaviours of women with an ED diagnosis. One study has found that ED behaviours across diagnoses tended to improve significantly during the pregnancy period, although this may not be maintained after [108]. It has also been reported that pregnancy is associated with remission of BN but an increased risk of BED onset [109, 110]. Women with a history of psychosocial adversities have been found to possess a significantly greater risk for BN during pregnancy [111].

### Family dynamics and parental characteristics

Research has shown that children are more likely to develop an ED if their parents display characteristics commonly associated with ED psychopathology, such as drive for thinness and perfectionism [112]. Specifically, maternal history of an ED has been shown to be associated with higher rates of emotional eating in children as young as four years old [113]. The children of women with lifetime AN have also been found to exhibit deficits in cognitive functioning, including social understanding, visual-motor function, planning, and abstract reasoning [114].

Additionally, Larsen et al. [115] reported that general parental psychiatric illness is associated with increased risk of BN and EDNOS. The authors also identified the experience of childhood adversity and significant family disruption as significant risk factors for development of BN and EDNOS. Interestingly, no associations between childhood adversities and risk of AN could be identified by authors, although a separate study identified maternal depressive symptoms as a predictor of AN [116].

Adopted individuals have also been identified as having a greater risk of binge eating and extreme weight loss behaviours, as well as increased risk of a lifetime diagnosis of an ED [117]. Other parental characteristics which have been associated with ED behaviours include high maternal BMI at 16 weeks’ gestation and when their child is eight years old, high maternal education attainment, and low parental self-esteem [118–120].

Individuals’ perceptions of the quality and nature of their parental relationship has been investigated as a potential risk factor for development of an ED. Research has found that female individuals diagnosed with AN or BN report significantly lower perceived emotional connectedness prior to disorder onset than their healthy sisters. In a family-based study of 332 female individuals, low emotional connectedness conferred a greater risk of developing BN over AN-R [121]. Further, females who report low maternal warmth have a higher risk of developing binge/purge type EDs [122]. Low parental warmth appears to be a risk factor for ED development in females but not males [123]. A study of AN patients and their healthy siblings found that both siblings in these families perceived low maternal care and high maternal overprotection. Siblings affected by AN developed insecure attachment compared with their siblings and had higher preoccupation with relationships, while healthy siblings were able to develop secure attachment and low need for approval and high self-transcendence [124]. Other risk factors include an oppressive parental relationship and childhood unhappiness [122].

Parents’ communication about food, as well as parental eating behaviours, have been shown to be a significant risk factor for EDs in their children. Several studies have found that exposure to disordered eating behaviours such as dietary restriction in parents is likely to have an impact on the early development on EDs in children, beyond the influence of genetics [125, 126]. One study identified maternal distress as a mediating factor in the relationship between maternal ED and infant feeding difficulties [127]. Maternal dieting and poor communication among family members have also been associated with long-term risk for restrictive disordered eating [128]. Conversely, parental conversations regarding healthy eating, rather than dieting or weight, and regular family meals were found to be protective against development of EDs among child and adolescent samples in Europe and the US [129, 130]. Parental pressure to eat, early negative experiences with food, and high disgust sensitivity were found to predict picky eating behaviours associated with ARFID. Parental encouragement around food in childhood was observed as a protective factor. Being male was also found to be a significant risk factor for adult picky eating behaviour and potential ARFID [131].

The experience of stressful life events, including bereavement, separation from family members, or involvement in an accident have been found to have an impact on ED development, in particular BN and BED. The occurrence of three or more events in combination with external criticism of weight or shape has been shown to be significant predictors in the year prior to BN onset [132]. No significant differences were observed between BN and BED in terms of the number or types of events experienced prior to onset [133].

### Childhood weight

Research on the association between childhood weight and risk of eating pathology in later years is ambiguous. Several studies have reported that higher weight during childhood poses an increased risk of developing an ED in later years, including among culturally and linguistically diverse (CALD) individuals, as well as males [134–138]. Analysis of specific ED behaviours among adolescents in the US between 1999 and 2010 found that ED symptomatology and weight/shape concern persisted beyond adolescence for individuals who were overweight. Contrastingly, for non-overweight individuals, unhealthy weight control behaviours and body dissatisfaction decreased over time [139]. Other studies have found that adolescents with a weight history in the overweight range experience a significantly greater drop in BMI, higher levels of ED psychopathology and comorbid mental health difficulties, and take much longer to be identified than adolescents without a history of overweight [140], 141.

Contrastingly, explorations of the association between weight history and AN specifically have found that low baseline BMI is a significant risk factor for development of both atypical AN and AN [38, 142, 143].

It has been suggested that parental perception of their child as being overweight may be a more powerful predictor of ED development than the child’s weight itself [118, 144, 145]. The significant impact of parental behaviours on ED risk has been supported by a study comparing individuals with BN to healthy controls and individuals with other psychiatric conditions. While being overweight or obese in childhood was identified as a risk factor, high maternal expectations and negative parental attitudes about weight and obesity in childhood were more strongly associated with the onset of BN among participants [146, 147]. These risk factors are also associated with onset of BED [148]. Negative parental attitude towards childhood weight, including parental teasing about weight, has been shown to have a strong positive association with ED behaviours in both males and females, in particular binge eating behaviours [146, 149, 150]. Parental comments about their child’s weight and eating behaviours are also significantly associated with increased drive for thinness and body dissatisfaction [151, 152].

### Abuse and trauma

Experience of childhood trauma and abuse has been consistently identified as a non-specific risk factor for the development of EDs, although these experiences are more strongly associated with binge-purge type disorders such as BN, BED, and AN-BP [153–157]. Evidence from several studies suggests that emotional abuse is a significant predictor of binge/purge symptomology in women, while sexual abuse and physical neglect were associated with symptoms in men [158–160]. Sexual harassment has also been identified as a risk factor for EDs however little is known about the causal relationship or the role of mediating factors [161]. Attempts to investigate the association between types of childhood trauma and specific ED diagnoses have found that emotional abuse is a risk factor for all core ED symptoms [162]. A large-scale study of young adults in the US found that participants who reported multiple types of maltreatment in childhood were almost twice as likely to report binge eating and skipping meals as compared to those who reported no or low maltreatment [163]. Verbally abusive fathers have been shown to be strongly associated with AN-BP and BN, and verbally abusive mothers influence the development of BN [164].

Studies conducted in groups of women with obesity have found relationships between binge eating and childhood abuse and neglect. The severity of the abuse, rather than the type of abuse, appears to have a role in the development of BED and severity of food addiction [165, 166]. A recent study has found that childhood food neglect is associated with increased risk for BN and BED even after adjusting for other adverse experiences and financial difficulties experienced during childhood [167]. A study on the impact of childhood emotional abuse and ED risk found that low self-perception and self-esteem caused by the abuse contributed to an increased risk of BED and NES [168]. Further, individuals with both an ED diagnosis and a history of childhood trauma and abuse have been found to have increased risk of lifetime suicide attempts [169, 170].

The experience of childhood bullying has been found to increase risk of AN, and to a lesser extent BN, in children and adolescents [171–173]. However, increased risk of EDs was not found to carry on into early adulthood [171]. Weight-based teasing has also been associated with emotional eating, eating in the absence of hunger, and disordered eating attitudes and behaviours [174]. Consistent with existing evidence, an observational study of 182 adolescents receiving treatment for EDs found bullying was the most common form of trauma experienced by patients [175]. Assessment of the impact of cyberbullying also found the experience predicted onset of AN, BN, and EDNOS in a group of individuals with an ED diagnosis and increased ED symptomology and depression among a group of high-risk individuals [176]. Exposure to online content and risk of ED development is discussed further in Sect. Gender.

### Summary

An overview of the evidence regarding the impact of early experiences in terms of ED risk has identified a range of factors starting from the in-utero environment through to adolescence. In-utero exposure to high levels of testosterone, cortisol, or substances have been associated with increased risk of EDs [98–100, 102, 103]. There is also evidence to linking high birth weight to BED and low birth weight to AN [104]. Weight persists as a risk factor throughout childhood and adolescence, with research findings that high maternal expectations and negative parental attitudes about weight are also associated with ED risk. The quality and nature of one’s parental relationship is considered another risk factor for EDs, such that lower ratings of parental warmth or emotional connectedness have been reported by individuals with AN and BN as compared to their healthy siblings [121–124]. Experiences of childhood adversity, significant family disruption, childhood trauma (including neglect and emotional or sexual abuse) are well-documented risk factors, with evidence suggesting that they are most likely to contribute to the development of binge/purge type disorders (AN-BP, BN, BED, PD) [115, 153–156]. Researchers have also suggested that the link between EDs and trauma is likely to be underestimated due to non-disclosure [207].

## 4. Personality traits and comorbid mental health conditions

Traits such as anxiety, perfectionism and obsessive-compulsivity are frequently associated with increased risk of EDs and may play a substantial role in the severity of symptoms, response to treatment, and risk of relapse [178].

### Perfectionism, impulsivity, compulsiveness, and avoidance motivation

Rather than being linked to diagnostic type, a meta-analysis of personality traits (Farstad et al., 2016) found a more robust association with specific behaviours and symptomatology. Studies have shown that relative to controls, individuals with ED have elevated levels of perfectionism (setting of excessively high standards for performance, accompanied by overly critical self-evaluation); neuroticism (tendency to experience negative effects such as anger, anxiety, self-consciousness, irritability, emotional instability, and depression); impulsivity, particularly negative urgency (tendency to engage in impulsive behaviour when experiencing strong negative emotion); compulsivity (tendency toward overcontrolled behaviour); avoidance motivation (tendency to move away from or avoid situations associated with punishment); sensitivity to social rewards; introversion; and self-directedness (goal-oriented behaviour) [178–186].

Perfectionistic traits are common in both AN and BN. A systematic review and meta-analysis concluded that individuals with AN tended to place greater emphasis on high personal standards, while individuals with BN were more likely to perceive high levels of parental criticism [178]. The contribution of perfectionism to ED symptomatology (including dietary restriction and shape and weight overvaluation) was further supported by Joyce et al. [180] in a community-based sample of women. The study was inconclusive as to whether perfectionism was the cause of the ED symptoms. However, a significant positive association between perfectionism and weight and shape overvaluation was observed [180].

Among a sample of adolescent females recruited from an ED service in Australia, researchers found both a direct relationship between perfectionism and AN symptoms as well as an indirect relationship when mediated by depression [187]. The two different relationships were found to be equally viable, further supporting the notion of a reciprocity of symptoms between anxiety, depression, and AN, which are preceded by perfectionism.

In a 10-year follow-up study of university-aged adults in the US perfectionism was associated with the onset of AN, BN, and EDNOS and found to contribute significantly to disorder maintenance [188]. The tendency toward perfectionism in AN has been linked to a trait of vulnerable narcissism, ‘hiding the self,’ described as an unwillingness to show one’s faults or needs to others. The ability to exhibit control over emotional needs and relationships was correlated with AN-R in a comparison study involving individuals with AN and BN. However, the cross-sectional design was unable to determine whether this trait preceded AN-R and the sample size was relatively small [189].

Obsessiveness has also been found to be strongly associated with AN. Among a clinical sample of patients with AN and atypical AN, obsessiveness was positively correlated with a drive for thinness, a key aspect of AN symptomatology. The study did not find any significant differences between AN and atypical AN in terms of obsessive behaviours [190].

Studies seeking to assess personality traits contributing to differences in clinical presentation between restricting and binge/purge ED subtypes conclude that alexithymia – the inability to identify or verbally describe feelings or emotions – plays a role in the emotional dysregulation displayed by both AN-R and BN patients [191, 192]. Higher levels of alexithymia have been associated with greater risk of re-hospitalisation in a three-year follow-up study of women with both AN and BN [193]. Prefit et al.’s [194] meta-analysis of studies into EDs and associated personality traits found lack of emotional awareness and inability to regulate emotions leading to maladaptive ED symptomology was not diagnosis specific [194]. Findings from the meta-analysis support Brown et al. (2018), suggesting a need for emotion-focused treatment approaches such as dialectical behaviour therapy (DBT) [192, 195].

While binge/purge presentations are consistently associated with impulsivity and greater emotional dysregulation [196, 197], one study demonstrated no significant differences in ability to regulate emotions between AN-R and BN patients with high levels of alexithymia [192]. However, in another study involving clinical samples of AN-R, AN-BP and BN patients, individuals with AN-R were found to have fewer fluctuations in mood than individuals with AN-BP and BN. Only in groups exhibiting binge/purge symptomology were these behaviours observed as a method for alleviating negative affect [198]. Similarly, among a group of 139 female college students, lower impulsivity in addition to lower self-esteem was found to be associated with AN risk [199]. A recent systemic review has warned that due to methodological limitations in the studies conducted to date, there is insufficient evidence to support the characterisation of AN and BN as being low and high in impulsivity, respectively [200].

Individuals with binge/purge subtypes EDs, including AN-BP, BN, BED and various OSFEDs, have been found to have higher levels of avoidance motivation, impulsivity, emotional dysregulation, anxiety, depression, and paranoia than healthy controls [178]. Within a clinical sample of AN patients, individuals displaying binge/purge symptoms were more likely to engage in non-suicidal self-injurious behaviour and have lower self-directedness and co-operation than individuals with AN only [201]. However, the literature is inconclusive as to whether these traits contribute to ED onset or are symptoms of it.

Several studies have observed high levels of impulsivity in individuals with BN, with these individuals commonly displaying negative urgency, lack of planning and sensation seeking. Farstadt et al. (2016) in their meta-analysis also argue a role for compulsiveness (i.e., the tendency towards overcontrolled behaviour), suggesting that the interaction of personality traits such as impulsiveness and compulsiveness can have implications for ED symptomology and disorder severity [161, 180, 183, 184]. In this manner, impulsivity was found to have a significant impact on the types of ED symptomatology displayed by the individual and clinical presentation [178, 195]. In contrast, Waxman [195] found no significant differences in impulsivity between ED diagnoses. Waxman [195] suggested that while there is a lack of evidence from longitudinal studies to determine conclusively that impulsivity is a risk factor in the development of ED, evidence from studies using proxy measures such as delinquency found these behaviours preceded BN onset. One further study has reported an association between NES and impulse control disorder [202]. It has also been suggested that impulsivity and addiction-like mechanisms may explain the association between ED psychopathology and both high-risk sexual behaviours and substance misuse [203, 204].

A study of 83 sister pairs found participants with a lifetime ED diagnosis displayed higher levels of internalising behavioural issues (social withdrawal, anxiety, depression) and/or externalising behavioural problems (aggression and delinquency) than their healthy sisters [205]. Internalising behaviours were found to be a strong predictor for AN-R, while externalising behaviours were strongly associated with later onset of bulimic symptoms and BN [205].

Two models illustrating risk of bulimic behaviours among young females have attempted to account for both the role of personality traits and traditional ED concepts of the ‘thin ideal’ [206]. Pearson’s integrated model of risk combines the ‘state-based’ pathway, which shows binge eating as an impulsive lack of control behaviour and purging as a compulsive correction, and the ‘trait-based’ pathway, which emphasises negative urgency as a consistent tendency toward impulsivity and stress alleviation through binge eating. The ‘trait-based’ pathway also considers the role of inherited ED risk and predisposing childhood exposures [206]. Pearson et al. argue that integration of the ‘trait-based’ model considers the important role of heritability and negative urgency that is absent from the Stice model [207]. Further investigation of disease models of bulimic behaviour by Dakanalis et al. [208] indicate that risk factors are more complex than can be mapped by the dual pathway model, citing bi-directional relationship between dietary restriction and negative affect.

Negative urgency has also been found to be an independent predictor of food addiction among individuals displaying binge-eating symptomology [209]. A further study by Utschig et al. [210] indicated that fear of negative evaluation from others is a predictor for body dissatisfaction and pressure to be thin, contributing to an internalised ‘thin ideal’ in individuals with BN and feeding into the state-based model. Fear of negative evaluation is considered an aspect of social anxiety and relates to heightened sensitivity to social rewards, a trait found to be elevated across ED diagnoses [178, 210].

### Personality disorders

The central role of certain personality traits in the perpetuation and potential development of ED symptomology reflects established relationships between some personality disorders and EDs [211–213]. Comorbidity studies have found borderline personality disorder (BPD) to most commonly occur with BN and other binge/purge ED subtypes [212]. This finding is supported by research on personality traits in EDs where avoidant behaviours and low emotion regulation flexibility are elevated in bulimic-type disorders and also a core feature of BPD [178, 212, 214]. However, some researchers argue that the co-occurrence of EDs and personality disorders may have been inflated in previous studies [215]. In a sample of 132 females with ED, prevalence of any personality disorder was 21%, lower than in other studies where reported figures were between 27 and 95% [215]. However, findings from von Lojewski et al. [215] were consistent with existing evidence that BPD traits were significantly associated with binge/purge EDs compared with AN-R. Individuals with comorbid BPD and ED were also more likely to report self-induced vomiting as compared to any other personality disorder. Co-occurrence of EDs and BPD has also been associated with increased risk of engaging in non-suicidal, self-injurious behaviours within a clinical sample [212]. Meta-analysis of 20 studies published between 1987 and 2010 found comorbidity of BPD with EDNOS (now OSFED) to be 38%, and 29% with BED. Researchers indicated that ED and personality disorder comorbidity are more common among individuals with AN and BN than BED and EDNOS [216]. However, among patients with BED or EDNOS, avoidant personality disorders were found to be the most common, followed by BPD [216]. It should however be noted that two of three studies identified by the Rapid Review concerning ED and personality disorders were restricted to relatively small clinical samples without control groups. They were also limited by their cross-sectional design in their capacity to investigate the temporal relationships between disorders.

### Anxiety, mood disorders and psychiatric comorbidities

Co-occurring and preceding mental health conditions, particularly those with shared genetic and experiential influences such as anxiety and mood disorders, are also risk factors for EDs. While it is difficult to assess which condition precedes the other without use of prospective study designs [217] these relationships have been widely studied in AN and BN, and there is some evidence for anxiety and mood disorders including depression and bipolar disorder preceding ED symptomatology. Evidence from a three-year prospective study of 615 pairs of twins in the US suggests elevated risk for AN is associated with higher levels of depression and anxiety in combination with a high drive for thinness, rather than either risk factor alone [218]. There is less conclusive evidence on the relationship between BN, anxiety, and depression although some preliminary research was identified indicating several key symptoms were shared between the three disorders [219].

#### Mood disorders

In clinical ED populations, prevalence of mood disorders is frequently high [220]. In one study, major depressive disorder (MDD) was found to affect 64% of individuals with AN-R and over 75% of binge/purge ED subtypes (AN-BP, BN). Sequencing of disorder onset found that mood disorders preceded ED onset in a third of the AN-R cases and 40% of the AN-BP/BN cases. The remaining comorbid cases were either co-occurring or onset following ED diagnosis. These findings from Godart et al. [220] indicate that depressive disorders can be both a predictor and consequence of ED, as well as a comorbidity caused by malnutrition further complicating management and treatment of EDs.

Assessment of the temporal relationship between depression and disordered eating in an eight-year longitudinal study found depressive symptoms predicted increases in BN behaviours, which in turn predicted increases in depressive symptoms [221]. These findings indicate there may be a reciprocal relationship between the two conditions. A reciprocal relationship was also identified in a larger cohort of adolescent females where individuals who reported depressive symptoms were twice as likely to engage in overeating and binge eating at four-year follow-up, and individuals reporting overeating and binge eating were also more likely to report depressive symptoms at follow-up [222].

#### Anxiety disorders

There is evidence to suggest that anxiety is the most commonly occurring comorbidity with ED [223]. Childhood anxiety disorders have repeatedly been found to precede the onset of an ED, particularly AN [224–228]. Studies have identified a greater incidence of childhood obsessive–compulsive traits in individuals diagnosed with AN in comparison to control groups without an ED [177]. Micali et al. [211] conducted a longitudinal study of 231 young people diagnosed with OCD over a nine year period. Of the 126 participants who completed the follow up assessment, 12.7% had a diagnosis of an ED. Such findings highlight predictive value of childhood anxiety disorders in the later development of EDs, especially AN.

A reciprocal relationship between GAD and AN was indicated in a large twin study by Thornton et al. [229] whereby having GAD significantly increased likelihood of AN and having AN significantly increased likelihood of GAD. The group with AN and GAD had the lower mean adult BMI than both AN only and GAD only groups and healthy controls. These findings indicate the presence of comorbid mental health conditions may exacrerbate EDs and increases severity of symptoms. Sihvola et al. (2009) found co-occurrence of MDD and GAD at age 14 was strongly associated with onset of ED at follow-up (age 17). Weaker associations were observed for both MDD and GAD alone [230].

Ciarma and Mathew [231] investigated the relationship between social anxiety disorder (SAD) and disordered eating among adults aged between 18 and 35 living in the community. This study found self-esteem and stress reactivity resulting from interpersonal conflict to be partial mediators, indicating that ED symptoms can be elicited by heightened responses to stress from social conflict and negative self-view. However, the partial mediation effect observed indicated that other unidentified factors may also have a role in the relationship. A further study of adolescents found evidence of a bidirectional relationship whereby depression and anxiety were risk factors for disordered eating behaviours, which in turn led to increased depression and anxiety [232].

Prevalence of social anxiety was also found to be high among a separate clinical sample of Australian adults with an ED, where 42% were found to have social phobia. It was also the most commonly diagnosed anxiety disorder within each of the ED subtypes, including 33% of those diagnosed with BN, 26% for AN and 25% for EDNOS. Investigations into the temporal relationship between ED diagnosis and anxiety disorder have found many individuals have anxiety prior to their ED diagnosis [225–227]. However, in one systematic review, this was supported only by the included retrospective case–control and cohort studies, and was not supported by evidence from prospective studies included in the review [227]. This discrepancy highlights the potential role of recall bias that may be present across studies relating to anxiety and EDs [227]. OCD and SAD also tend to precede onset of ED, and BN in adolescence may increase risk of SAD and panic disorders in adulthood [233].

In some individuals, shame has been found to predict later onset of BN and social anxiety, indicating a shared risk factor for both conditions [234]. Impaired psychosocial functioning and capacity to maintain interpersonal relationships associated with shame or shyness was also found to predict ED onset among adolescents in the US [235].

#### Psychiatric comorbidities of ED diagnoses other than AN/BN

Evidence relating to mental health comorbidities for EDs other than AN and BN is less developed. Studies conducted investigating BED and NES are confined to clinical samples with cross-sectional designs, highlighting a need for further work in this area, especially considering the high prevalence of psychiatric comorbidities detected in individuals with these diagnoses. Among patients receiving treatment for BED, 74% had a lifetime psychiatric disorder diagnosis, and 43% had a current diagnosis [236]. In a population of overweight and obese patients with severe mental illness, 25% were diagnosed with NES and 6% with BED [237]. Other studies measuring NES in patient samples with depression and bipolar disorder (BD) found the prevalence to be 32.5% and 8.8% respectively [238, 239]. Higher prevalence of NES was detected in both depression and BD groups compared with healthy controls, indicating increased risk among these individuals.

ED and BD comorbidities are also commonly reported in research, with association between BD and BN/BED considered particularly significant, although the casual and temporal relationships between the disorders are not well understood [240–242]. While it is likely that some risk factors are shared, lack of data regarding disorder onset limits commentary on the relative risk BD confers to the development of ED [241]. One review found incidence of BD to be 4.7 times higher in individuals with BN, 3.6 times higher in individuals with BED and 3.5 times higher for binge/purge ED subtypes overall. Due to the low prevalence of AN and BD in the general population, an accurate estimation of this comorbidity is difficult to obtain [241, 243]. BD in individuals with ED is associated with increased severity of core symptoms including body dissatisfaction, weight/shape concern, eating concern, impulse regulation, interoceptive awareness and perfectionism [244]. Mood instability is also significantly higher in individuals with a BD/ED comorbidity compared to those with BD alone. Systematic review of BD and its clinical correlates by McDonald et al. [245] suggests this finding indicates shared aetiology between ED and BD through emotional dysregulation.

### ADHD and autism spectrum disorders

There is an emerging body of literature exploring associations between EDs and attention-deficit hyperactivity disorder (ADHD) and autism spectrum disorders (ASDs), however few have examined the conditions as risk factors in the development of ED. A 2016 meta-analysis of twelve studies found a three-fold increased risk of ED among individuals with ADHD [246]. Similarly, a 2020 matched cohort screening study found the same three-fold increase—almost one third of children and adolescents with ADHD were at risk of ED, compared to 12% of healthy controls. Here, BMI was a statistically significant predictor of risk [247]. Impulsivity and inattention symptoms of childhood ADHD have been positively associated with the development of overeating and bulimic-type behaviours in adolescence [248]. A longitudinal study of a large sample of adolescents reported that the onset of emotional and behavioural issues, including those associated with ADHD and conduct disorder, was observed to occur prior to the onset of disordered eating behaviours [249].

A 2013 systematic review found elevated rates of ASDs in ED populations compared with healthy controls, however, six of the eight studies in this review were based on longitudinal research using the same community sample [250]. The authors suggested a need to integrate appropriate, well-structured ASD assessment tools into routine care of ED service users, with the prevalence of ASD traits potentially contributing to ‘high treatment resistance to conventional therapies’ [250]. Dell’ Osso et al. [251] tested such an instrument in a sample of 138 individuals meeting DSM-5 criteria for an ED and 160 controls. They found significantly higher autism spectrum traits in participants with EDs, particularly verbal and non-verbal communication, inflexibility and adherence to routine, and restricted interest and rumination. Individuals with restrictive EDs were more likely to display ASD traits. Similarly, as part of a large, population-based prospective study of women and their children, Schaumberg et al. (2021) found autistic-like social communication difficulties during middle childhood were associated with BN symptoms during adolescence in both males and females [252]. They also discovered that misattribution of faces as sad or angry at 8.5 years of age was associated with a diagnosis of AN and purging behaviours at age 14. Contrarily, Dinkler et al. [253] in their prospective twin cohort study found no association between traits of autism in nine-year-old children and a later AN diagnosis, as well as noting a marked elevation in restricted/repetitive behaviour and interests *only* in the subgroup of individuals with acute AN. They questioned previous reports of elevated prevalence of ASD in AN and instead wondered if autistic traits may be best conceptualised as an epiphenomenon of the acute phase of AN.

### Post-traumatic stress disorder

Although there is a large body of evidence relating to childhood trauma and abuse as a risk factor for the development of ED, few studies were identified investigating the role of post-traumatic stress disorder (PTSD) specifically as a risk factor. No distinction was made in the search methodology for this review between complex trauma and early childhood adverse events, with all studies captured under the search term ‘risk factors.’ Studies presented in this section, focused on the link between diagnosed PTSD and development of ED.

Results from two cohort studies observed an association between PTSD and severity of ED symptoms as well as relatively high prevalence rates within sample populations [254, 255]. Among a patient sample in Sweden who had experienced trauma either prior to ED onset, after onset or within a year of onset, lifetime prevalence of PTSD was observed to be 24.1% [255]. An almost identical PTSD prevalence was found within a smaller ethnically diverse sample of obese women with BED in the US, at 24% [254]. Analysis of the impact of timing of trauma exposure on ED symptom severity in the Swedish sample found the association was only significant in the group who had experienced trauma in the same year of their ED diagnosis [255]. This analysis was not undertaken in the US study. Brewerton et al. [256] assessed adults entering ED treatment at seven US sites and found 49.3% had PTSD. It was found that individuals who were significantly more symptomatic had a higher propensity towards binge-type disorders and reported worse quality of life than those without PTSD. Co-occurrence of PTSD and AN was reported by Reyes-Rodriguez et al. (2011) as part of their cross-sectional study of 753 women with AN. They found 13.7% of the sample of AN patients also met criteria for PTSD with childhood sexual traumas being the most common traumatic event associated with the diagnosis [257].

Evidence from three studies relating to EDs in veteran populations—a meta-analysis (Barlett and Mitchell [259]); a retrospective chart review (Forman-Hoffman et al. [258]); and a retrospective cohort study of female veterans (Mitchell et al. [260])—found an association between increased ED prevalence and PTSD and trauma. Through a telephone interview with 1004 veterans, Formann-Hoffman et al. [258] determined that 16% of their sample had a lifetime ED with many of the cases also experiencing comorbid PTSD or lifetime sexual trauma. However, increased risk for ED among the veteran population could not be solely attributed to trauma, as unhealthy weight control behaviours are also common in this population due to strict weight and fitness requirements within the military [259–261].

### Summary

The prevalence of personality traits appear to differ according to the ED diagnostic category. Elevated levels of perfectionism are common amongst AN and BN, obsessiveness strongly associated with AN, and binge/purge presentations consistently associated with impulsivity and greater emotional dysregulation, whereas lack of emotional awareness is not ED specific and common amongst most ED diagnoses [178–183, 196]. Although co-occurrence of ED and personality disorders has been consistently identified in studies of comorbidity (e.g., BPD and binge/purge EDs), mood and anxiety disorders represent the most common psychiatric comorbidities in individuals with EDs (e.g., MDD affects over 75% of binge/purge EDs, SAD affects 42% of adults with an ED) [212, 220, 223, 225–227]. There is also good evidence to suggest that the presence of a diagnosable childhood anxiety disorder (e.g., OCD) precedes the onset of an ED later in life [177, 211]. Other psychological factors which appear to contribute to the risk of EDs include diagnoses of PTSD, ADHD, or ASD [246, 250, 254].

## 5. Gender differences

EDs impact a higher number of females with greater symptom severity. While common risk factors are shared across genders, such as low self-esteem and high shape/weight concern, males have been identified as less likely to engage in severe dieting behaviours compared with their female counterparts [262, 263].

### Onset

Puberty is a period of significant risk for ED development in both males and females. Research has implicated increased production of sex hormones during puberty, in particular estrogen, in the onset of EDs [264]. Evidence has consistently demonstrated that early onset of puberty is strongly associated with increased risk for ED development in both young males and females. Favaro et al. [265] linked earlier age of menarche with a younger mean age of onset of AN and BN. It has been suggested that if an individual experiences changes to their body shape, associated with menarche, at an earlier time than their peers, this may lead to heightened body dissatisfaction and which in turn may contribute to early the onset of EDs.

Despite the commonality between males and females in terms of the risk of ED development posed by puberty, it had been suggested that bodily changes experienced during this time possess a stronger impact for females as compared to males. It is thought that changes to one’s body shape move females further away from the thin ideal, whereas the changes for males move them closer to ideals around muscularity [266]. These findings have been supported by a cohort study, which found that bulimic symptoms and body dissatisfaction were associated with early puberty in females and late puberty in males [267]. Similarly, having a higher BMI comparative to peers has been associated with ED risk among teenage girls but not boys in a US school cohort [265].

### Comorbidity

Research into gender differences has found that an equal proportion of male and female adolescents with an ED experience comorbid anxiety or depression [268]. A further four-year retrospective study in male adolescents with a diagnosed ED supported the assertion that comorbid anxiety and depression posed considerable ED risk to males [269]. Research has also identified increased prevalence of compulsive disorders, including gambling and substance use, among males as compared to females in a cohort of individuals at risk of ED [270]. While male ED risk has been associated with compulsive and depressive symptoms in these studies, evidence presented in a longitudinal study of adolescents found depression to be associated with higher ED symptomology in 12-year-old girls but not in boys [271]. Further research into EDs and depression in males is required to clarify the impact of this association.

### Gender roles

Gender roles have been investigated as a potential contributor to ED risk. Exposure to media ideals has been found to be associated with increased body dissatisfaction and ED symptomology in university-aged males [272]. Research has also indicated that increased femininity in heterosexual males is negatively associated with muscle dissatisfaction [273]. Weak associations have also been found between femininity in women and eating pathology and body satisfaction. Among both sexes, masculinity was found to have a significant negative relationship with eating pathology, also conferring modest protection to body dissatisfaction [273].

Interactions between societal gender roles and sexual orientation is also known to play a role in ED risk with researchers suggesting that greater social body image pressures are present among gay males. A systematic review of disordered eating among sexual minority individuals has reported that elevated ED symptomology exists across all LGBTQI + groups as compared to heterosexual males and females [274]. A further study of men aged 18 to 35 found that disordered eating and body dissatisfaction was higher in gay and bisexual men compared to heterosexual men, as was susceptibility to social messaging around body image [275]. The occurrence of body image disorders has also been found to be higher among sexual minorities as compared to heterosexual samples [276]. A recent study involving a sample of transgender and gender non-binary individuals reported that increased internalised transphobia was associated with increased likelihood of disordered eating symptoms [277]. There is insufficient evidence currently available to separate risk of engagement in specific types of ED behaviours according on sexual identity [274].

### Summary

The literature indicates that whilst both males and females are susceptible to risk factors for EDs such as early puberty onset and elevated weight/shape concerns, it appears that these factors have a stronger impact upon females as compared to males in terms of risk of developing disordered eating behaviours and psychopathology (e.g., severe dieting, bulimic symptoms and body dissatisfaction) [292, 294, 297–300]. Recent findings also indicate that LGBTQI + groups are at a higher risk of ED symptomology and body image disorders as compared to heterosexual individuals [305–307].

## 6. Socio-economic status

Despite the pervasive view that EDs disproportionately affect more affluent groups, evidence suggests that disordered eating behaviours occur at similar rates across all income levels and regardless of employment status [278]. Differences between socio-economic status (SES) seem to emerge in the types of disordered eating. Specifically, a positive correlation has been reported between non-fulltime workers and binge eating and purging behaviours. Also, a trade or certificate qualification has been shown to be positively associated with strict dieting as compared to groups with no higher education [278]. In contrast, a large study conducted in Sweden failed to find a relationship between social class and household income and incidence of EDs in females. However, in males, lower household income was associated with increased risk of BN and EDNOS, although the study observed a very low rate of BN in males [279].

Recent studies in the US have found low food security to be a predictor for disordered eating behaviours [280]. Among higher SES adolescents, binge eating behaviours were associated with weight-related teasing by family members [281]. In an adult sample, experience of low food security was more common among individuals with BN and BED as compared to healthy weight controls [282]. Lower food security in these individuals was associated with more frequent binge eating episodes and, in individuals with BN, unhealthy compensatory behaviours [282].

High levels of parental education have also been identified as a predictor of EDs [119, 283]. Higher educational attainment by both parents as well as maternal grandparents has been associated with higher incidence of AN, BN, and EDNOS equally across diagnoses in females [279, 284]. In males a positive association was found between parental education and AN, but not for BN or EDNOS [279].

### Summary

Research into sociocultural risk factors for EDs suggests that income has little impact on overall ED risk although available evidence points to specific indicators that have an influence [278]. Higher education attainment is associated with restrictive ED behaviours, while experience of food insecurity is associated with binge-type behaviours and EDs [279, 282, 284].

## 7. Ethnic minority

Although there is no evidenced association between ethnic background and the risk of ED onset, specific aspects of ED psychopathology do appear to differ between ethnic groups [285, 286]. A cohort study of females aged between nine and 22 years old found those with an ED were more likely to be non-Hispanic White, come from well-educated households, and be well-educated themselves [287]. A recent study of a treatment-seeking community sample in US found that Black individuals displayed higher rates of BED as compared to other ethnic groups, however overall Asian and Black individuals were less likely to report ED symptoms than White individuals [288]. Significantly higher thin ideal internalisation has been observed among Asian-American participants as compared with other groups [285]. Additionally, the association between fear of losing control of eating and depressive symptoms has been found to be stronger in Asian and Pacific Islander minorities than other ethnic groups [289]. In a study comparing thin-ideal internalisation among young Australian and Malaysian women, a stronger association between body dissatisfaction and restrained eating practices was observed in the Australian sample [286].

Further investigation of ethnic minority status has implicated perceived ethnic discrimination as a risk factor in ED development. In a cohort of college students, perceived discrimination based upon one’s ethnicity was associated with increased prevalence of key ED symptoms including restraint, weight/shape concern, body dissatisfaction and bulimia [290]. Perceived discrimination was also found to increase drive for muscularity among males in the sample but not drive for thinness among females. These findings indicate a potentially growing risk for ED in CALD individuals [290].

### Summary

A small body of evidence was identified in the current RR regarding the association between ethnic minority status and ED risk. Of the studies reviewed, unique associations have been found between particular ethnic groups and specific aspects of ED psychopathology. For example, in comparison to other ethnic groups, higher rates of BED have been observed in Black-Americans and greater thin ideal internalisation in Asian-Americans [286, 289]. Given that a significant proportion of ED research has been conducted using White/Caucasian participants, greater research efforts are needed to better understand the features of EDs in ethnically diverse groups.

## 8. Body image and social influence

Weight/shape concern, overvaluation of weight/shape and drive for thinness, referred to here using the term body image concerns, are key concepts in ED [291, 292]. Along with the social and cultural factors that contribute to body image concerns, these concerns have been extensively investigated as risk factors for the development of EDs. Research in this area has been concentrated among women and girls whose body image concerns are characterised by a focus on low body weight and the thin-ideal [293], but greater recent focus on men and boys with regard to the muscular/lean ideal has been seen due to increasing recognition of muscle orientated EDs in males. Engagement with particular environments that shape social norms for appearance and promote pursuit of the ideal body shape or weight, or involvement in certain activities with a culture of strict dieting and excessive exercise is encouraged, such as college level or professional sports, are also well studied risk factors in ED literature.

### Body image and appearance ideals

Studies using prospective designs have found evidence for body image concerns predicting development of EDs and ED behaviours. In an eight-year longitudinal study of adolescent girls, higher levels of perceived pressure to be thin, thin-ideal internalisation, and body dissatisfaction were significant predictors of later onset ED (BN, BED, and purging disorder) [294]. Among an adolescent sample, dissatisfaction with weight and shape, but not overvaluation or preoccupation, was a predictor of onset of an ED after 12 months [295]. The authors suggest that while body dissatisfaction may impart risk for ED development, the other body image-related constructs of overvaluation and preoccupation, may indicate presence of ED psychopathology. A systematic review of the impact of anti-obesity public health messages has found that endorsement of thin ideals and drive for thinness are exacerbated in response to exposure to messages which are stigmatising towards individuals who are overweight or obese [296]. In a large longitudinal sample of adolescent boys and girls, body image concerns predicted binge eating over 5 years to young adulthood [297] and persistent disordered eating 10 years later among both males and females [298], and body dissatisfaction, preoccupation with body weight and shape, and overvaluation predicted increases in disordered eating 15 years later, particularly in females [299]. Similarly, in a cohort of this sample characterised as having BMI in the overweight category, higher body image concerns predicted prevalence and onset of disordered eating (binge eating and extreme weight control behaviours) over five years [300]. Findings for body image concern as a risk factor for development of AN are mixed. In this regard, a systematic review of 46 longitudinal studies by Glashouwer et al. [301] with a pooled sample of 4,928 patients with AN was unable to definitively determine whether body dissatisfaction was a causal factor in disorder onset.

### Media, social media, and the internet

The impact of media depictions of appearance ideals on ED symptoms have been examined with studies of varying methodologies. A meta-analysis of laboratory-based experimental studies found that viewing idealised images resulted in a small but non-significant increase in body dissatisfaction. However, exposure to these images was found to have a greater impact on groups considered at high-risk for developing EDs [302]. Of note, there were no differences observed in the impact of these images based on gender, indicating that men and women are equally affected by media portrayals of idealised bodies [302].

Among 574 women aged between 14 and 36, social expectations to be thin were found to mediate the relationship between protective self-presentation and disordered eating [303]. This finding aligns with research on exposure to negative parental attitudes regarding weight to be a risk factor in the later development of ED, discussed previously [118, 303].

As with traditional media, the effects of portrayal of idealised bodies on the internet and on social media has been explored. Among young women, use of social media was found to impact weight and shape concerns [304] and among a predominantly female sample of participants with AN, use of appearance-focused social media was found to be associated with higher levels of ED symptoms [305]. A systematic review found that general internet use was associated with body image and eating concerns [306]. Further exploration of problematic internet use suggested excessive use of social media was associated with increased risk of AN and BN, while video gaming was associated with risk of BED [307]. However, recent proliferation of pro-AN or pro-ED websites and social media networks may create online environments that are more detrimental to the health of individuals at risk of ED than other forms of media. Even among females with normal BMI and no history of ED, one week of exposure to pro-ED website content resulted in a significant reduction (20%) in calorie intake among participants compared to groups who were exposed to other website content including health and fitness websites [308]. Dangers associated with pro-ED websites is not restricted to females, with a content analysis study finding that up to 25% of participants on pro-AN forums are male, suggesting that these sites may have a substantive negative impact with males engaged with these sites expressing negative experiences including body dissatisfaction [309].

### Summary

Body image concerns are a well-known risk factor for EDs. High levels of body dissatisfaction and internalisation of the thin ideal have been found to be predictors of ED onset, whereas related constructs of overvaluation and preoccupation with weight and shape are considered to reflect current ED psychopathology [270–277]. Exposure to the thin ideal via either traditional media or social media is associated with greater risk of an ED, with evidence suggesting that both males and females are equally impacted by this content [278–283].

## 9. Elite sports, female athlete triad, and excessive exercise

Engagement in activities that accept or promote strict dieting practices and endorsement of low body fat has the potential to contribute to development and maintenance of ED symptoms [310]. Consistent with this, EDs among elite and college/university level athletes were observed at higher rates than in non-athlete comparison groups [311], although no difference in prevalence of EDs was found between athletes engaged in sports with an emphasis on aesthetics and/or weight and athletes engaged in sports without this focus. The female athlete triad (FAT), characterised by low energy availability (through increased physical activity or dietary restriction), amenorrhea and low mineral bone density, is considered a consequence of training for elite level sports and pursuit of lean physiques [312]. Features of FAT have also been observed in elite para-athletes (n = 260) with no difference in risk between genders or sport type [313].

In relation to ED behaviours, among elite athletes (n = 224), high prevalence of clinically significant ED symptomology (22.8%) has also been found [314].Similarly, in a sample of college level female gymnasts and swimmers (n = 325), 4.6% (n = 15) engaged in intentional vomiting, 1.5% (n = 5) used laxatives and 2.5% (n = 8) used diuretics for weight control. Additionally, 10.5% (n = 3.4) engaged in binge eating two or more times a week, while almost all participants engaged in binge eating once a week, 96.6% (n = 314) [315]. However, in a smaller UK sample of male and female gymnasts (n = 51) no purging behaviours were observed, although 31% of male gymnasts in this group scored highly on ED self-report questionnaires [316].

However, other studies have not found these differences between athlete and non-athlete groups. For example, a cohort study comparing elite and non-elite athletes to controls (n = 725) was also unable to find any differences between the three groups in terms of ED behaviours. However, it did highlight distinct differences associated with social pressures and influences on body image and weight in athletes versus non-athletes. There is some evidence to suggest that unlike female athletes, male athletes are not at greater risk of developing EDs than non-athletes [317]. Evidence from a meta-analysis of 31 studies of ED athletes indicated that, with the exception of wrestling, male athletes were not at greater risk of disordered eating than non-athletes. Although, researchers noted that studies were heterogenous and measurements were impacted by the potential inappropriateness of ED assessment tools for male populations [318].

Among non-elite populations, recognising excessive physical activity or exercise levels among women in the community is particularly important in risk assessment of ED, as these individuals were found to be 2.5 times as likely to have an ED diagnosis than non-excessively exercising individuals [319]. Furthermore, participation in activities promoting lean body types such as yoga and pilates has also been highlighted as a potential risk factor for ED development. However, in a large cohort study (n = 2,287) of young adults no association was found between participating in yoga and pilates and ED symptomology among female subjects but increased risk of unhealthy and extreme weight control behaviours as well as binge eating was observed in males [320]. Further research is required to understand the unique associations identified in this study.

Similar to athletic settings, other physical activity pursuits take place in environments that may promote ED symptoms. A systematic review and meta-analysis observed higher rates of ED among dancers, where dancers were found to have three times greater risk of having AN or EDNOS but not BN, than the general population and risk was particularly heightened among ballet dancers [321].

### Summary

Involvement in elite sports is a potential risk factor for disordered eating behaviours among both male and female athletes [311–317]. Increased attention should be paid towards excessive exercise by non-elite populations in the community as risk factor for EDs and to support screening and early intervention activities [318–320].

## Discussion

This review to aimed to summarise recent peer-reviewed evidence relating to risk factors associated with EDs. An extensive number of research studies were identified, exploring a multitude of risk factors. For the purposes of this review, the research findings were broadly characterised into nine primary categories: (1) genetics, (2) gastrointestinal microbiota and autoimmune reactions, (3) childhood and early adolescent experiences, (4) personality traits and comorbid mental health conditions, (5) gender, (6) socio-economic status, (7) ethnic minority, (8) body image and social influence, (9) and elite sports.

Identification of the recent evidence relating to key risk factors offers valuable knowledge to researchers, clinicians, and policy makers, such that it may inform the development of evidence-based approaches for the care and treatment of individuals with EDs. An understanding of risk factors is essential for the development and refinement of aetiological models [8]. In a recent review of existing models of disordered eating, Pennesi and Wade [21] reported that very few of the existing theoretical models (18.5%) have informed the development of effective interventions. The authors call upon researchers to use empirically supported risk-factors to modify existing theories, which then can inform prevention and treatment interventions [21].

The findings of the current review can be used to determine which risk factors are differentially appropriate targets for prevention, early intervention, and/or treatment efforts [322]. For example, modifiable risk factors such as negative parental comments towards weight and eating behaviours may be best approached using targeted prevention parenting programs to assist with modelling of healthy eating patterns and family dialogue. There is evidence to suggest targeted prevention programs addressing early signs of disordered eating in adolescents (e.g., the *Body Project, StudentBodies2-BED*) are effective in significantly reducing future onset of EDs [323, 324]. They represent a targeted, efficient way of addressing modifiable risk factors rather than approaching the population as a whole in a largely non-specific manner.

Identifying risk factors which are less amenable to modification, such as genetic risk factors and autoimmune conditions, may represent an opportunity for enhanced screening measures to recognise early signs of disordered eating prior to onset of full ED diagnosis. Research has identified low levels of screening and poor detection rates of EDs by health practitioners, in particular non-stereotypical presentations of EDs in primary care settings [325–327]. A noteworthy outcome of the current review pertains to the growing field of evidence supporting increased risk of EDs within the sexual minority groups as compared to heterosexual samples. Given the high levels stigma surrounding both LGBTQI and EDs, particularly for young males, it is of particular importance that clinicians thoroughly assess for disordered eating behaviours within sexual minority groups [328, 329]. Accordingly, the findings of this review may offer an opportunity for advances in the development of resources (e.g., screening instruments) to assist practitioners in recognising evidenced risk factors for EDs.

Finally, awareness of comorbid psychiatric illnesses or personality traits may inform targets for treatment interventions, including as specific programs for individuals with comorbid personality disorders and ED. Enhanced Cognitive Behaviour Therapy (CBT-e) offers an example of the way in which comorbid psychological traits, considered to be “external” to the ED itself, can be addressed to create a more efficacious, tailored treatment for patients [330]. The inclusion of additional treatment targets to address comorbid psychological mechanisms (clinical perfectionism, core low self-esteem, and interpersonal problems) allows for cognitive behaviour therapy treatment to meet the needs of non-responders for whom comorbid psychopathology may have interfered with their treatment response [331].

Additionally, given the search strategy of the review adopted a timeline which overlaps between two versions of the Diagnostic and Statistical Manual of Mental Disorders [332], namely Version 4 and 5 (i.e., DSM-IV and DSM-5), our findings were able to highlight inconsistences in the degree of research conducted across various ED diagnoses. In particular, the findings demonstrate that considerably less is known about the risk factors associated with EDs which were recently included as formal diagnoses in the DSM-5, including ARFID, BED, rumination disorder, and pica, highlighting the need for more focused research efforts to be put towards these diagnoses.

In this review, gaps in the existing literature were identified. Many of the research studies included in the review adopted a cross-sectional study design and therefore focused upon associations and correlations between EDs and potential risk factors. Consequently, some studies were limited in their capacity to delineate temporal or causal relationships, or how in fact the associations connect the factor with the illnesses. For example, although an understanding of psychiatric comorbidities of EDs (e.g., perfectionism, impulsivity etc.) provides value, without longitudinal research it is difficult to disentangle whether these traits contribute to ED onset or are symptoms of it. Similarly, identification of trauma and abuse as a risk factor for eating disorders needs further clarification as this association has been described for many other mental health conditions such as anxiety and depression [333], and is not likely a specific association to eating disorders. Additionally, several of the studies included in the current review were not able to distinguish between factors related to onset and factors related to maintenance in EDs, which represents an important differentiation of different classes of risk factors and their influence [207]. It is possible that some of the constructs reviewed in the present paper have a role as maintenance factors, even if they may not have a role as a causal risk factor. An understanding of whether one psychiatric condition precedes another can assist clinicians in treatment planning and inform sequencing of treatment targets. Taken together, these considerations represent a limitation in our ability to understand the implications of these identified risk factors. For risk factors which have relied heavily upon cross-sectional studies, future research is encouraged to adopt experimental or prospective study designs to better capture the nature of the variable being examined.

Several of the studies included in the review examined risk factors in isolation from one another and thus assessment of their association with EDs occurred as though they were independent contributors of risk. This is markedly distinct from real world environments in which EDs develop in response to a multitude of risk factors and consequently, weakens the ecological validity of the reported findings. An understanding of the ways in which various risk factors interact with each other (e.g., whether they are cumulative in nature), is necessary to form a detailed conceptualisation of illness profiles for both clinicians and researchers, which can in turn inform the development of targeted interventions. Conversely, in the absence of this information, the mechanisms of change are less clear. Future research would benefit from adopting an approach towards risk factors as co-occurring, interactional variables as opposed to a siloed view.

Given the attempt to summarise peer-reviewed ED literature in a broad-reaching and prompt manner, there are some limitations of the review. First broad search terms, required to fulfil the purpose of the large series of rapid reviews, of which this paper forms part, were used to collate evidence, which may have compromised the specificity of the included studies for individual ED diagnoses and/or phenotypes and individual risk factors. Additionally, research studies were excluded if they reported on unpublished data, implementation research, or if they were observational studies; and included studies were mostly limited to those conducted in Western cultures with high-resource health systems. Finally, having a specified time period for the review meant that seminal studies conducted prior to the start date were not included.

## Conclusions

This review has identified risk factors for which a substantial evidence-base exists as well as emerging areas requiring further investigation (e.g., ADHD) and ED diagnoses where there is less available evidence (e.g., BED, ARFID). A broad review of the literature has been provided, however future studies are required which critique the strength of evidence of the causal nature of these risk factors.

## Supplementary Information

**Additional file 1**. PRISMA Diagram & Included Studies Table.

## Data Availability

Not applicable—all citations provided.

## Abbreviations

ED: Eating disorder
BN: Bulimia nervosa
AN: Anorexia nervosa
BED: Binge eating disorder
AN-R: Anorexia nervosa (restrictive subtype)
ARFID: Avoidant restrictive food intake disorder
AN-BP: Anorexia nervosa (binge-purge subtype)
EDNOS: Eating disorder not otherwise specified
A-AN: Atypical anorexia nervosa
BMI: Body mass index
NES: Night eating syndrome
ADHD: Attention-deficit/hyperactivity disorder
ASD: Autism spectrum disorder
DBT: Dialectical behaviour therapy
BPD: Borderline personality disorder
MDD: Major depressive disorder
SAD: Social anxiety disorder
BD: Bipolar disorder
PTSD: Post-traumatic stress disorder
SES: Socioeconomic status
CALD: Culturally and linguistically diverse
FAT: Female athlete triad

## Footnotes

1 *having three different alleles at the same locus.*

2 *Polymorphism is a DNA sequence variation.*

3 *perception or awareness of sensations inside the body.*

4 *DNA methylation is a process that controls the expression/suppression of a gene without changing the genetic sequence.*

5 *Small molecules formed in or necessary for metabolism.*

6 *Models using rates and mice.*

## References

[1] Jenkins PE, Hoste RR, Meyer C, Blissett JM (2011). Eating disorders and quality of life: a review of the literature. Clin Psychol Rev.

[2] Stice E, Marti CN, Rohde P (2013). Prevalence, incidence, impairment, and course of the proposed DSM-5 eating disorder diagnoses in an 8-year prospective community study of young women. J Abnorm Psychol.

[3] Crow SJ, Smiley N. Costs and Cost-Effectiveness in Eating Disorders. The Oxford Handbook of Eating Disorders [Internet]. 2010 Jul 6 [cited 2022 Feb 14]; Available from: https://www.oxfordhandbooks.com/view/10.1093/oxfordhb/9780195373622.001.0001/oxfordhb-9780195373622-e-026

[4] Striegel-Moore RH, Bulik CM (2007). Risk factors for eating disorders. Am Psychol.

[5] Keel PK, Forney KJ (2013). Psychosocial risk factors for eating disorders. Int J Eat Disord.

[6] Hilbert A, Pike KM, Goldschmidt AB, Wilfley DE, Fairburn CG, Dohm FA (2014). Risk factors across the eating disorders. Psychiatry Res.

[7] Austin SB (2000). Prevention research in eating disorders: theory and new directions. Psychol Med.

[8] Stice E, South K, Shaw H (2012). Future directions in etiologic, prevention, and treatment research for eating disorders. J Clin Child Adolesc Psychol.

[9] Hamilton A, Mitchison D, Basten C, Byrne S, Goldstein M, Hay P (2021). Understanding treatment delay: perceived barriers preventing treatment-seeking for eating disorders. Aust N Z J Psychiatry.

[10] Hart LM, Granillo MT, Jorm AF, Paxton SJ (2011). Unmet need for treatment in the eating disorders: a systematic review of eating disorder specific treatment seeking among community cases. Clin Psychol Rev.

[11] Rowe E (2017). Early detection of eating disorders in general practice. Aust Fam Physician.

[12] Bailey AP, Parker AG, Colautti LA, Hart LM, Liu P, Hetrick SE (2014). Mapping the evidence for the prevention and treatment of eating disorders in young people. J Eat Disord.

[13] Cororve Fingeret M, Warren CS, Cepeda-Benito A, Gleaves DH (2006). Eating disorder prevention research: a meta-analysis. Eat Disord.

[14] Bulik CM, Berkman ND, Brownley KA, Sedway JA, Lohr KN (2007). Anorexia nervosa treatment: a systematic review of randomized controlled trials. Int J Eat Disord.

[15] Kazdin AE, Fitzsimmons-Craft EE, Wilfley DE (2017). Addressing critical gaps in the treatment of eating disorders. Int J Eat Disord.

[16] Steinhausen HC (2009). Outcome of eating disorders. Child Adolesc Psychiatr Clin N Am.

[17] Silén Y, Sipilä PN, Raevuori A, Mustelin L, Marttunen M, Kaprio J, Keski-Rahkonen A (2021). Detection, treatment, and course of eating disorders in Finland: a population-based study of adolescent and young adult females and males. Eur Eat Disord Rev.

[18] Le Grange D, Hughes EK, Court A, Yeo M, Crosby RD, Sawyer SM (2016). Randomized clinical trial of parent-focused treatment and family-based treatment for adolescent anorexia nervosa. J Am Acad Child Adolesc Psychiatry.

[19] Lock J, Grange DL (2019). Family-based treatment: Where are we and where should we be going to improve recovery in child and adolescent eating disorders. Int J Eat Disord.

[20] Wonderlich SA, Bulik CM, Schmidt U, Steiger H, Hoek HW (2020). Severe and enduring anorexia nervosa: update and observations about the current clinical reality. Int J Eat Disord.

[21] Pennesi JL, Wade TD (2016). A systematic review of the existing models of disordered eating: Do they inform the development of effective interventions?. Clin Psychol Rev.

[22] InsideOut Institute for Eating Disorders. Australian Eating Disorders Research and Translation Strategy 2021–2031. Sydney; 2021.

[23] Virginia Commonwealth University. Research Guides: Rapid Review Protocol [Internet]. Rapid Review Protocol. [cited 2021 Jun 19]. Available from: https://guides.library.vcu.edu/c.php?g=240398&p=1598530

[24] Brooks SK, Webster RK, Smith LE, Woodland L, Wessely S, Greenberg N (2020). The psychological impact of quarantine and how to reduce it: rapid review of the evidence. The Lancet.

[25] World Health Organisation. WHO | Rapid reviews to strengthen health policy and systems: a practical guide [Internet]. WHO. World Health Organization; [cited 2021 Jun 19]. Available from: http://www.who.int/alliance-hpsr/resources/publications/rapid-review-guide/en/.

[26] Canadian Agency for Drugs and Technologies in Health. About the Rapid Response Service | CADTH [Internet]. [cited 2021 Jun 19]. Available from: https://www.cadth.ca/about-cadth/what-we-do/products-services/rapid-response-service.

[27] Hamel C, Michaud A, Thuku M, Skidmore B, Stevens A, Nussbaumer-Streit B (2021). Defining rapid reviews: a systematic scoping review and thematic analysis of definitions and defining characteristics of rapid reviews. J Clin Epidemiol.

[28] Moher D, Liberati A, Tetzlaff J, Altman DG, TP Group (2009). Preferred reporting items for systematic reviews and meta-analyses: the prisma statement. PLOS Med.

[29] Bettany-Saltikov J (2016) How to do a Systematic Literature Review in Nursing: a step-by-step guide. Open University Press: London, England

[30] Rethlefsen ML, Kirtley S, Waffenschmidt S, Ayala AP, Moher D, Page MJ (2021). PRISMA-S: an extension to the PRISMA statement for reporting literature searches in systematic reviews. Syst Rev.

[31] Aouad P, Bryant E, Maloney D, Marks P, Le A, Russell H (2022). Informing the development of Australia’s National Eating Disorders Research and Translation Strategy: a rapid review methodology. J Eat Disord.

[32] Kraemer HC, Stice E, Kazdin A, Offord D, Kupfer D (2001). how do risk factors work together? Mediators, moderators, and independent, overlapping, and proxy risk factors. AJP.

[33] Thornton LM, Mazzeo SE, Bulik CM, Adan RAH, Kaye WH (2011). The heritability of eating disorders: methods and current findings. behavioral neurobiology of eating disorders.

[34] Bould H, Sovio U, Koupil I, Dalman C, Micali N, Lewis G (2015). Do eating disorders in parents predict eating disorders in children? Evidence from a Swedish cohort. Acta Psychiatr Scand.

[35] Breithaupt L, Hubel C, Bulik CM (2018). Updates on genome-wide association findings in eating disorders and future application to precision medicine. Curr Neuropharmacol.

[36] Bulik CM, Thornton LM, Root TL, Pisetsky EM, Lichtenstein P, Pedersen NL (2010). Understanding the relation between anorexia nervosa and bulimia nervosa in a Swedish national twin sample. Biol Psychiat.

[37] Mazzeo SE, Mitchell KS, Bulik CM, Reichborn-Kjennerud T, Kendler KS, Neale MC (2009). Assessing the heritability of anorexia nervosa symptoms using a marginal maximal likelihood approach. Psychol Med.

[38] Watson HJ, Yilmaz Z, Thornton LM, Hübel C, Coleman JRI, Gaspar HA (2019). Genome-wide association study identifies eight risk loci and implicates metabo-psychiatric origins for anorexia nervosa. Nat Genet.

[39] Baker JH, Maes HH, Lissner L, Aggen SH, Lichtenstein P, Kendler KS (2009). Genetic risk factors for disordered eating in adolescent males and females. J Abnorm Psychol.

[40] Keski-Rahkonen A, Neale BM, Bulik CM, Pietiläinen KH, Rose RJ, Kaprio J (2005). Intentional weight loss in young adults: sex-specific genetic and environmental effects. Obes Res.

[41] Versini A, Ramoz N, Le Strat Y, Scherag S, Ehrlich S, Boni C (2010). Estrogen receptor 1 gene (ESR1) is associated with restrictive anorexia nervosa. Neuropsychopharmacology.

[42] Yao S, Kuja-Halkola R, Martin J, Lu Y, Lichtenstein P, Norring C (2019). Associations between attention-deficit/hyperactivity disorder and various eating disorders: a Swedish nationwide population study using multiple genetically informative approaches. Biol Psychiat.

[43] Capusan AJ, Yao S, Kuja-Halkola R, Bulik CM, Thornton LM, Bendtsen P (2017). Genetic and environmental aspects in the association between attention-deficit hyperactivity disorder symptoms and binge-eating behavior in adults: a twin study. Psychol Med.

[44] Duncan L, Yilmaz Z, Gaspar H, Walters R, Goldstein J, Anttila V (2017). Significant locus and metabolic genetic correlations revealed in genome-wide association study of anorexia nervosa. Am J Psychiatry.

[45] Yilmaz Z, Szatkiewicz JP, Crowley JJ, Ancalade N, Brandys MK, van Elburg A (2017). Exploration of large, rare copy number variants associated with psychiatric and neurodevelopmental disorders in individuals with anorexia nervosa. Psychiatr Genet.

[46] Wade TD, Fairweather-Schmidt AK, Zhu G, Martin NG (2015). Does shared genetic risk contribute to the co-occurrence of eating disorders and suicidality?. Int J Eat Disord.

[47] Thornton LM, Welch E, Munn-Chernoff MA, Lichtenstein P, Bulik CM (2016). Anorexia nervosa, major depression, and suicide attempts: shared genetic factors. Suicide Life Threat Behav.

[48] Zhang R, Kuja-Halkola R, Birgegård A, Larsson H, Lichtenstein P, Bulik CM (2021). Association of family history of schizophrenia and clinical outcomes in individuals with eating disorders. Psychol Med.

[49] Hübel C, Abdulkadir M, Herle M, Loos RJF, Breen G, Bulik CM (2021). One size does not fit all. Genomics differentiates among anorexia nervosa, bulimia nervosa, and binge-eating disorder. Int J Eat Disord..

[50] Kirk KM, Martin FC, Mao A, Parker R, Maguire S, Thornton LM (2017). The anorexia nervosa genetics initiative: study description and sample characteristics of the Australian and New Zealand arm. Aust N Z J Psychiatry.

[51] Sardahaee FS, Holmen TL, Micali N, Kvaløy K (2017). Effects of single genetic variants and polygenic obesity risk scores on disordered eating in adolescents—The HUNT study. Appetite.

[52] Kaye WH, Wierenga CE, Bailer UF, Simmons AN, Bischoff-Grethe A (2013). Nothing tastes as good as skinny feels: the neurobiology of anorexia nervosa. Trends Neurosci.

[53] Gratacòs M, Escaramís G, Bustamante M, Saus E, Agüera Z, Bayés M (2010). Role of the neurotrophin network in eating disorders’ subphenotypes: body mass index and age at onset of the disease. J Psychiatr Res.

[54] Nicoletti CF, Delfino HBP, Ferreira FC, de Pinhel MAS, Nonino CB (2019). Role of eating disorders-related polymorphisms in obesity pathophysiology. Rev Endocr Metab Disord.

[55] Steiger H, Thaler L, Gauvin L, Joober R, Labbe A, Israel M (2016). Epistatic interactions involving DRD2, DRD4, and COMT polymorphisms and risk of substance abuse in women with binge-purge eating disturbances. J Psychiatr Res.

[56] Calati R, De Ronchi D, Bellini M, Serretti A (2011). The 5-HTTLPR polymorphism and eating disorders: a meta-analysis. Int J Eat Disord.

[57] Steiger H, Richardson J, Schmitz N, Joober R, Israel M, Bruce KR (2009). Association of trait-defined, eating-disorder sub-phenotypes with (biallelic and triallelic) 5HTTLPR variations. J Psychiatr Res.

[58] Micali N, Crous-Bou M, Treasure J, Lawson EA (2017). Association between oxytocin receptor genotype, maternal care, and eating disorder behaviours in a community sample of women. Eur Eat Disord Rev.

[59] Hernández S, Camarena B, González L, Caballero A, Flores G, Aguilar A (2016). A family-based association study of the HTR1B gene in eating disorders. Braz J Psychiatry.

[60] Scott-Van Zeeland AA, Bloss CS, Tewhey R, Bansal V, Torkamani A, Libiger O (2014). Evidence for the role of EPHX2 gene variants in anorexia nervosa. Mol Psychiatry.

[61] Yilmaz Z, Kaplan AS, Tiwari AK, Levitan RD, Piran S, Bergen AW (2014). The role of leptin, melanocortin, and neurotrophin system genes on body weight in anorexia nervosa and bulimia nervosa. J Psychiatr Res.

[62] Martin E, Dourish CT, Rotshtein P, Spetter MS, Higgs S (2019). Interoception and disordered eating: a systematic review. Neurosci Biobehav Rev.

[63] Wade TD, Treloar SA, Heath AC, Martin NG (2009). An examination of the overlap between genetic and environmental risk factors for intentional weight loss and overeating. Int J Eat Disord.

[64] Steiger H, Bruce K, Gauvin L, Groleau P, Joober R, Israel M (2011). Contributions of the glucocorticoid receptor polymorphism (Bcl1) and childhood abuse to risk of bulimia nervosa. Psychiatry Res.

[65] Cellini E, Castellini G, Ricca V, Bagnoli S, Tedde A, Rotella CM (2010). Glucocorticoid receptor gene polymorphisms in Italian patients with eating disorders and obesity. Psychiatr Genet.

[66] Steiger H, Gauvin L, Joober R, Israel M, Badawi G, Groleau P (2012). Interaction of the BcII glucocorticoid receptor polymorphism and childhood abuse in Bulimia Nervosa (BN): relationship to BN and to associated trait manifestations. J Psychiatr Res.

[67] Monteleone AM, Monteleone P, Serino I, Scognamiglio P, Di Genio M, Maj M (2015). Childhood trauma and cortisol awakening response in symptomatic patients with anorexia nervosa and bulimia nervosa. Int J Eat Disord.

[68] Thaler L, Gauvin L, Joober R, Groleau P, de Guzman R, Ambalavanan A (2014). Methylation of BDNF in women with bulimic eating syndromes: associations with childhood abuse and borderline personality disorder. Prog Neuropsychopharmacol Biol Psychiatry.

[69] Rozenblat V, Ong D, Fuller-Tyszkiewicz M, Akkermann K, Collier D, Engels RCME (2017). A systematic review and secondary data analysis of the interactions between the serotonin transporter 5-HTTLPR polymorphism and environmental and psychological factors in eating disorders. J Psychiatr Res.

[70] Micali N, Field AE, Treasure JL, Evans DM (2015). Are obesity risk genes associated with binge eating in adolescence?. Obesity.

[71] Qasim A, Mayhew AJ, Ehtesham S, Alyass A, Volckmar AL, Herpertz S (2019). Gain-of-function variants in the melanocortin 4 receptor gene confer susceptibility to binge eating disorder in subjects with obesity: a systematic review and meta-analysis. Obes Rev.

[72] Bonnefond A, Keller R, Meyre D, Stutzmann F, Thuillier D, Stefanov DG (2016). Eating Behavior, Low-Frequency Functional Mutations in the Melanocortin-4 Receptor (MC4R) Gene, and Outcomes of Bariatric Operations: A 6-Year Prospective Study. Diabetes Care.

[73] Davis C, Levitan RD, Yilmaz Z, Kaplan AS, Carter JC, Kennedy JL (2012). Binge eating disorder and the dopamine D2 receptor: genotypes and sub-phenotypes. Prog Neuropsychopharmacol Biol Psychiatry.

[74] Koren R, Munn-Chernoff MA, Duncan AE, Bucholz KK, Madden PAF, Heath AC (2014). Is the relationship between binge eating episodes and personality attributable to genetic factors?. Twin Res Hum Genet.

[75] Sabbagh U, Mullegama S, Wyckoff GJ (2016). Identification and evolutionary analysis of potential candidate genes in a human eating disorder. Biomed Res Int.

[76] Root TL, Thornton LM, Lindroos AK, Stunkard AJ, Lichtenstein P, Pedersen NL (2010). Shared and unique genetic and environmental influences on binge eating and night eating: a Swedish twin study. Eat Behav.

[77] Lundgren JD, Drapeau V, Allison KC, Gallant AR, Tremblay A, Lambert MA (2012). Prevalence and familial patterns of night eating in the Québec adipose and lifestyle investigation in youth (QUALITY) study. Obesity.

[78] Fetissov SO, Hökfelt T (2019). On the origin of eating disorders: altered signaling between gut microbiota, adaptive immunity and the brain melanocortin system regulating feeding behavior. Curr Opin Pharmacol.

[79] Glenny EM, Bulik-Sullivan EC, Tang Q, Bulik CM, Carroll IM (2017). Eating disorders and the intestinal microbiota: mechanisms of energy homeostasis and behavioral influence. Curr Psychiatry Rep.

[80] Carbone EA, D’Amato P, Vicchio G, De Fazio P, Segura-Garcia C (2020). A systematic review on the role of microbiota in the pathogenesis and treatment of eating disorders. Eur Psychiatry.

[81] Hanachi M, Manichanh C, Schoenenberger A, Pascal V, Levenez F, Cournède N (2019). Altered host-gut microbes symbiosis in severely malnourished anorexia nervosa (AN) patients undergoing enteral nutrition: an explicative factor of functional intestinal disorders?. Clin Nutr.

[82] Schorr M, Miller KK (2017). The endocrine manifestations of anorexia nervosa: mechanisms and management. Nat Rev Endocrinol.

[83] Breton J, Legrand R, Akkermann K, Järv A, Harro J, Déchelotte P (2016). Elevated plasma concentrations of bacterial ClpB protein in patients with eating disorders. Int J Eat Disord.

[84] Eddy KT, Lawson EA, Meade C, Meenaghan E, Horton SE, Misra M (2015). Appetite regulatory hormones in women with anorexia nervosa: binge-eating/purging versus restricting type. J Clin Psychiatry.

[85] Breton J, Déchelotte P, Ribet D (2019). Intestinal microbiota and anorexia nervosa. Clin Nutr Exp.

[86] Zerwas S, Larsen JT, Petersen L, Thornton LM, Quaranta M, Koch SV (2017). Eating disorders, autoimmune, and autoinflammatory disease. Pediatrics.

[87] Raevuori A, Haukka J, Vaarala O, Suvisaari JM, Gissler M, Grainger M (2014). The increased risk for autoimmune diseases in patients with eating disorders. PLoS ONE.

[88] Nikniaz Z, Beheshti S, Abbasalizad Farhangi M, Nikniaz L (2021). A systematic review and meta-analysis of the prevalence and odds of eating disorders in patients with celiac disease and vice-versa. Int J Eat Disord.

[89] Avila JT, Park KT, Golden NH (2019). Eating disorders in adolescents with chronic gastrointestinal and endocrine diseases. Lancet Child Adolesc Health.

[90] Nip ASY, Reboussin BA, Dabelea D, Bellatorre A, Mayer-Davis EJ, Kahkoska AR (2019). Disordered eating behaviors in youth and young adults with type 1 or type 2 diabetes receiving insulin therapy: the SEARCH for diabetes in youth study. Diabetes Care.

[91] Reinehr T, Dieris B, Galler A, Teufel M, Berger G, Stachow R (2019). Worse metabolic control and dynamics of weight status in adolescent girls point to eating disorders in the first years after manifestation of type 1 diabetes mellitus: findings from the diabetes Patienten Verlaufsdokumentation Registry. J Pediatr.

[92] Peterson CM, Fischer S, Young-Hyman D (2015). Topical review: a comprehensive risk model for disordered eating in youth with type 1 diabetes. J Pediatr Psychol.

[93] Conviser JH, Fisher SD, McColley SA (2018). Are children with chronic illnesses requiring dietary therapy at risk for disordered eating or eating disorders? A systematic review. Int J Eat Disord.

[94] d’Emden H, Holden L, McDermott B, Harris M, Gibbons K, Gledhill A (2013). Disturbed eating behaviours and thoughts in Australian adolescents with type 1 diabetes. J Paediatr Child Health.

[95] Scheuing N, Bartus B, Berger G, Haberland H, Icks A, Knauth B (2014). Clinical characteristics and outcome of 467 patients with a clinically recognized eating disorder identified among 52,215 patients with type 1 diabetes: a multicenter german/austrian study. Diabetes Care.

[96] Bächle C, Stahl-Pehe A, Rosenbauer J (2016). Disordered eating and insulin restriction in youths receiving intensified insulin treatment: Results from a nationwide population-based study. Int J Eat Disord.

[97] Dias Santana D, Mitchison D, Gonzalez-Chica D, Touyz S, Stocks N, Appolinario JC (2019). Associations between self-reported diabetes mellitus, disordered eating behaviours, weight/shape overvaluation, and health-related quality of life. J Eat Disord.

[98] St-Hilaire A, Steiger H, Liu A, Laplante DP, Thaler L, Magill T (2015). A prospective study of effects of prenatal maternal stress on later eating-disorder manifestations in affected offspring: preliminary indications based on the Project Ice Storm cohort. Int J Eat Disord.

[99] Lydecker JA, Pisetsky EM, Mitchell KS, Thornton LM, Kendler KS, Reichborn-Kjennerud T (2012). Association between co-twin sex and eating disorders in opposite sex twin pairs: evaluations in North American, Norwegian, and Swedish samples. J Psychosom Res.

[100] Kothari R, Gafton J, Treasure J, Micali N (2014). 2D:4D ratio in children at familial high-risk for eating disorders: The role of prenatal testosterone exposure. Am J Hum Biol.

[101] Micali N, Treasure J (2009) Biological effects of a maternal ED on pregnancy and foetal development: a review. Eur Eat Disord Rev 17(6):448–54. 10.1002/erv.963 19851992

[102] Micali N, Kothari R, Nam KW, Gioroukou E, Walshe M, Allin M (2015). Eating disorder psychopathology, brain structure, neuropsychological correlates and risk mechanisms in very preterm young adults. Eur Eat Disord Rev.

[103] Jones C, Pearce B, Barrera I, Mummert A (2017). Fetal programming and eating disorder risk. J Theor Biol.

[104] Watson HJ, Diemer EW, Zerwas S, Gustavson K, Knudsen GP, Torgersen L (2019). Prenatal and perinatal risk factors for eating disorders in women: a population cohort study. Int J Eat Disord.

[105] Su X, Xu B, Liang H, Olsen J, Yuan W, Cnattingius S (2015). Prenatal maternal bereavement and risk of eating disorders in infants and toddlers: a population-based cohort study. BMC Psychiatry.

[106] Reba-Harrelson L, Von Holle A, Hamer RM, Torgersen L, Reichborn-Kjennerud T, Bulik CM (2010). Patterns of maternal feeding and child eating associated with eating disorders in the Norwegian Mother and Child Cohort Study (MoBa). Eat Behav.

[107] Marzola E, Cavallo F, Panero M, Porliod A, Amodeo L, Abbate-Daga G (2021). The role of prenatal and perinatal factors in eating disorders: a systematic review. Arch Womens Ment Health.

[108] Dörsam AF, Preißl H, Micali N, Lörcher SB, Zipfel S, Giel KE (2019). The impact of maternal eating disorders on dietary intake and eating patterns during pregnancy: a systematic review. Nutrients.

[109] Watson HJ, Von Holle A, Hamer RM, Knoph Berg C, Torgersen L, Magnus P (2013). Remission, continuation and incidence of eating disorders during early pregnancy: a validation study in a population-based birth cohort. Psychol Med.

[110] Knoph Berg C, Torgersen L, Von Holle A, Hamer RM, Bulik CM, Reichborn-Kjennerud T (2011). Factors associated with binge eating disorder in pregnancy. Int J Eat Disord.

[111] Watson HJ, Von Holle A, Knoph C, Hamer RM, Torgersen L, Reichborn-Kjennerud T (2015). Psychosocial factors associated with bulimia nervosa during pregnancy: an internal validation study. Int J Eat Disord.

[112] Canals J, Sancho C, Arija MV (2009). Influence of parent’s eating attitudes on eating disorders in school adolescents. Eur Child Adolesc Psychiatry.

[113] de Barse LM, Tharner A, Micali N, Jaddoe VVW, Hofman A, Verhulst FC (2015). Does maternal history of eating disorders predict mothers’ feeding practices and preschoolers’ emotional eating?. Appetite.

[114] Kothari R, Rosinska M, Treasure J, Micali N (2014). The early cognitive development of children at high risk of developing an eating disorder. Eur Eat Disord Rev.

[115] Larsen JT, Munk-Olsen T, Bulik CM, Thornton LM, Koch SV, Mortensen PB (2017). Early childhood adversities and risk of eating disorders in women: a Danish register-based cohort study. Int J Eat Disord.

[116] Nicholls DE, Viner RM (2009). Childhood risk factors for lifetime anorexia nervosa by age 30 years in a national birth cohort. J Am Acad Child Adolesc Psychiatry.

[117] Rossman SM, Eddy KT, Franko DL, Rose J, DuBois R, Weissman RS (2020). Behavioral symptoms of eating disorders among adopted adolescents and young adults in the United States: Findings from the Add Health survey. Int J Eat Disord.

[118] Allen KL, Byrne SM, Oddy WH, Schmidt U, Crosby RD (2014). Risk factors for binge eating and purging eating disorders: differences based on age of onset. Int J Eat Disord.

[119] Nicholls D, Statham R, Costa S, Micali N, Viner RM (2016). Childhood risk factors for lifetime bulimic or compulsive eating by age 30 years in a British national birth cohort. Appetite.

[120] Ahrén JC, Chiesa F, Af Klinteberg B, Koupil I (2012). Psychosocial determinants and family background in anorexia nervosa–results from the Stockholm Birth Cohort Study. Int J Eat Disord.

[121] Huemer J, Haidvogl M, Mattejat F, Wagner G, Nobis G, Fernandez-Aranda F (2012). Perception of autonomy and connectedness prior to the onset of anorexia nervosa and bulimia nervosa. Z Kinder Jugendpsychiatr Psychother.

[122] Micali N, Martini MG, Thomas JJ, Eddy KT, Kothari R, Russell E (2017). Lifetime and 12-month prevalence of eating disorders amongst women in mid-life: a population-based study of diagnoses and risk factors. BMC Med.

[123] Krug I, King RM, Youssef GJ, Sorabji A, Wertheim EH, Le Grange D (2016). The effect of low parental warmth and low monitoring on disordered eating in mid-adolescence: findings from the Australian Temperament Project. Appetite.

[124] Amianto F, Abbate-Daga G, Morando S, Sobrero C, Fassino S (2011). Personality development characteristics of women with anorexia nervosa, their healthy siblings and healthy controls: What prevents and what relates to psychopathology?. Psychiatry Res.

[125] Watson HJ, O’Brien A, Sadeh-Sharvit S (2018). Children of parents with eating disorders. Curr Psychiatry Rep.

[126] Watkins B, Cooper PJ, Lask B (2012). History of eating disorder in mothers of children with early onset eating disorder or disturbance. Eur Eat Disord Rev.

[127] Micali N, Simonoff E, Stahl D, Treasure J (2011). Maternal eating disorders and infant feeding difficulties: maternal and child mediators in a longitudinal general population study. J Child Psychol Psychiatry.

[128] Haynos AF, Watts AW, Loth KA, Pearson CM, Neumark-Stzainer D (2016). Factors predicting an escalation of restrictive eating during adolescence. J Adolesc Health.

[129] Krug I, Treasure J, Anderluh M, Bellodi L, Cellini E, Collier D (2009). Associations of individual and family eating patterns during childhood and early adolescence: a multicentre European study of associated eating disorder factors. Br J Nutr.

[130] Berge JM, Maclehose R, Loth KA, Eisenberg M, Bucchianeri MM, Neumark-Sztainer D (2013). Parent conversations about healthful eating and weight: associations with adolescent disordered eating behaviors. JAMA Pediatr.

[131] Ellis JM, Schenk RR, Galloway AT, Zickgraf HF, Webb RM, Martz DM (2018). A multidimensional approach to understanding the potential risk factors and covariates of adult picky eating. Appetite.

[132] Gonçalves SF, Machado BC, Martins C (2014). Eating and weight/shape criticism as a specific life-event related to bulimia nervosa: a case control study. J Psychol.

[133] Degortes D, Santonastaso P, Zanetti T, Tenconi E, Veronese A, Favaro A (2014). Stressful life events and binge eating disorder. Eur Eat Disord Rev.

[134] Rodgers RF, Watts AW, Austin SB, Haines J, Neumark-Sztainer D (2017). Disordered eating in ethnic minority adolescents with overweight. Int J Eat Disord.

[135] Berkowitz SA, Witt AA, Gillberg C, Råstam M, Wentz E, Lowe MR (2016). Childhood body mass index in adolescent-onset anorexia nervosa. Int J Eat Disord.

[136] Veses AM, Martínez-Gómez D, Gómez-Martínez S, Zapatera B, Veiga ÓL, Marcos A (2011). Association between excessive body fat and eating-disorder risk in adolescents: the AFINOS Study. Med Clin.

[137] Espinoza P, Penelo E, Raich RM (2010). Disordered eating behaviors and body image in a longitudinal pilot study of adolescent girls: what happens 2 years later?. Body Image.

[138] van Eeden AE, Oldehinkel AJ, van Hoeken D, Hoek HW (2021). Risk factors in preadolescent boys and girls for the development of eating pathology in young adulthood. Int J Eat Disord.

[139] Loth K, Wall M, Larson N, Neumark-Sztainer D (2015). Disordered eating and psychological well-being in overweight and nonoverweight adolescents: secular trends from 1999 to 2010. Int J Eat Disord.

[140] Lebow J, Sim LA, Kransdorf LN (2015). Prevalence of a history of overweight and obesity in adolescents with restrictive eating disorders. J Adolesc Health.

[141] Balantekin KN, Grammer AC, Fitzsimmons-Craft EE, Eichen DE, Graham AK, Monterubio GE (2021). Overweight and obesity are associated with increased eating disorder correlates and general psychopathology in university women with eating disorders. Eat Behav.

[142] Stice E, Desjardins CD (2018). Interactions between risk factors in the prediction of onset of eating disorders: Exploratory hypothesis generating analyses. Behav Res Ther.

[143] Yilmaz Z, Gottfredson NC, Zerwas SC, Bulik CM, Micali N (2019). Developmental premorbid body mass index trajectories of adolescents with eating disorders in a longitudinal population cohort. J Am Acad Child Adolesc Psychiatry.

[144] Allen KL, Byrne SM, Forbes D, Oddy WH (2009). Risk factors for full- and partial-syndrome early adolescent eating disorders: a population-based pregnancy cohort study. J Am Acad Child Adolesc Psychiatry.

[145] Machado BC, Gonçalves SF, Martins C, Brandão I, Roma-Torres A, Hoek HW (2016). Anorexia nervosa versus bulimia nervosa: differences based on retrospective correlates in a case-control study. Eat Weight Disord.

[146] Gonçalves S, Machado BC, Martins C, Hoek HW, Machado PPP (2016). Retrospective correlates for bulimia nervosa: a matched case-control study. Eur Eat Disord Rev.

[147] Allen KL, Byrne SM, Crosby RD, Stice E (2016). Testing for interactive and non-linear effects of risk factors for binge eating and purging eating disorders. Behav Res Ther.

[148] Allen KL, Byrne SM, Crosby RD (2015). Distinguishing between risk factors for bulimia nervosa, binge eating disorder, and purging disorder. J Youth Adolesc.

[149] Haines J, Kleinman KP, Rifas-Shiman SL, Field AE, Austin SB (2010). Examination of shared risk and protective factors for overweight and disordered eating among adolescents. Arch Pediatr Adolesc Med.

[150] Saltzman JA, Liechty JM (2016). Family correlates of childhood binge eating: a systematic review. Eat Behav.

[151] Klein KM, Brown TA, Kennedy GA, Keel PK (2017). Examination of parental dieting and comments as risk factors for increased drive for thinness in men and women at 20-year follow-up. Int J Eat Disord.

[152] Neumark-Sztainer D, Bauer KW, Friend S, Hannan PJ, Story M, Berge JM (2010). Family weight talk and dieting: how much do they matter for body dissatisfaction and disordered eating behaviors in adolescent girls?. J Adolesc Health.

[153] Caslini M, Bartoli F, Crocamo C, Dakanalis A, Clerici M, Carrà G (2016). Disentangling the association between child abuse and eating disorders: a systematic review and meta-analysis. Psychosom Med.

[154] Belli H, Ural C, Akbudak M, Sagaltıcı E (2019). Levels of childhood traumatic experiences and dissociative symptoms in extremely obese patients with and without binge eating disorder. Nord J Psychiatry.

[155] Latzer Y, Rozenstain-Hason M, Kabakov O, Givon M, Mizrachi S, Alon S (2020). Childhood maltreatment in patients with binge eating disorder with and without night eating syndrome vs. control. Psychiatry Res.

[156] Solmi M, Radua J, Stubbs B, Ricca V, Moretti D, Busatta D (2020). Risk factors for eating disorders: an umbrella review of published meta-analyses. Brazil J Psychiatry.

[157] Stice E, Gau JM, Rohde P, Shaw H (2017). Risk factors that predict future onset of each DSM–5 eating disorder: predictive specificity in high-risk adolescent females. J Abnorm Psychol.

[158] Fischer S, Stojek M, Hartzell E (2010). Effects of multiple forms of childhood abuse and adult sexual assault on current eating disorder symptoms. Eat Behav.

[159] Afifi TO, Sareen J, Fortier J, Taillieu T, Turner S, Cheung K (2017). Child maltreatment and eating disorders among men and women in adulthood: results from a nationally representative United States sample. Int J Eat Disord.

[160] Quilliot D, Brunaud L, Mathieu J, Quenot C, Sirveaux MA, Kahn JP (2019). Links between traumatic experiences in childhood or early adulthood and lifetime binge eating disorder. Psychiatry Res.

[161] Hayes S, Linardon J, Kim C, Mitchison D (2021). Understanding the relationship between sexual harassment and eating disorder psychopathology: a systematic review and meta-analysis. Int J Eat Disord.

[162] Monteleone AM, Cascino G, Pellegrino F, Ruzzi V, Patriciello G, Marone L (2019). The association between childhood maltreatment and eating disorder psychopathology: a mixed-model investigation. Eur Psychiatry.

[163] Hazzard VM, Bauer KW, Mukherjee B, Miller AL, Sonneville KR (2019). Associations between childhood maltreatment latent classes and eating disorder symptoms in a nationally representative sample of young adults in the United States. Child Abuse Negl.

[164] Krug I, Fuller-Tyszkiewicz M, Anderluh M, Bellodi L, Bagnoli S, Collier D (2015). A new social-family model for eating disorders: a European multicentre project using a case-control design. Appetite.

[165] Imperatori C, Innamorati M, Lamis DA, Farina B, Pompili M, Contardi A (2016). Childhood trauma in obese and overweight women with food addiction and clinical-level of binge eating. Child Abuse Negl.

[166] Amianto F, Spalatro AV, Rainis M, Andriulli C, Lavagnino L, Abbate-Daga G (2018). Childhood emotional abuse and neglect in obese patients with and without binge eating disorder: personality and psychopathology correlates in adulthood. Psychiatry Res.

[167] Coffino JA, Grilo CM, Udo T (2020). Childhood food neglect and adverse experiences associated with DSM-5 eating disorders in U.S. National Sample. J Psychiatr Res.

[168] Hymowitz G, Salwen J, Salis KL (2017). A mediational model of obesity related disordered eating: the roles of childhood emotional abuse and self-perception. Eat Behav.

[169] Smith CE, Pisetsky EM, Wonderlich SA, Crosby RD, Mitchell JE, Joiner TE (2016). Is childhood trauma associated with lifetime suicide attempts in women with bulimia nervosa?. Eat Weight Disord.

[170] Forrest LN, Grilo CM, Udo T (2021). Suicide attempts among people with eating disorders and adverse childhood experiences: Results from a nationally representative sample of adults. Int J Eat Disord.

[171] Copeland WE, Bulik CM, Zucker N, Wolke D, Lereya ST, Costello EJ (2015). Does childhood bullying predict eating disorder symptoms? A prospective, longitudinal analysis. Int J Eat Disord.

[172] Carretero-García A, Sánchez Planell L, Doval E, Rusiñol Estragués J, Raich Escursell RM, Vanderlinden J (2012). Repeated traumatic experiences in eating disorders and their association with eating symptoms. Eat Weight Disord.

[173] Lie SØ, Bulik CM, Andreassen OA, Rø Ø, Bang L (2021). The association between bullying and eating disorders: a case-control study. Int J Eat Disord.

[174] Rubin AG, Schvey NA, Shank LM, Altman DR, Swanson TN, Ramirez E (2021). Associations between weight-based teasing and disordered eating behaviors among youth. Eat Behav.

[175] Hicks White AA, Pratt KJ, Cottrill C (2018). The relationship between trauma and weight status among adolescents in eating disorder treatment. Appetite.

[176] Marco JH, Tormo-Irun MP, Galán-Escalante A, Gonzalez-García C (2018). Is Cybervictimization associated with body dissatisfaction, depression, and eating disorder psychopathology?. Cyberpsychol Behav Soc Netw.

[177] Degortes D, Zanetti T, Tenconi E, Santonastaso P, Favaro A (2014). Childhood obsessive-compulsive traits in anorexia nervosa patients, their unaffected sisters and healthy controls: a retrospective study. Eur Eat Disord Rev.

[178] Farstad SM, McGeown LM, von Ranson KM (2016). Eating disorders and personality, 2004–2016: a systematic review and meta-analysis. Clin Psychol Rev.

[179] Mousavi F, Rozsa S, Nilsson T, Archer T, Anckarsäter H, Garcia D (2015). Personality and intelligence: persistence, not self-directedness, cooperativeness or self-transcendence, is related to twins’ cognitive abilities. PeerJ.

[180] Joyce F, Watson HJ, Egan SJ, Kane RT (2012). Mediators between perfectionism and eating disorder psychopathology in a community sample. Eat Behav.

[181] Widiger TA, Oltmanns JR (2017). Neuroticism is a fundamental domain of personality with enormous public health implications. World Psychiatry.

[182] Robinson L, Zhang Z, Jia T, Bobou M, Roach A, Campbell I (2020). Association of genetic and phenotypic assessments with onset of disordered eating behaviors and comorbid mental health problems among adolescents. JAMA Netw Open.

[183] Carr MM, Wiedemann AA, Macdonald-Gagnon G, Potenza MN (2021). Impulsivity and compulsivity in binge eating disorder: a systematic review of behavioral studies. Prog Neuropsychopharmacol Biol Psychiatry.

[184] Amianto F, Siccardi S, Abbate-Daga G, Marech L, Barosio M, Fassino S (2012). Does anger mediate between personality and eating symptoms in bulimia nervosa?. Psychiatry Res.

[185] Forsén Mantilla E, Bergsten K, Birgegård A (2014). Self-image and eating disorder symptoms in normal and clinical adolescents. Eat Behav.

[186] Messer M, Anderson C, Linardon J (2021). Self-compassion explains substantially more variance in eating disorder psychopathology and associated impairment than mindfulness. Body Image.

[187] Drieberg H, McEvoy PM, Hoiles KJ, Shu CY, Egan SJ (2019). An examination of direct, indirect and reciprocal relationships between perfectionism, eating disorder symptoms, anxiety, and depression in children and adolescents with eating disorders. Eat Behav.

[188] Holland LA, Bodell LP, Keel PK (2013). Psychological factors predict eating disorder onset and maintenance at 10-year follow-up. Eur Eat Disord Rev.

[189] Boucher K, Côté M, Gagnon-Girouard MP, Ratté C, Bégin C (2018). Eating pathology among patients with anorexia nervosa and bulimia nervosa: the role of narcissism and self-esteem. J Nerv Ment Dis.

[190] Levinson CA, Brosof LC, Ram SS, Pruitt A, Russell S, Lenze EJ (2019). Obsessions are strongly related to eating disorder symptoms in anorexia nervosa and atypical anorexia nervosa. Eat Behav.

[191] Zaidel E, Kaplan J, Cohen H, Stemmer B (2007). The cross-cultural brain. Consciousness and cognition.

[192] Brown TA, Avery JC, Jones MD, Anderson LK, Wierenga CE, Kaye WH (2018). The impact of alexithymia on emotion dysregulation in anorexia nervosa and bulimia nervosa over time. Eur Eat Disord Rev.

[193] Speranza M, Loas G, Guilbaud O, Corcos M (2011). Are treatment options related to alexithymia in eating disorders? Results from a three-year naturalistic longitudinal study. Biomed Pharmacother.

[194] Prefit AB, Cândea DM, Szentagotai-Tătar A (2019). Emotion regulation across eating pathology: a meta-analysis. Appetite.

[195] Waxman SE (2009). A systematic review of impulsivity in eating disorders. Eur Eat Disord Rev.

[196] Lee-Winn AE, Townsend L, Reinblatt SP, Mendelson T (2016). Associations of neuroticism-impulsivity and coping with binge eating in a nationally representative sample of adolescents in the United States. Eat Behav.

[197] Reas DL, Pedersen G, Rø Ø (2016). Impulsivity-related traits distinguish women with co-occurring bulimia nervosa in a psychiatric sample. Int J Eat Disord.

[198] Vansteelandt K, Probst M, Pieters G (2013). Assessing affective variability in eating disorders: affect spins less in anorexia nervosa of the restrictive type. Eat Behav.

[199] Lilienthal KR, Weatherly JN (2013). Understanding the relationships between body esteem, risk for anorexia nervosa, and domain-dependent decision-making impulsivity in a college sample. Body Image.

[200] Howard M, Gregertsen EC, Hindocha C, Serpell L (2020). Impulsivity and compulsivity in anorexia and bulimia nervosa: a systematic review. Psychiatry Res.

[201] Davico C, Amianto F, Gaiotti F, Lasorsa C, Peloso A, Bosia C (2019). Clinical and personality characteristics of adolescents with anorexia nervosa with or without non-suicidal self-injurious behavior. Compr Psychiatry.

[202] Saraçlı Ö, Atasoy N, Akdemir A, Güriz O, Konuk N, Sevinçer GM (2015). The prevalence and clinical features of the night eating syndrome in psychiatric out-patient population. Compr Psychiatry.

[203] Pearson CM, Pisetsky EM, Goldschmidt AB, Lavender JM, Wonderlich SA, Crosby RD (2016). Personality psychopathology differentiates risky behaviors among women with bulimia nervosa. Int J Eat Disord.

[204] Castellini G, Rossi E, Ricca V (2020). The relationship between eating disorder psychopathology and sexuality: etiological factors and implications for treatment. Curr Opin Psychiatry.

[205] Adambegan M, Wagner G, Nader IW, Fernandez-Aranda F, Treasure J, Karwautz A (2012). Internalizing and externalizing behaviour problems in childhood contribute to the development of anorexia and bulimia nervosa-a study comparing sister pairs. Eur Eat Disord Rev.

[206] Pearson CM, Riley EN, Davis HA, Smith GT (2014). Two pathways toward impulsive action: an integrative risk model for bulimic behavior in youth. J Child Psychol Psychiatry.

[207] Stice E (2002). Risk and maintenance factors for eating pathology: a meta-analytic review. Psychol Bull.

[208] Dakanalis A, Timko CA, Carrà G, Clerici M, Zanetti MA, Riva G (2014). Testing the original and the extended dual-pathway model of lack of control over eating in adolescent girls. A two-year longitudinal study. Appetite.

[209] Wolz I, Granero R, Fernández-Aranda F (2017). A comprehensive model of food addiction in patients with binge-eating symptomatology: the essential role of negative urgency. Compr Psychiatry.

[210] Utschig AC, Presnell K, Madeley MC, Smits JAJ (2010). An investigation of the relationship between fear of negative evaluation and bulimic psychopathology. Eat Behav.

[211] Micali N, Hilton K, Nakatani E, Natatani E, Heyman I, Turner C (2011). Is childhood OCD a risk factor for eating disorders later in life? A longitudinal study. Psychol Med.

[212] Navarro-Haro MV, Wessman I, Botella C, García-Palacios A (2015). The role of emotion regulation strategies and dissociation in non-suicidal self-injury for women with borderline personality disorder and comorbid eating disorder. Compr Psychiatry.

[213] Brown M, Hochman A, Micali N (2020). Emotional instability as a trait risk factor for eating disorder behaviors in adolescents: sex differences in a large-scale prospective study. Psychol Med.

[214] Dougherty EN, Murphy J, Hamlett S, George R, Badillo K, Johnson NK (2020). Emotion regulation flexibility and disordered eating. Eat Behav.

[215] von Lojewski A, Fisher A, Abraham S (2013). Have personality disorders been overdiagnosed among eating disorder patients?. Psychopathology.

[216] Friborg O, Martinussen M, Kaiser S, Øvergård KT, Martinsen EW, Schmierer P (2014). Personality disorders in eating disorder not otherwise specified and binge eating disorder: a meta-analysis of comorbidity studies. J Nerv Ment Dis.

[217] Baker JH, Thornton LM, Strober M, Brandt H, Crawford S, Fichter MM (2013). Temporal sequence of comorbid alcohol use disorder and anorexia nervosa. Addict Behav.

[218] Peñas-Lledó E, Bulik CM, Lichtenstein P, Larsson H, Baker JH (2015). Risk for self-reported anorexia or bulimia nervosa based on drive for thinness and negative affect clusters/dimensions during adolescence: a three-year prospective study of the TChAD cohort. Int J Eat Disord.

[219] Levinson CA, Zerwas S, Calebs B, Forbush K, Kordy H, Watson H (2017). The core symptoms of bulimia nervosa, anxiety, and depression: a network analysis. J Abnorm Psychol.

[220] Godart N, Radon L, Curt F, Duclos J, Perdereau F, Lang F (2015). Mood disorders in eating disorder patients: prevalence and chronology of ONSET. J Affect Disord.

[221] Presnell K, Stice E, Seidel A, Madeley MC (2009). Depression and eating pathology: prospective reciprocal relations in adolescents. Clin Psychol Psychother.

[222] Skinner HH, Haines J, Austin SB, Field AE (2012). A prospective study of overeating, binge eating, and depressive symptoms among adolescent and young adult women. J Adolesc Health.

[223] Rojo-Moreno L, Arribas P, Plumed J, Gimeno N, García-Blanco A, Vaz-Leal F (2015). Prevalence and comorbidity of eating disorders among a community sample of adolescents: 2-year follow-up. Psychiatry Res.

[224] Treasure J, Zipfel S, Micali N, Wade T, Stice E, Claudino A (2015). Anorexia nervosa. Nat Rev Dis Primers.

[225] Swinbourne J, Hunt C, Abbott M, Russell J, St Clare T, Touyz S (2012). The comorbidity between eating disorders and anxiety disorders: prevalence in an eating disorder sample and anxiety disorder sample. Aust N Z J Psychiatry.

[226] Mayer B, Muris P, Meesters C, Zimmermann-van BR (2009). Brief report: direct and indirect relations of risk factors with eating behavior problems in late adolescent females. J Adolesc.

[227] Lloyd EC, Haase AM, Foster CE, Verplanken B (2019). A systematic review of studies probing longitudinal associations between anxiety and anorexia nervosa. Psychiatry Res.

[228] Dellava JE, Thornton LM, Hamer RM, Strober M, Plotnicov K, Klump KL (2010). Childhood anxiety associated with low BMI in women with anorexia nervosa. Behav Res Ther.

[229] Thornton LM, Dellava JE, Root TL, Lichtenstein P, Bulik CM (2011). Anorexia nervosa and generalized anxiety disorder: further explorations of the relation between anxiety and body mass index. J Anxiety Disord.

[230] Sihvola E, Keski-Rahkonen A, Dick DM, Hoek HW, Raevuori A, Rose RJ (2009). Prospective associations of early-onset Axis I disorders with developing eating disorders. Compr Psychiatry.

[231] Ciarma JL, Mathew JM (2017). Social anxiety and disordered eating: the influence of stress reactivity and self-esteem. Eat Behav.

[232] Puccio F, Fuller-Tyszkiewicz M, Youssef G, Mitchell S, Byrne M, Allen N (2017). Longitudinal bi-directional effects of disordered eating, depression and anxiety. Eur Eat Disord Rev.

[233] Buckner JD, Silgado J, Lewinsohn PM (2010). Delineation of differential temporal relations between specific eating and anxiety disorders. J Psychiatr Res.

[234] Levinson CA, Byrne M, Rodebaugh TL (2016). Shame and guilt as shared vulnerability factors: shame, but not guilt, prospectively predicts both social anxiety and bulimic symptoms. Eat Behav.

[235] Mehl A, Rohde P, Gau JM, Stice E (2019). Disaggregating the predictive effects of impaired psychosocial functioning on future DSM-5 eating disorder onset in high-risk female adolescents. Int J Eat Disord.

[236] Grilo CM, White MA, Masheb RM (2009). DSM-IV psychiatric disorder comorbidity and its correlates in binge eating disorder. Int J Eat Disord.

[237] Lundgren JD, Rempfer MV, Brown CE, Goetz J, Hamera E (2010). The prevalence of night eating syndrome and binge eating disorder among overweight and obese individuals with serious mental illness. Psychiatry Res.

[238] Orhan FO, Ozer UG, Ozer A, Altunoren O, Celik M, Karaaslan MF (2011). Night eating syndrome among patients with depression. Isr J Psychiatry Relat Sci.

[239] Melo MCA, de Oliveira RM, de Araújo CFC, de Mesquita LMF, de Bruin PFC, de Bruin VMS (2018). Night eating in bipolar disorder. Sleep Med.

[240] Lunde AV, Fasmer OB, Akiskal KK, Akiskal HS, Oedegaard KJ (2009). The relationship of bulimia and anorexia nervosa with bipolar disorder and its temperamental foundations. J Affect Disord.

[241] Thiebaut S, Godart N, Radon L, Courtet P, Guillaume S (2019). Crossed prevalence results between subtypes of eating disorder and bipolar disorder: a systematic review of the literature. Encephale.

[242] McElroy SL, Crow S, Blom TJ, Biernacka JM, Winham SJ, Geske J (2016). Prevalence and correlates of DSM-5 eating disorders in patients with bipolar disorder. J Affect Disord.

[243] Fornaro M, Perugi G, Gabrielli F, Prestia D, Mattei C, Vinciguerra V (2010). Lifetime co-morbidity with different subtypes of eating disorders in 148 females with bipolar disorders. J Affect Disord.

[244] Thiebaut S, Jaussent I, Maimoun L, Beziat S, Seneque M, Hamroun D (2019). Impact of bipolar disorder on eating disorders severity in real-life settings. J Affect Disord.

[245] McDonald CE, Rossell SL, Phillipou A (2019). The comorbidity of eating disorders in bipolar disorder and associated clinical correlates characterised by emotion dysregulation and impulsivity: a systematic review. J Affect Disord.

[246] Nazar BP, Bernardes C, Peachey G, Sergeant J, Mattos P, Treasure J (2016). The risk of eating disorders comorbid with attention-deficit/hyperactivity disorder: A systematic review and meta-analysis. Int J Eat Disord.

[247] Jahrami H, AlAnsari AM, Janahi AI, Janahi AK, Darraj LR, Faris MAIE (2021). The risk of eating disorders among children and adolescents with attention deficit hyperactivity disorder: Results of a matched cohort study. Int J Pediatr Adolesc Med.

[248] Kaisari P, Dourish CT, Higgs S (2017). Attention deficit hyperactivity disorder (ADHD) and disordered eating behaviour: a systematic review and a framework for future research. Clin Psychol Rev.

[249] Zhang Z, Robinson L, Jia T, Quinlan EB, Tay N, Chu C, et al. Development of Disordered Eating Behaviors and Comorbid Depressive Symptoms in Adolescence: Neural and Psychopathological Predictors. Biological Psychiatry [Internet]. 2020; Available from: https://www.sciencedirect.com/science/article/pii/S0006322320316723 10.1016/j.biopsych.2020.06.003 32778392

[250] Huke V, Turk J, Saeidi S, Kent A, Morgan John F (2013). Autism spectrum disorders in eating disorder populations: a systematic review. Eur Eat Disord Rev.

[251] Dell’Osso L, Carpita B, Gesi C, Cremone IM, Corsi M, Massimetti E (2018). Subthreshold autism spectrum disorder in patients with eating disorders. Compr Psychiatry.

[252] Schaumberg K, Zerwas SC, Bulik CM, Fiorentini C, Micali N (2020). Prospective associations between childhood social communication processes and adolescent eating disorder symptoms in an epidemiological sample. Eur Child Adolesc Psychiatry.

[253] Dinkler L, Taylor MJ, Råstam M, Hadjikhani N, Bulik CM, Lichtenstein P (2021). Anorexia nervosa and autism: a prospective twin cohort study. J Child Psychol Psychiatry.

[254] Grilo CM, White MA, Barnes RD, Masheb RM (2012). Posttraumatic stress disorder in women with binge eating disorder in primary care. J Psychiatr Pract.

[255] Isomaa R, Backholm K, Birgegård A (2015). Posttraumatic stress disorder in eating disorder patients: the roles of psychological distress and timing of trauma. Psychiatry Res.

[256] Brewerton TD, Perlman MM, Gavidia I, Suro G, Genet J, Bunnell DW (2020). The association of traumatic events and posttraumatic stress disorder with greater eating disorder and comorbid symptom severity in residential eating disorder treatment centers. J Eat Disord.

[257] Reyes-Rodríguez ML, Von Holle A, Ulman TF, Thornton LM, Klump KL, Brandt H (2011). Post traumatic stress disorder in anorexia nervosa. Psychosom Med.

[258] Forman-Hoffman VL, Mengeling M, Booth BM, Torner J, Sadler AG (2012). Eating disorders, post-traumatic stress, and sexual trauma in women veterans. Mil Med.

[259] Bartlett BA, Mitchell KS (2015). Eating disorders in military and veteran men and women: a systematic review. Int J Eat Disord.

[260] Mitchell KS, Rasmusson A, Bartlett B, Gerber MR (2014). Eating disorders and associated mental health comorbidities in female veterans. Psychiatry Res.

[261] Crompvoets S (2011). The health and wellbeing of female veterans: a review of the literature. J Milit Vet Health.

[262] Sepulveda A, Carrobles JA, Gandarillas AM (2010). Associated factors of unhealthy eating patterns among Spanish university students by gender. Span J Psychol.

[263] Dakanalis A, Clerici M, Bartoli F, Caslini M, Crocamo C, Riva G (2017). Risk and maintenance factors for young women’s DSM-5 eating disorders. Arch Womens Ment Health.

[264] Favaro A, Caregaro L, Tenconi E, Bosello R, Santonastaso P (2009). Time trends in age at onset of anorexia nervosa and bulimia nervosa. J Clin Psychiatry.

[265] Arduini T, Iorio D, Patacchini E (2019). Weight, reference points, and the onset of eating disorders. J Health Econ.

[266] Klump KL (2013). Puberty as a critical risk period for eating disorders: a review of human and animal studies. Horm Behav.

[267] McNicholas F, Dooley B, McNamara N, Lennon R (2012). The impact of self-reported pubertal status and pubertal timing on disordered eating in Irish adolescents. Eur Eat Disord Rev.

[268] Sidor A, Baba CO, Marton-Vasarhelyi E, Chereches RM (2015). Gender differences in the magnitude of the associations between eating disorders symptoms and depression and anxiety symptoms. Results from a community sample of adolescents. J Ment Health..

[269] Akgül S, Akdemir D, Kara M, Derman O, Çetin FÇ, Kanbur N (2016). The understanding of risk factors for eating disorders in male adolescents. Int J Adolesc Med Health.

[270] Davison KM, Marshall-Fabien GL, Gondara L (2014). Sex differences and eating disorder risk among psychiatric conditions, compulsive behaviors and substance use in a screened Canadian national sample. Gen Hosp Psychiatry.

[271] Evans EH, Adamson AJ, Basterfield L, Le Couteur A, Reilly JK, Reilly JJ (2017). Risk factors for eating disorder symptoms at 12 years of age: A 6-year longitudinal cohort study. Appetite.

[272] Dakanalis A, Zanetti AM, Riva G, Colmegna F, Volpato C, Madeddu F (2015). Male body dissatisfaction and eating disorder symptomatology: moderating variables among men. J Health Psychol.

[273] Blashill AJ (2011). Gender roles, eating pathology, and body dissatisfaction in men: a meta-analysis. Body Image.

[274] Calzo JP, Blashill AJ, Brown TA, Argenal RL (2017). Eating disorders and disordered weight and shape control behaviors in sexual minority populations. Curr Psychiatry Rep.

[275] Gigi I, Bachner-Melman R, Lev-Ari L (2016). The association between sexual orientation, susceptibility to social messages and disordered eating in men. Appetite.

[276] Gonzales M, Blashill AJ (2021). Ethnic/racial and gender differences in body image disorders among a diverse sample of sexual minority U.S. adults. Body Image.

[277] Uniacke B, Glasofer D, Devlin M, Bockting W, Attia E (2021). Predictors of eating-related psychopathology in transgender and gender nonbinary individuals. Eat Behav.

[278] Mulders-Jones B, Mitchison D, Girosi F, Hay P (2017). Socioeconomic correlates of eating disorder symptoms in an Australian population-based sample. PLoS ONE.

[279] Goodman A, Heshmati A, Koupil I (2014). Family history of education predicts eating disorders across multiple generations among 2 million Swedish males and females. PLoS ONE.

[280] Barry MR, Sonneville KR, Leung CW (2021). Students with food insecurity are more likely to screen positive for an eating disorder at a large, public university in the Midwest. J Acad Nutr Diet.

[281] West CE, Goldschmidt AB, Mason SM, Neumark-Sztainer D (2019). Differences in risk factors for binge eating by socioeconomic status in a community-based sample of adolescents: findings from Project EAT. Int J Eat Disord.

[282] Lydecker JA, Grilo CM (2019). Food insecurity and bulimia nervosa in the United States. Int J Eat Disord.

[283] Bould H, De Stavola B, Magnusson C, Micali N, Dal H, Evans J (2016). The influence of school on whether girls develop eating disorders. Int J Epidemiol.

[284] Ahrén JC, Chiesa F, Koupil I, Magnusson C, Dalman C, Goodman A (2013). We are family–parents, siblings, and eating disorders in a prospective total-population study of 250,000 Swedish males and females. Int J Eat Disord.

[285] Cheng ZH, Perko VL, Fuller-Marashi L, Gau JM, Stice E (2019). Ethnic differences in eating disorder prevalence, risk factors, and predictive effects of risk factors among young women. Eat Behav.

[286] Shagar PS, Donovan CL, Loxton N, Boddy J, Harris N (2019). Is thin in everywhere?: A cross-cultural comparison of a subsection of Tripartite Influence Model in Australia and Malaysia. Appetite.

[287] O’Brien KM, Whelan DR, Sandler DP, Hall JE, Weinberg CR (2017). Predictors and long-term health outcomes of eating disorders. PLoS ONE.

[288] Gorrell S, Grange DL, Blalock DV, Mehler PS, Johnson C, Manwaring J (2021). Gender identity, race/ethnicity and eating pathology in a treatment-seeking community sample. J Behav Cogn Ther.

[289] Hazzard VM, Hahn SL, Bauer KW, Sonneville KR (2019). Binge eating-related concerns and depressive symptoms in young adulthood: seven-year longitudinal associations and differences by race/ethnicity. Eat Behav.

[290] Kwan MY, Gordon KH, Minnich AM (2018). An examination of the relationships between acculturative stress, perceived discrimination, and eating disorder symptoms among ethnic minority college students. Eat Behav.

[291] Forrest LN, Jones PJ, Ortiz SN, Smith AR (2018). Core psychopathology in anorexia nervosa and bulimia nervosa: a network analysis. Int J Eat Disord.

[292] Phillipou A, Castle DJ, Rossell SL (2019). Direct comparisons of anorexia nervosa and body dysmorphic disorder: a systematic review. Psychiatry Res.

[293] Stice E, Gau JM, Rohde P, Shaw H (2017). Risk factors that predict future onset of each DSM-5 eating disorder: Predictive specificity in high-risk adolescent females. J Abnorm Psychol.

[294] Stice E, Van Ryzin MJ (2019). A prospective test of the temporal sequencing of risk factor emergence in the dual pathway model of eating disorders. J Abnorm Psychol.

[295] Prnjak K, Hay P, Mond J, Bussey K, Trompeter N, Lonergan A (2021). The distinct role of body image aspects in predicting eating disorder onset in adolescents after one year. J Abnorm Psychol.

[296] Bristow C, Meurer C, Simmonds J, Snell T (2020). Anti-obesity public health messages and risk factors for disordered eating: a systematic review. Health Promot Int.

[297] Goldschmidt AB, Wall MM, Zhang J, Loth KA, Neumark-Sztainer D (2016). Overeating and binge eating in emerging adulthood: 10-year stability and risk factors. Dev Psychol.

[298] Loth KA, MacLehose R, Bucchianeri M, Crow S, Neumark-Sztainer D (2014). Predictors of dieting and disordered eating behaviors from adolescence to young adulthood. J Adolesc Health.

[299] Sharpe H, Griffiths S, Choo TH, Eisenberg ME, Mitchison D, Wall M (2018). The relative importance of dissatisfaction, overvaluation and preoccupation with weight and shape for predicting onset of disordered eating behaviors and depressive symptoms over 15 years. Int J Eat Disord.

[300] Neumark-Sztainer D, Wall M, Story M, Sherwood NE (2009). Five-year longitudinal predictive factors for disordered eating in a population-based sample of overweight adolescents: implications for prevention and treatment. Int J Eat Disord.

[301] Glashouwer KA, van der Veer RML, Adipatria F, de Jong PJ, Vocks S (2019). The role of body image disturbance in the onset, maintenance, and relapse of anorexia nervosa: a systematic review. Clin Psychol Rev.

[302] Hausenblas HA, Campbell A, Menzel JE, Doughty J, Levine M, Thompson JK (2013). Media effects of experimental presentation of the ideal physique on eating disorder symptoms: a meta-analysis of laboratory studies. Clin Psychol Rev.

[303] Bachner-Melman R, Zohar AH, Elizur Y, Kremer I, Golan M, Ebstein R (2009). Protective self-presentation style: association with disordered eating and anorexia nervosa mediated by sociocultural attitudes towards appearance. Eat Weight Disord.

[304] Mabe AG, Forney KJ, Keel PK (2014). Do you “like” my photo? Facebook use maintains eating disorder risk. Int J Eat Disord.

[305] Griffiths S, Castle D, Cunningham M, Murray SB, Bastian B, Barlow FK (2018). How does exposure to thinspiration and fitspiration relate to symptom severity among individuals with eating disorders? Evaluation of a proposed model. Body Image.

[306] Rodgers RF, Melioli T (2016). The relationship between body image concerns, eating disorders and internet use, Part I: a review of empirical support. Adolescent Res Rev.

[307] Hinojo-Lucena FJ, Aznar-Díaz I, Cáceres-Reche MP, Trujillo-Torres JM, Romero-Rodríguez JM (2019). Problematic internet use as a predictor of eating disorders in students: a systematic review and meta-analysis study. Nutrients.

[308] Jett S, LaPorte DJ, Wanchisn J (2010). Impact of exposure to pro-eating disorder websites on eating behaviour in college women. Eur Eat Disord Rev.

[309] Wooldridge T, Mok C, Chiu S (2014). Content analysis of male participation in pro-eating disorder web sites. Eat Disord.

[310] Sundgot-Borgen J, Torstveit MK (2010). Aspects of disordered eating continuum in elite high-intensity sports. Scand J Med Sci Sports.

[311] Martinsen M, Sundgot-Borgen J (2013). Higher prevalence of eating disorders among adolescent elite athletes than controls. Med Sci Sports Exerc.

[312] Wheatley S, Khan S, Székely AD, Naughton DP, Petróczi A (2012). Expanding the female athlete triad concept to address a public health issue. Perform Enhanc Health.

[313] Brook EM, Tenforde AS, Broad EM, Matzkin EG, Yang HY, Collins JE (2019). Low energy availability, menstrual dysfunction, and impaired bone health: a survey of elite para athletes. Scand J Med Sci Sports.

[314] Gulliver A, Griffiths KM, Mackinnon A, Batterham PJ, Stanimirovic R (2015). The mental health of Australian elite athletes. J Sci Med Sport.

[315] Thompson A, Petrie T, Anderson C (2017). Eating disorders and weight control behaviors change over a collegiate sport season. J Sci Med Sport.

[316] Tan JOA, Calitri R, Bloodworth A, McNamee MJ (2016). Understanding eating disorders in elite gymnastics: ethical and conceptual challenges. Clin Sports Med.

[317] Francisco R, Narciso I, Alarcão M (2013). Individual and relational risk factors for the development of eating disorders in adolescent aesthetic athletes and general adolescents. Eat Weight Disord.

[318] Chapman J, Woodman T (2016). Disordered eating in male athletes: a meta-analysis. J Sports Sci.

[319] Kostrzewa E, Eijkemans MJC, Kas MJ (2013). The expression of excessive exercise co-segregates with the risk of developing an eating disorder in women. Psychiatry Res.

[320] Neumark-Sztainer D, Eisenberg ME, Wall M, Loth KA (2011). Yoga and Pilates: associations with body image and disordered-eating behaviors in a population-based sample of young adults. Int J Eat Disord.

[321] Arcelus J, Witcomb GL, Mitchell A (2014). Prevalence of eating disorders amongst dancers: a systemic review and meta-analysis. Eur Eat Disord Rev.

[322] Taylor CB, Altman T (1997). Priorities in prevention research for eating disorders. Psychopharmacol Bull.

[323] Jones M, Luce KH, Osborne MI, Taylor K, Cunning D, Doyle AC (2008). Randomized, controlled trial of an internet-facilitated intervention for reducing binge eating and overweight in adolescents. Pediatrics.

[324] Becker CB, Stice E (2017). From efficacy to effectiveness to broad implementation: evolution of the body project. J Consult Clin Psychol.

[325] Mitchison D, Basten C, Griffiths S, Murray SB (2017). Beneath the tip of the iceberg: Why so many people with eating disorders are not referred for treatment. Aust Family Physician.

[326] Linville D, Brown T, O’Neil M (2012). Medical providers’ self perceived knowledge and skills for working with eating disorders: a national survey. Eat Disord.

[327] Bryant E, Spielman K, Le A, Marks P, Touyz S, Maguire S (2022). Screening, assessment and diagnosis in the eating disorders: findings from a rapid review. J Eat Disord.

[328] Nagata JM, Ganson KT, Austin SB (2020). Emerging trends in eating disorders among sexual and gender minorities. Curr Opin Psychiatry.

[329] Parker LL, Harriger JA (2020). Eating disorders and disordered eating behaviors in the LGBT population: a review of the literature. J Eat Disord.

[330] Cooper Z, Fairburn CG (2011). The evolution of “enhanced” cognitive behavior therapy for eating disorders: learning from treatment nonresponse. Cogn Behav Pract.

[331] Fairburn CG, Bailey-Straebler S, Basden S, Doll HA, Jones R, Murphy R (2015). A transdiagnostic comparison of enhanced cognitive behaviour therapy (CBT-E) and interpersonal psychotherapy in the treatment of eating disorders. Behav Res Ther.

[332] American Psychiatric Association. Diagnostic and Statistical Manual of Mental Disorders. Vol. 5. 2013.

[333] McKay MT, Cannon M, Chambers D, Conroy RM, Coughlan H, Dodd P (2021). Childhood trauma and adult mental disorder: a systematic review and meta-analysis of longitudinal cohort studies. Acta Psychiatr Scand.

