# Supplementary material for: Risk factors for eating disorders: findings from a rapid review
Source: J Eat Disord. 2023 Jan 17;11:8. doi: 10.1186/s40337-022-00717-4 (PMC9847054; doi:10.1186/s40337-022-00717-4)
Supplement: Supplementary file 1 — Additional file 1. PRISMA Diagram & Included Studies Table. [file 40337_2022_717_MOESM1_ESM.docx]

PRISMA Diagram

articles related to current Rapid Review topic

(n=284)

articles included in overall Rapid Review

(n=1,324)

Expert authors & other sources *related to the current topic*

(n=4)

Articles identified through database searching

(n=17,757)

Articles identified through links and reference lists

(n=36)

Articles after duplicates removed

(n=9,260)

Articles screened through assessment of abstract/title

(n=9,260)

Excluded

(n=7,292)

Full text articles assessed for eligibility

(n=1,968)

Excluded

(n=660)

Expert research collaborative requested articles

(n=12)

Articles included in original Rapid Review

(n=1,320)

Identification

Screening

Eligibility

Included

Included Studies Table

**Table 1.** Studies included in the Rapid Review

| **Author, Year** | **Country** | ***N*** | **Population** | **Aim** | **Design** | **Outcome Measure** |
| --- | --- | --- | --- | --- | --- | --- |
| **Genetics** | | | | | | |
| Baker et al., 2009 | Sweden | 1200 | Community (Adolescents, All Genders) | To examine sex differences in the genetic and environmental risk factors for aspects of body dissatisfaction, drive for thinness, and bulimia. | Cross-sectional (Correlational); | Genetic and environmental contribution |
| Bonnefond et al., 2016 | Switzerland | 872 | Community (Adult, All Genders) | To assess long-term outcomes of bariatric operations in patients characterised for eating behaviour and rare mutations in the melanocortin-4 receptor (MC4R) gene, which is strongly implicated in energy balance | Longitudinal (>5yr) | Eating Behaviour, Low-Frequency Functional Mutations in the Melanocortin-4 Receptor (MC4R) Gene, and Outcomes of Bariatric Operations |
| Bould et al., 2015 | Sweden | 158697 | Community (Children, All Genders) | To investigate whether parental eating disorders (ED) predict ED in children, using a large multigeneration register-based sample. | Cross-Sectional | Parent ED |
| Breithaupt, Hubel & Bulik, 2018 | Worldwide | N/A | Community (Adult, All Genders) | To review recent advances in psychiatric genetics focusing on genome-wide association studies (GWAS) in eating disorders | Review (Systematic) | Personalised intervention |
| Bulik et al., 2010 | Sweden | 43000 | Community (Adult, All Genders) | To determine the extent to which shared genetic and environmental factors contribute to liability to anorexia nervosa and bulimia nervosa. | Cross-sectional (Correlational); | Heritability AN and BN |
| Calati et al., 2011 | Worldwide | 15 studies | N/A | (1) To investigate the association between 5-HTTLPR and eating disorders considered as a whole, including anorexia (AN), bulimia (BN), and binge eating disorder (BED); (2) to extend recently reported findings on the association between 5-HTTLPR and AN-B | Meta-Analysis | 5-HTTLPR Polymorphism |
| Capusan et al., 2017 | Sweden | 18000 | Community (Adult, All Genders) | To investigate these associations in an adult twin population, and to determine the extent to which ADHD symptoms and binge-eating behaviour share genetic and environmental factors. | Cross-sectional (Correlational); | Genetic Risk Factor |
| Cellini et al., 2010 | Italy | 572 | Community (Adult, All Genders) | To assess the role of glucocorticoid receptor gene polymorphisms in the pathophysiology of eating disorders and obesity. | Review (Systematic) | Receptors, ED's and obesity |
| Davis et al., 2012 | USA | 230 | Community (Adult, All Genders) | To investigate the D2 receptors genes (DRD2/ANKK1) and their relation to the BED phenotype and four sub-phenotypes of BED that reflect an enhanced response to positive food stimuli | Cross-sectional (Correlational) | Dopamine D2 receptor |
| Dellava, Kendler, & Neale, 2011 | USA | 2083 | Community (Adult, Women) | To determine the magnitude of shared genetic and environmental components between anorexia nervosa and generalised anxiety disorder. | Review (Systematic) | Genetic variation |
| Gratacòs et al., 2010 | France, Germany, Italy & Spain | 991 | Community (Adult, All Genders) | To explore whether neurotrophic factors affect the natural history of an ED, such as age of illness onset. | Review (Systematic) | Sub phenotypes |
| Hernández et al., 2016 | Mexico | 245 | Community (Adult, All Genders) | To explore the associations in three polymorphisms of a serotonin receptor gene (HTR1B) in the aetiology and clinical features of eating disorders. | Cross-sectional (Correlational); | HTR1B Gene |
| Hübel et al., 2021 | UK | 17,690 | Outpatient (Adult, All Genders) | To identify traits that are genetically associated with binge-type eating disorders | Cross-Sectional | Polygenic scores |
| Kaye et al., 2013 | N/A | N/A | N/A | To assess the evidence related to neurobiology of AN with a focus upon the neural circuitry of gustatory sensory response, interoception, reward, and executive control. | Review (Systematic) | Neurobiology of AN |
| Kirk et al., 2017 | Australia, New Zealand | 3885 | Community (Adult, All Genders) | To describe the features and treatment experiences of a large community sample of people with AN in Australia and New Zealand, collected as part of the Australasian Anorexia Nervosa Genetics Initiative. | Review (Systematic) | Genetic underpinnings |
| Koren et al., 2014 | USA | 3446 | Community (Adult, Women) | To examine the association between personality traits (neuroticism, extraversion, conscientiousness, agreeableness, openness to experience, and control/impulsivity) and binge eating episode, the most widely studied aspect of disordered eating and now the basis for the new DSM-5 binge eating disorder | Cross-sectional (Correlational) | Genetic factors and Binge-eating episodes |
| Lundgren et al., 2010 | USA | 68 | Outpatient (Adult, All Genders) | To assess the prevalence of both night eating syndrome (NES) and binge eating disorder (BED), as well as their co-morbidity, in a sample of obese, weight-loss-seeking individuals with serious mental illness (SMI) | Cross-Sectional | Serious mental illness |
| Lundgren et al., 2012 | Canada | 395 | Mixed (Mixed cohorts, All genders) | To expand the literature on prevalence and familial resemblance of night eating behaviour in the following way | Cross-Sectional | Heritability of NES |
| Martin et al., 2019 | Worldwide | 32883 | Community (Adult, All Genders) | To synthesize evidence from studies that used a range of methods to assess interception related to visceral signals and to signals related to pain, itch, temperature and sensual touch | Review (Systematic) | Interception and disordered eating |
| Mazzeo et al., 2009 | Norway | 1722 | Community (Adult, Women) | To examine genetic and environmental contributions to liability to anorexia nervosa (AN) symptoms in a population-based twin sample using a genetic common pathway model. | Cross-sectional (Correlational); | Genetic and environmental contribution |
| Micali et al., 2015 | UK | 10,906 | Community (Adolescents, All Genders) | To investigate whether genetic variants previously identified as associated with BMI are also associated with adolescent binge eating | Cross-sectional (Correlational) | Obesity Risk Genes |
| Micali et al., 2017 | UK | 3698 | Community (Adult, Women) | To investigate associations between oxytocin receptor gene (OXT-R) polymorphisms, their interaction with maternal care, and eating disorder behaviours in a community sample. | Review (Systematic) | Genotype, Maternal Care and ED behaviours |
| Nicoletti et al., 2019 | N/A | N/A | N/A | To review evidence related to genetic polymorphisms related to hypothalamic appetite and satiety pathways and their role in the development of eating disorders associated with obesity (e.g., binge eating disorder and bulimia nervosa). | Review (Narrative) | Eating disorder polymorphisms in obesity |
| Qasim et al., 2018 | Europe (Switzerland, France, Germany) | 1554 GOF Carriers & 1520 LOF carrier | Community (Adult, All Genders) | To systematically appraise the association between genetic variants and mutations in the coding region of the MC4R and to see whether the functional consequences of the genetic variation on receptor function (i.e. whether the genetic variation leads to a loss or gain of receptor function) modify this association | Systematic Review/ Meta-Analysis (combined) | Gain-of-function variants in the melanocortin 4 receptor gene |
| Root et al., 2010 | Sweden | 11,604 | Community (Adult, All Genders) | To explore the extent to which genetic and environmental factors influencing BE and NE overlap | Cross-Sectional | Genetic and environmental contribution to BED and NES |
| Rozenblat et al., 2017 | Worldwide | 2176 | Community (Adult, All Genders) | To provide a systematic, detailed overview and re-analysis of current GxE studies investigating 5-HTTLPR in eating pathology | Review (Systematic) | Serotonin gene transporter |
| Sabbagh, Mullegama, & Wyckoff, 2016 | USA | N/A | N/A | To find genes linked with eating disorders and associated with both metabolic and neural system | Meta-Analysis | Genes underlying Night Eating Syndrome |
| Steiger et al., 2009 | Canada | 278 | Community (Adult, Women) | To develop an empirical classification of eating-disordered individuals based on assessment of variations along theoretically indicated, comorbid psychopathological traits; and to examine associations between empirically derived sub-phenotypes and 5HTTLPR variants | Cross-sectional (Correlational) | Trait-defined, eating-disorder sub-phenotypes with (biallelic and triallelic) 5HTTLPR variations |
| Steiger et al., 2011 | Canada | 227 | Community (Adult, Women) | To evaluate the hypothesis that traumatic stress can increase risk of bulimia nervosa in individuals who are genetically disposed to lower modulation of physiological stress reactions. | Review (Systematic) | Polymorphism, Childhood abuse and BN |
| Steiger et al., 2012 | Canada | 304 | Community (Adult, Women) | To examine the extent to which the gene-environment interaction between the glucocorticoid receptor polymorphism (BcII) and childhood abuse in individuals with bulimia nervosa is attributable to behavioural impulsivity, sensation seeking, affective instability, or depression. | Review (Systematic) | Polymorphism; Abuse and ED's |
| Thaler et al., 2014 | Canada | 96 | Community (Adult, Women) | To explore the possibility that women with BN, when compared to normal-eater women, might display hypermethylation of the BDNF gene | Cross-sectional (Correlational) | Methylation of BDNF in women |
| Thornton, Mazzeo, & Bulik, 2011 | Worldwide | N/A | Community (Adult, All Genders) | To review biometrical genetic methods and current findings from family and twin studies that investigate the role of genes and environment in the aetiology of eating disorders | Review (Systematic) | Genes and environment - Aetiology |
| Versini et al., 2010 | France | 41 families; 693 young women | Community (Adult, Women) | To identify an association between AN and estrogen receptors, encoded by ESR1 and ESR2 genes. | Cross-sectional (Correlational); | ESR1 and ESR2 genes, |
| Wade et al., 2009 | Australia | 3848 | Community (Adult, Women) | To examine shared genetic and environmental factors between eating that involved intentional weight loss and overeating in a large adult female twin sample. | Cross-sectional (Correlational); | Genetic and environmental variance |
| Wade et al., 2015 | Australia | 1002 | Community (Adult, Women) | To examine the hypothesis that suicidality and eating disorders share genetic risk contributing to the expression of both phenotypes. | Longitudinal (>10yr) | Prevalence and long-term course |
| Watson et al., 2019 | Worldwide | 89,509 | Community (Adult, All Genders) | To identify significant loci associated with AN via a genome-wide association study. | Systematic Review/ Meta-Analysis (combined) | Risk loci and meto-psychiatric |
| Yao et al., 2019 | Sweden | 3,550,118 | Community (Adult, All Genders) | To investigate the genetic association between ADHD and various EDs, including anorexia nervosa (AN) and other EDs such as bulimia nervosa. | Quasi-experimental (intervention) | General Genetic Factor |
| Yilmaz et al., 2014 | Worldwide | 1311 | Community (Adult, All Genders) | To assess the role of genes involved in appetite and weight regulation in conferring risk for AN and BN. | Review (Systematic) | Role of Genes in AN and BN |
| Yilmaz et al., 2017 | Worldwide | 1983 | Community (Adult, Women) | To assess the prevalence of large, rare copy number variants (CNVs) previously associated with schizophrenia, autism, intellectual disability, or developmental delay in individuals with AN. | Review (Systematic) | CNV's |
| Zeeland et al., 2014 | Worldwide | 3153 | Community (Adult, All Genders) | To identify associations between individual variants in the Epoxide Hydrolase 2 (EPHX2) gene and AN. | Cross-sectional (Correlational); | EPHX2 Gene Variants |
| Zhang et al., 2021 | Sweden | 33,140 | Outpatient (Adult, All Genders) | To explore the influence of family history of schizophrenia (as an indicator of genetic liability for schizophrenia) on clinical features and comorbidities in individuals with ED | Cross-Sectional | Family history of schizophrenia |
| **Gastrointestinal microbiota and autoimmune reactions** | | | | | | |
| Zerwas et al., 2017 | Denmark | 930,977 | Community (Mixed Cohort, All Genders) | To examine whether (1) autoimmune diseases were associated with increased hazard for subsequent eating disorders, and (2) whether eating disorders were associated with increased hazard for subsequent autoimmune disease. To examine (3) novel associations between autoinflammatory diseases and eating disorders, and (4) the odds for eating disorders were elevated for youth with a family history (parents, full and half siblings, and cousins) of autoimmune and autoinflammatory diseases. | Longitudinal (>10yr) | Autoimmune and autoinflammatory disease |
| Avila, Park, & Golden, 2019 | Worldwide | N/A | Mixed (Young adults, All genders) | To describe what is known about eating disorders in adolescents with chronic gastrointestinal and endocrine diseases, focusing on coeliac disease, inflammatory bowel disease, diabetes, and thyroid disorders | Review (Narrative) | Gastrointestinal and endocrine disease |
| Bächle et al., 2016 | Germany | 819 | Community (Mixed Cohort, All Genders) | To estimate the prevalence of disordered eating and IR in youths with early-onset Type 1 Diabetes of long duration and assess associations between disordered eating/insulin restriction and metabolic outcomes | Cross-Sectional | Insulin Restriction |
| Breton et al., 2016 | France | 95 | Outpatient (Adult, All Genders) | To measure plasma concentrations of ClpB in patients with anorexia nervosa (AN), bulimia nervosa (BN), and binge-eating disorder (BED) as well as in healthy participants | Cross-Sectional | ClpB protein |
| Breton, Dechelotte, & Ribet, 2019 | Worldwide | N/A | N/A | To present the current knowledge about the putative role of the gut microbiota in the pathogenesis, course and treatment of AN | Review (Narrative) | Intestinal microbiota |
| Carbone et al., 2020 | Worldwide | 17 studies | N/A | To update and critically analyse the existing literature on the possible role of altered microbiota in the etiopathogenesis and treatment of patients with EDs | Review (Systematic) | Microbiota (dysbiosis) |
| Conviser, Fisher, & McColley, 2015 | Worldwide | N/A | Mixed (Young adults, All genders) | To examine the relation between diet-treated CI and disordered eating and to determine the order of onset to infer directionality | Review (Systematic) | Children with chronic illness risk for ED |
| d'Emden et al., 2013 | Australia | 124 | Community (Adolescents, All Genders) | To describe the presence and types of disturbed eating behaviours and thoughts in a combined male/female Australian sample of adolescents with Type 1 diabetes mellitus. | Cross-Sectional | ED behaviours in Type I diabetes population |
| Duncan et al., 2017 | USA | 14477 | Community (Adult, All Genders) | To conduct a genome-wide association study of AN and to calculate genetic correlations with psychiatric, educational, and metabolic phenotypes. | Meta-Analysis | Metabolic Correlations |
| Eddy et al., 2015 | USA | 97 | Outpatient (Adult, Women) | To examine anorexigenic appetite-regulating hormones in individuals with anorexia nervosa to explore the role of these hormones in relation to the array of eating-related symptoms focusing expressly on anorexia nervosa subtypes | Cross-Sectional | Appetite regulatory hormones |
| Glenny et al., 2017 | Worldwide | N/A | N/A | To review and evaluate recently published scientific studies that explored the role of the intestinal microbiota in eating disorders | Review (Narrative) | Intestinal microbiota |
| Hanachi et al., 2019 | France | 55 | Inpatient (Young People, All Genders) | To determine an association between FIDs severity and dysbiosis of the intestinal microbiota in a severely malnourished patient population with AN undergoing enteral nutrition. | Cross-Sectional | Host-gut microbes |
| Nikniaz et al., 2021 | Worldwide | 23 studies for systematic review; 22 studies for meta-analysis | Community (Adults, All genders) | To review studies that assessed the prevalence and risk of eating disorders (EDs) in patients with celiac disease (CD) and vice-versa. | Systematic Review/ Meta-Analysis (combined) | Celiac disease |
| Nip et al., 2019 | USA | 2305 | Community (Young adults, All genders) | To examine the prevalence of disordered eating behaviours (DEB) and its associations with glycaemic control, insulin sensitivity (IS), and psychosocial functioning in a large, diverse cohort of youth and young adults with type 1 or type 2 diabetes | Cross-Sectional | Disordered eating behaviours, anthropometric measurements, insulin sensitivity, health outcomes |
| Peterson, Fischer, & Young-Hyman, 2015 | Worldwide | N/A | Mixed (Young adults, All genders) | To provide an updated literature review on prevalence, measurement, and correlates of disordered eating in youth with Type 1 diabetes (T1D), present a novel theoretical risk model (i.e., The Modified Dual Pathway Model) for disordered eating in youth with T1D incorporating psychosocial and physiological risk factors, and discuss clinical implications | Review (Narrative) | Prevalence, correlates, risk factors, and outcomes of disordered eating behaviour |
| Raevuori et al., 2014 | Finland | 2342 | Inpatient (Adult, All Genders) | To address the prevalence and incidence of autoimmune diseases in a large Finnish patient cohort with anorexia nervosa, bulimia nervosa, and binge eating disorder | Cross-Sectional | Autoimmune diseases |
| Reinehr et al., 2019 | German/Austrian/ Luxembourgian and Swiss | 31,556 | Community (Mixed Cohort, Girls) | To assess indications of eating disorders in girls with type 1 diabetes mellitus (T1DM) | Cross-Sectional | Pubertal age, no usage of pumps, migration background, HbA1c levels, DKA frequencies, hypoglycaemia |
| Santana et al., 2019 | Australia | 2977 | Community (Mixed Cohort, All Genders) | To investigate the association between self-reported diabetes (Type 1 or 2) with ED/DEB (binge eating, subjective binge eating or loss of control overeating, severe dieting and purging) weight/shape overvaluation, and health-related quality of life (HRQoL) in a household survey in South Australia. | Cross-Sectional | Diabetes Type 1 |
| Scheung et al., 2014 | Germany, Austria | 52,215 | Community (Adult, All Genders) | To compare clinical characteristics and outcome of type 1 diabetes mellitus (T1DM) between patients with and without a clinically recognized eating disorder (ED) | Cross-Sectional | Clinical characteristics of Type 1 diabetes patients with and without ED |
| Schorr & Miller, 2017 | Worldwide | N/A | N/A | To discuss the endocrine complications of anorexia nervosa, including dysregulation of hypothalamic–pituitary axis hormones, adipokines and appetite-regulating hormones | Review (Narrative) | Endocrine abnormalities |
| **Childhood and early adolescent exposures** | | | | | | |
| Afifi et al., 2017 | USA | 36,309 | Community (Adult, All Genders) | To examine the associations between child maltreatment, including harsh physical punishment, physical abuse, sexual abuse, emotional abuse, emotional neglect, physical neglect, and exposure to intimate partner violence, and eating disorders in adulthood among men and women | Cross-Sectional | Child maltreatment (sexual abuse, physical abuse and neglect, emotional abuse) |
| Ahren et al., 2012 | Sweden | 14,294 | Outpatient (Adult, All Genders) | To examine the associations between psychosocial factors and family background and incidence of anorexia nervosa (AN) in a Swedish cohort. | Longitudinal (>10yr) | Maternal education, psychosocial factors, family background |
| Allen et al., 2009 | Australia | 1597 | Community (Young adults, All genders) | To identify prospective predictors of eating disorders in a population-based sample of 14-year-old boys and girls, using previously collected antenatal, biomedical, familial, demographic, and psychosocial data | Repeated Measure (with follow-up) | Gender, Parent perception of overweight, Maternal BMI, low social related self-efficacy, neurocognitive difficulties, social problems |
| Allen et al., 2013 | Australia | 1383 | Community (Mixed Cohort, All Genders) | To (1) determine whether childhood risk factors for early onset binge eating and purging eating disorders also predict risk for later-onset binge eating and purging disorders, and (2) compare the utility of childhood and early adolescent variables in predicting later-onset disorders. | Longitudinal (>10yr) | Gender, parent-perceived child overweight, eating, weight and shape concerns |
| Allen et al., 2014 | Australia | 1160 | Community (Adult, All Genders) | To evaluate a prospective, mediational model of risk for the full range of binge eating and purging eating disorders, with attention to possible diagnostic differences | Longitudinal (>5yr) | Early adolescent eating, weight and shape concerns, parent-perceived childhood overweight |
| Allen et al., 2016 | Australia | 1297 | Community (Adolescents, All Genders) | To test for possible interactions between risk factors in the prediction of binge eating and purging eating disorders | Longitudinal (<5yr) | Gender, weight and eating concerns, externalising problems |
| Amianto et al., 2011 | Italy | 119 | Outpatient (Young People, All Genders) | To assess attachment and personality in anorexic women, non-affected siblings, and healthy controls, examining correlations with psychopathology. | Cross-Sectional | Maternal care and overprotection, self-transcendence, obsessive-compulsive traits, need for approval, pursuit of thinness, interpersonal distrust, social insecurity, low preoccupation with relationships |
| Amianto et al., 2018 | Italy | 172 | Outpatient (Young Adult, All Genders) | To assess childhood traumatic events in obese adults, with a specific focus on psychological abuse and emotional neglect, and to explore their correlations with psychopathological and personality characteristics in adulthood | Cross-Sectional | Emotional abuse and neglect, sexual abuse, physical abuse |
| Balantekin et al., 2021 | USA | 690 | Outpatient (Young People, Women) | To examine how eating disorder (ED) correlates, ED-related clinical impairment, general psychopathology, and ED diagnoses differ across weight statuses in a sample of university women with ED | Cross-Sectional | Weight |
| Belli et al., 2019 | Turkey | 241 | Outpatient (Young People, All Genders) | To assess childhood trauma history and dissociative symptoms in obese patients with binge eating disorder (BED) compared to obese patients without BED | Cross-Sectional | Obesity, dissociative scores, childhood physical abuse, childhood emotional abuse |
| Berge et al., 2013 | USA | 2348 | Community (Young adults, All genders) | To examine the associations between parent conversations about healthful eating and weight and adolescent disordered eating behaviors. | Cross-Sectional | Parental weight-related conversations |
| Berkowitz et al., 2016 | Sweden | 102 | Outpatient (Young People, All Genders) | To determine whether BMI or BMI trajectory differed between individuals who later developed adolescent-onset anorexia nervosa (AN) and a comparison group of healthy controls (HCs) between school grades 1 through 6. | Repeated Measure (without follow-up) | Childhood BMI |
| Brown et al., 2018 | USA | 114 | Inpatient (Adult, All Genders) | To examine whether ED diagnosis (AN-R vs. BN) moderates the association between alexithymia at treatment admission and change in emotion dysregulation at discharge. | Repeated Measure (without follow-up) | Alexithymia, emotion regulation, impulse control difficulties, access to emotion regulation strategies |
| Brown, Hochman, & Micali, 2020 | UK | 5812 | Community (Mixed Cohort, All Genders) | To examine longitudinal temperament/personality pathways of risk for purging and binge eating in youth stratified by sex using data from a large-scale prospective study | Longitudinal (>10yr) | Temperament, borderline personality features, sensation seeking, openness, extraversion, neuroticism, conscientiousness, agreeableness, depression |
| Buckner, Silgado, & Lewinsohn, 2010 | USA | 1709 | Community (Mixed Cohort, All Genders) | To examine the temporal sequencing of eating and anxiety disorders to delineate which anxiety disorders increase eating disorder risk and whether individuals with eating disorders are at greater risk for particular anxiety disorders | Longitudinal (>10yr) | Mood disorders |
| Canals, Sancho, & Arjia, 2009 | Spain | 1336 | Community (Adolescents, All Genders) | To investigate the relationship between parents' cognitive and behavioural dimensions and the risk of eating disorders in non-clinical adolescents | Repeated Measure (with follow-up) | Parental body dissatisfaction, drive for thinness, ineffectiveness, interoceptive awareness, social insecurity, perfectionism, child gender and BMI |
| Carretero-Garcia et al., 2017 | Spain | 300 | Inpatient (Young People, Women) | To analyse the association between traumatic experiences (TEs) and eating symptoms and their severity in a healthy group (HG) of students and an eating disorder group (EDG) | Cross-Sectional | Traumatic experiences, Child abuse |
| Caslini et al., 2016 | Worldwide | N/A | Mixed (Adults, All genders) | To estimate the association between distinct types of child abuse—sexual (CSA), physical (CPA), and emotional (CEA)—and different eating disorders (EDs). | Systematic Review/ Meta-Analysis (combined) | Child sexual abuse, child physical abuse, child emotional abuse |
| Coffino, Grilo, & Udo, 2020 | USA | 36145 | Community (Adult, All Genders) | To examine the relationship between childhood food neglect and DSM-5-defined AN, BN, and BED in a nationally-representative sample of U.S. adults, and to examine whether observed associations between childhood food neglect and EDs persist after adjusting for other ACEs and government financial assistance | Cross-Sectional | Childhood food neglect |
| Copeland et al., 2015 | USA | 1420 | Community (Mixed Cohort, All Genders) | To test whether bullying increases risk for eating disorder symptoms | Repeated Measure (with follow-up) | Bullying, family adversities, psychiatric status |
| de Barse et al., 2015 | Netherlands | 4851 | Parents or Carers (Women) | To examine whether a maternal history of eating disorders predicted mothers’ feeding practices and pre-schoolers’ emotional eating patterns | Repeated Measure (with follow-up) | Pressuring feeding strategies, child emotional overeating |
| Degortes et al., 2014 | Italy | 214 | Outpatient (Young Adult, All Genders) | To assess retrospectively the frequency and types of stressful life events preceding the onset of the eating disorder in a group of patients with binge eating disorder (BED) compared with a group of patients with bulimia nervosa (BN) and to examine the relationship between these events and psychopathological features | Cross-Sectional | Stressful life events |
| Dinkler et al., 2021 | Sweden | 5987 | Community (Mixed Cohort, All Genders) | To prospectively investigate autistic traits before and after the first diagnosis of AN | Longitudinal (>10yr) | Autism |
| Dörsam et al., 2019 | Worldwide | N/A | Mixed (Adults, Women) | To provide a synthesis of evidence regarding overall nutrition and related issues of pregnant women with a history of EDs | Review (Systematic) | Maternal dietary intake during pregnancy, Maternal ED diagnosis |
| Ellis et al., 2018 | USA | 1339 | Community (Adult, All Genders) | To examine potential predictors of adult picky eating | Cross-Sectional | Parental feeding practices (pressure to eat, encouragement of healthy eating), disgust sensitivity, aversive food event |
| Fischer, Stojek, & Hartzell, 2010 | USA | 489 | Community (Adult, Women) | To examine the effect of recent adult sexual assault on current eating disorder symptoms when controlling for the effects of multiple forms of childhood abuse | Cross-Sectional | Childhood emotional abuse, childhood physical abuse, childhood sexual abuse, recent sexual assault |
| Forrest et al, 2021 | USA | 36146 | Community (Adult, All Genders) | To examine whether having histories of adverse childhood experiences (ACEs) and EDs augments lifetime risk for suicide attempts | Cross-Sectional | Adverse childhood experiences, suicide attempts |
| Gonçalves et al., 2014 | Portugal | 180 | Outpatient (Adult, Women) | To evaluate the occurrence of life events preceding the onset of eating problems in bulimia nervosa patients | Cross-Sectional | Life events (stress, eating criticism, weight and shape criticism) |
| Gonçalves et al., 2016 | Portugal | 180 | Outpatient (Young Adult, Women) | To (i) replicate the uncontested retrospective correlates for BN and clarify the role of factors with inconsistent findings, and (ii) evaluate the strength of these factors in a different culture. | Cross-Sectional | Maternal expectations, negative attitudes about parental weight, obesity, self-harm, family conflicts, feeling fat in childhood |
| Haines et al., 2010 | USA | 10540 | Community (Adolescents, All Genders) | To identify shared risk and protective factors for purging, binge eating, and overweight | Cross-Sectional | Concern for weight, dieting, parental weight-related teasing, family meal frequency |
| Hayes et al., 2021 | Worldwide | 15 studies for systematic review; 12 studies for meta-analysis | Community (Adults, All genders) | To synthesize evidence on the relationship between sexual harassment and eating disorder psychopathology, as well as mediating and moderating factors that contribute to this relationship | Systematic Review/ Meta-Analysis (combined) | Sexual harassment, gender, posttraumatic stress, anxiety |
| Haynos et al., 2016 | USA | 2,516 | Community (Adolescents, All Genders) | To examine longitudinal risk factors and short-term risk correlates for the development of extreme forms of restrictive eating among adolescent dieters. | Longitudinal (>5yr) | Depression, self-esteem, poor family communication/caring, maternal dieting, body image issues, weight-related teasing, peer dieting |
| Hazzard et al., 2019 | USA | 14322 | Community (Mixed Cohort, All Genders) | To examine associations between childhood maltreatment patterns and eating disorder symptoms in young adulthood | Longitudinal (>5yr) | Childhood physical neglect, physical abuse, sexual abuse, |
| Hicks-White, Pratt, & Cottrill, 2018 | USA | 182 | Outpatient (Young People, All Genders) | To describe the prevalence of different types of traumas (Childhood physical abuse (CPA), childhood sexual abuse (CSA), bullying, domestic violence, motor vehicle accident, neighbourhood violence, war, terrorism, medical procedures, national disaster, and significant death/loss) in a clinical sample of adolescents in outpatient treatment for an eating disorder (AN, BN, and eating disorder not otherwise specified (ED NOS)) | Cross-Sectional | Traumatic events (bullying, domestic violence) |
| Huemer et al., 2012 | Austria | 332 | Outpatient (Young People, All Genders) | To examine retrospective correlates of nonshared family environment prior to onset of disease, by means of multiple familial informants, among anorexia and bulimia nervosa patients | Cross-Sectional | Emotional connectedness, individual autonomy, |
| Hymowitz, Salwen, & Salis, 2017 | USA | 598 | Community (Adult, All Genders) | To evaluate a model of Disordered Eating (DE) and weight difficulties in which negative self-perception mediate the relationship between emotional abuse (EA) and DE, and DE predicts body mass index (BMI) in a population of emerging adults; and utilize recursive partitioning to confirm whether EA can predict DE, and establish cut-offs on a measure of EA that can predict risk of night eating syndrome (NES), binge eating disorder (BED), or unhealthy weight control behaviour (UWC) | Cross-Sectional | Disordered eating, childhood emotional; abuse, negative self-perceptions |
| Imperatori et al., 2016 | Italy | 301 | Community (Adult, Women) | To assess in overweight and obese women, (i) the association between child trauma (CT) and food addiction (FA) controlling for potential confounding variables (e.g., BMI, psychopathology, etc.), (ii) CT in patients with cooccurrence of FA and other dysfunctional eating patterns (i.e., clinical level of binge eating) versus patients with “singular” forms of dysfunctional eating patterns (i.e., patients who only engage in binge eating or who only have FA) | Cross-Sectional | Food addiction, childhood trauma, binge eating, anxiety, depression |
| Jones et al., 2017 | Worldwide | N/A | N/A | To summarize evidence for the role of foetal programming in eating disorder aetiology through review of studies demonstrating specific obstetric complications and later eating risk of anorexia or bulimia. | Review (Systematic) | Foetal programming |
| Klein et al., 2017 | USA | 799 | Community (Adult, All Genders) | To examine the impact of maternal and paternal dieting and comments about weight and eating, as reported by late adolescents, on the eating pathology of those late adolescents at 20-year follow-up | Longitudinal (>10yr) | Parental dieting, comments on weight and eating |
| Knoph Berg et al., 2011 | Norway | 45644 | Parents or Carers (Women) | To identify factors associated with incidence and course of broadly defined binge eating disorder (BED) in pregnancy. | Cross-Sectional | Lifetime sexual, physical, emotional abuse, depression, anxiety, life satisfaction, self-esteem, partner relationship satisfaction, smoking, alcohol use, social support, weight-related factors |
| Kothari et al., 2014 | UK | 1564 | Community (Children, All Genders) | To investigate cognitive development in children at high risk at 18 months (Griffiths Mental Development Scale) and 4 years old (Wechsler Preschool and Primary Scale of Intelligence—Revised), in comparison with children not at risk, using a general population sample, the Avon Longitudinal Study of Parents and Children | Longitudinal (<5yr) | Cognitive development |
| Kothari et al., 2014 | UK | 446 | Parents or Carers (Women) | To determine whether 2D:4D ratio, a marker for in utero testosterone exposure, is associated with risk for ED in a large population-based cohort: the Avon Longitudinal Study of Parents and Children (ALSPAC) | Cross-Sectional | Prenatal testosterone exposure, Maternal lifetime ED |
| Krug et al., 2009 | Europe | 1664 | Outpatient (Young People, All Genders) | To examine whether there is an association between individual and family eating patterns during childhood and early adolescence and the likelihood of developing a subsequent eating disorder (ED) | Cross-Sectional | Childhood eating behaviour (retrospective) |
| Krug et al., 2016 | Australia | 1300 | Community (Adolescents, All Genders) | To investigate the interactions between low parental warmth and monitoring at age 13-14 years and disordered eating attitudes and behaviours at age 15-16 years | Longitudinal (<5yr) | Parenting practices (warmth, monitoring) |
| Krug et al., 2015 | Europe | 1264 | Outpatient (Young People, All Genders) | To examine a new socio-family risk model of Eating Disorders (EDs) using path-analyses | Cross-Sectional | Parenting styles, family, media and peer influences, body dissatisfaction |
| Larsen et al., 2017 | Denmark | 495244 | Community (Adult, Women) | To examine the association between a range of measures of early childhood adversity and later risk of AN, BN, and EDNOS separately, and to examine the effect of multiple adversities and compare the risk estimates for eating disorders to those for other psychiatric disorders with overlapping symptomatology and traits, that is, major depressive disorder (MDD), anxiety disorder, and obsessive compulsive disorder (OCD) | Cross-Sectional | Family disruption, residential instability, placement in out-of-home care, familial death, parental somatic illness, parental psychiatric illness, parental disability, severe parental criminality, parental substance use disorder |
| Latzer et al., 2020 | Israel | 426 | Outpatient (Adult, Women) | To compare women with binge eating disorders or bulimia nervosa with and without night eating syndrome regarding childhood maltreatment and psychopathology relative to healthy controls | Cross-Sectional | Childhood trauma, self-esteem, psychopathology |
| Lebow, Sim, & Kransdorf, 2015 | USA | 179 | Outpatient (Young People, All Genders) | To clarify the less-obvious relationship between overweight or obesity and eating disorders characterized by dietary restriction and/or weight loss | Cross-Sectional | Obesity, Overweight |
| Lie et al., 2021 | Norway | 890 | Outpatient (Young People, All Genders) | To investigate the association between bullying exposure and eating disorders (EDs) | Cross-Sectional | Bullying history |
| Loth et al., 2014 | USA | 1902 | Community (Young adults, All genders) | To identify personal and socioenvironmental factors associated with the persistence of dieting or disordered eating from adolescence to young adulthood and factors associated with the initiation of dieting or disordered eating during young adulthood | Longitudinal (>10 years) | Disordered eating behaviours, self-esteem, weight concern, body satisfaction, depression, weight importance, health concern, parental weight concerns, peer dieting, weight teasing, family communication |
| Loth et al., 2015 | USA | 3072 | Community (Young Adult, All Genders) | To examine how both overweight and non overweight adolescent girls and boys fared from 1999 to 2010 in terms of disordered eating behaviours and psychosocial wellbeing. | Cross-Sectional | Overweight status |
| Lydecker et al., 2012 | USA, Norway, Sweden | 21861 | Community (Adult, All Genders) | To examine the hypothesis that prenatal exposure to sex hormones influences twins' risk for eating disorders based on co-twin sex | Cross-Sectional | Sex, prenatal sex hormones, co-twin sex |
| Machado et al., 2016 | Portugal | 331 | Outpatient (Adult, Women) | To identify retrospective correlates that distinguish AN and BN | Cross-Sectional | Paternal expectations, excessive family importance placed on fitness/keeping in shape, negative consequences due to adolescent overweight |
| Marco et al., 2018 | Spain | 80 | Outpatient (Young People, All Genders) | (1) To analyse whether cyberbullying victimization would be positively associated with ED psychopathology, body dissatisfaction, and depression and (2) to analyse whether the model composed of appearance evaluation, depression, and cyberbullying victimization would be a significant predictor of ED psychopathology and explain more variance in ED symptoms than appearance evaluation and depression alone | Cross-Sectional | Cyberbullying victimisation, gender, BMI, appearance evaluation, depression |
| Marzola et al., 2021 | Worldwide | 37 studies | Mixed (Adults, Women) | [To systematically and critically review the literature on the association of prenatal and perinatal factors with the onset of EDs in the offspring, updating previous findings and focusing on anorexia nervosa (AN) and bulimia nervosa (BN).](https://go-gale-com.ezproxy.library.sydney.edu.au/ps/i.do?p=AONE&u=usyd&id=GALE%7CA656116505&v=2.1&it=r) | Review (Systematic) | Prenatal and perinatal factors |
| Micali & Treasure, 2009 | Worldwide | N/A | Community (Adults, Women) | To review the literature on the biological effects of a maternal eating disorder (ED) (and relevant comorbidities) in pregnancy on mothers and in particular on the foetus, and to highlight possible mechanisms of risk for long-term consequences in the offspring | Review (Narrative) | Maternal ED in pregnancy |
| Micali et al., 2011 | UK | 10,902 | Community (Mixed cohorts, All genders) | To determine the role of maternal anxiety and depression in mediating the risk for feeding difficulties in infants of women with ED | Longitudinal (<5yr) | Maternal anxiety and depression in pregnancy and post-partum, child temperament |
| Micali et al., 2014 | UK | 143 | Community (Mixed Cohort, All Genders) | To investigate the prevalence of eating disorder (ED) psychopathology, neuropsychological function, structural brain correlates and risk mechanisms in a prospective cohort of very preterm (VPT) young adults | Longitudinal (>10yr) | Preterm birth, executive function, gray and white matter volume, caesarean delivery |
| Micali et al., 2017 | UK | 5658 | Community (Adult, Women) | To investigate the lifetime and 12-month prevalence of EDs and lifetime health service use and to identify childhood, parenting, and personality risk factors | Longitudinal (<5yr) | Childhood sexual abuse, external locus of control, maternal care, childhood life events, interpersonal sensitivity |
| Monteleone et al., 2015 | Italy | 73 | Outpatient (Adult, Women) | To examine possible associations between childhood traumatic experiences and HPA-axis functioning in adult patients with AN or BN and in healthy adult women | Review (Narrative) | Neuroendocrinology and brain imaging of reward in eating disorders |
| Monteleone et al., 2019 | Italy | 228 | Outpatient (Young Adult, All Genders) | To explore the psychological pathways through which childhood maltreatment (CM) experiences promote eating disorder (ED) core symptoms | Cross-Sectional | Childhood maltreatment, interoceptive awareness, drive to thinness, ineffectiveness |
| Neumark-Sztainer et al., 2010 | USA | 356 | Community (Adolescents, Girls) | To examine associations between family variables (parent weight talk, parent dieting, and family weight-teasing) and adolescent girls' weight status, body satisfaction, and disordered eating behaviours. | Cross-Sectional | Parent weight talk and dieting, family weight-teasing |
| Nicholls et al., 2009 | UK | 16,567 | Community (Mixed Cohort, All Genders) | To examine whether previously identified childhood risk factors for anorexia nervosa (AN) predict self-reported lifetime AN by age 30 years in a prospective birth cohort | Longitudinal (>10yr) | Gender, infant feeding problems, maternal depressive symptoms, history of undereating |
| Nicholls et al., 2016 | UK | 11,261 | Community (Adult, All Genders) | To examine whether previously identified childhood risk factors for bulimia or compulsive eating (BCE) predict self-reported lifetime BCE by age 30 years in a prospective birth cohort | Longitudinal (>10yr) | Gender, self-esteem, maternal education, SES |
| Quilliot et al., 2019 | France | 1484 | Outpatient (Adult, All Genders) | To evaluate the association between childhood or early adulthood traumatic experiences and adulthood binge eating disorder (BED) in 326 male and 1158 female patients | Cross-Sectional | Child abuse, domestic violence, neglect |
| Reba-Harrelson et al., 2010 | Norway | 13,006 | Parents or Carers (Women) | To explore the impact of broadly defined eating disorders on maternal feeding practices and children's eating behaviours and psychological symptoms in a sample of 13,006 births in Norway | Longitudinal (<5yr) | Maternal ED diagnosis |
| Rossman et al., 2020 | USA | 20,745 | Community (Mixed Cohort, All Genders) | To describe the relationship between adoption status and behavioural eating disorder (ED) symptoms, and to examine potential correlates of ED symptoms that are unique to adopted individuals. | Longitudinal (>5yr) | Adoption, contact with biological parent, age of adoption, foster care |
| Rubin et al., 2021 | USA | 201 | Community (Mixed Cohort, All Genders) | To assess associations between weight-based teasing (WBT) and disinhibited and disordered eating among non-treatment seeking youth ages 8-17y, and whether negative affect mediated these relationships | Cross-Sectional | Weight-based teasing, depression, anxiety |
| Smith et al., 2016 | USA | 204 | Outpatient (Young People, Women) | To explore the association between specific forms of childhood abuse and neglect with lifetime suicide attempts in women with bulimia nervosa (BN) | Cross-Sectional | Lifetime suicide event, childhood physical, emotional and sexual abuse, neglect |
| Saltzman & Liechty, 2016 | Worldwide | N/A | Mixed (Children, All genders) | To identify family correlates of binge eating in children (C-BE) aged 12 and under | Review (Systematic) | Family weight teasing, parent emotional unresponsiveness, parent weight, education, SES, race-ethnicity, parent disordered eating, weight/thinness concern, harsh discipline, maternal dieting, attachment security, mealtimes and feeding practices |
| Solmi et al., 2020 | Worldwide | 9 meta-analysis | N/A | To grade the evidence about risk factors for eating disorders (anorexia nervosa, bulimia nervosa, and binge eating disorder) with an umbrella review approach | Systematic Review/ Meta-Analysis (combined) | Childhood sexual abuse, appearance-related teasing victimisation |
| St-Hilaire et al., 2015 | Canada | 54 | Community (Adolescents, All Genders) | To study the influences of prenatal maternal stress on later disordered eating in exposed offspring. | Longitudinal (>10yr) | Prenatal maternal stress |
| Stice & Desjardins, 2018 | USA | 1271 | Community (Mixed Cohort, Girls) | To conduct exploratory analyses regarding the interactions between risk factors in predicting future onset of AN, BN, BED, and PD, and to characterize the interacting relations between the risk factors for future onset of eating disorders by virtue of body dissatisfaction | Repeated Measure (with follow-up) | BMI, Body dissatisfaction, overeating, positive expectations for thinness, dieting, thin-ideal internalisation, negative affect |
| Su et al., 2015 | Denmark, Sweden | 5,102,034 | Community (Children, All Genders) | To examine whether prenatal stress following maternal bereavement is associated with ED in infants and toddlers | Longitudinal (<5yr) | Prenatal stress |
| van Eeden et al., 2021 | The Netherlands | 2229 | Community (Adolescents, All Genders) | To explore risk factors for the development of eating pathology in a prospective, community cohort study covering preadolescence through young adulthood. | Longitudinal (>10yr) | Pregnancy and perinatal factors, sociodemographic variables, eating and weight-related factors, psychological functioning, stressful experiences and family factors |
| Veses et al., 2011 | Spain | 195 | Community (Adolescents, All Genders) | To assess the association between excessive body fat and the risk of eating disorders | Cross-Sectional | Overweight |
| Watkins, Cooper, & Lask, 2012 | UK | 66 | Parents or Carers (Women) | To assess whether the prevalence of a maternal history of eating disorder differentiated early onset eating disturbance groups (AN, FAED and SE). | Cross-Sectional | Maternal eating disorder history |
| Watson et al., 2013 | Norway | 77,267 | Community (Adult, Women) | To internally validate the statistical modelling of incidence, remission and continuation used in women during early pregnancy using MoBa (The Norwegian Mother and Child Cohort Study) questionnaire | Cross-Sectional | Pregnancy |
| Watson et al., 2015 | Norway | 69,030 | Community (Adult, Women) | To internally validate the relationship between psychosocial characteristics and broadly defined bulimia nervosa during early pregnancy, including factors associated with continuation, incidence and remission between psychosocial factors and bulimia nervosa (BN) outcomes during pregnancy | Cross-Sectional | Relationship satisfaction, self-esteem, life satisfaction, physical abuse, anxiety, depression |
| Watson, O'Brien, & Sadeh-Sharvit, 2018 | Worldwide | N/A | Community (Young adults, All genders) | To address the question of whether the offspring of parents with past or present eating disorders have adverse outcomes | Review (Narrative) | Paediatric, ED related behaviours, ED risk and psychological health profile of offspring of ED parents |
| Watson et al., 2019 | Norway | 46,373 | Parents or Carers (Women) | To investigate whether prenatal/perinatal complications are associated with lifetime eating disorders in women | Longitudinal (>10yr) | Birth weight, born large for gestational age |
| Yilmaz et al., 2019 | UK | 1502 | Community (Mixed Cohort, All Genders) | To examine whether childhood body mass index (BMI) trajectories are prospectively associated with later eating disorder (ED) diagnoses | Longitudinal (>10yr) | Development growth trajectory, BMI trajectory |
| **Personality traits and comorbid mental health conditions** | | | | | | |
| Adambegan et al., 2012 | Austria | 83 | Outpatient (Young People, Women) | To examine for differences within siblings before the onset of AN—restricting type (AN-R) or bulimia nervosa (BN) | Longitudinal (<5yr) | Internalising and externalising symptoms |
| Amianto et al., 2012 | Italy | 363 | Outpatient (Adult, All Genders) | To explore anger correlation with bulimic symptoms and to test the mediation power of anger between personality and eating psychopathology | Cross-Sectional | Anger, self-directedness, cooperativeness, impulsiveness, harm avoidance, social insecurity, interpersonal distrust |
| Baker et al., 2013 | USA, Canada, England, Germany | 767 | Outpatient (Adult, Women) | To assess the temporal pattern of comorbid anorexia nervosa (AN) and alcohol use disorder (AUD) and the impact this ordering has on symptomatology and associated features | Cross-Sectional | Alcohol use disorder, impulsivity scores, depression, borderline personality disorder |
| Bartlett & Mitchell, 2015 | Worldwide | 33 studies | Mixed (Adults, All genders) | To examine prevalence estimates and associated symptomatology of EDs among military and veteran men and women and to identify factors that may put these individuals at risk for the development of an ED for the purposes of improving detection, intervention, and treatment. | Review (Systematic) | Military employment, sexual trauma |
| Boucher et al., 2018 | Canada | 50 | Outpatient (Adult, Women) | To deepen our understanding of the relationship between pathological narcissism and eating pathology by examining the predictive value of specific facets that compose grandiose and vulnerable narcissism while taking into account self-esteem and ED diagnoses (AN or BN) | Cross-Sectional | Narcissism, self-esteem |
| Brewerton et al., 2020 | USA | 642 | Inpatient (Adult, All Genders) | To determine the association of traumatic events and posttraumatic stress disorder with greater eating disorder and comorbid symptom severity in residential eating disorder treatment centers | Cross-Sectional | PTSD, traumatic events |
| Carr et al., 2021 | Worldwide | 32 studies | N/A | To identify and synthesize available behavioural studies of impulsivity and compulsivity among individuals with binge eating disorder (BED) | Review (Systematic) | Impulsivity, compulsivity |
| Castellini, Rossi, & Ricca, 2020 | Worldwide | 15 studies | Mixed (Adults, All genders) | To overcome the conceptualization of a comorbidity between eating disorders and sexual dysfunctions, which limits the coexistence of these two conditions to a merely descriptive level, aiming at clarifying the nature of this relationship in terms of psychopathological meaning | Review (Narrative) | Sexual problems, impulsivity |
| Chapman & Woodman, 2015 | Worldwide | 31 studies | Community (Young adults, All genders) | To apply meta-analytic procedures to investigate whether male athletes are more at risk of disordered eating relative to male controls, and whether there are moderators that might explain some of the inconsistent findings in the literature | Meta-Analysis | Male athlete |
| Cheng et al., 2019 | USA | 1117 | Community (Mixed Cohort, Girls) | To examine the predictive effects of overeating, fasting, excessive exercise, functional impairment, and mental health service usage on eating disorders based on evidence that these factors predict future onset of eating disorders | Repeated Measure (with follow-up) | Ethnicity |
| Ciarma & Mathew, 2017 | Australia | 282 | Community (Adult, All Genders) | To explore whether reactivity to stress in social settings and self-esteem mediate the link between social anxiety and disordered eating in a community sample | Cross-Sectional | Stress reactivity, social anxiety |
| Dakanalis et al., 2014 | Italy | 361 | Community (Adolescents, Girls) | To evaluate prospectively and compare the original and the extended dual pathway model of binge eating. | Longitudinal (<5yr) | Negative affect, body dissatisfaction, dieting, interoceptive deficits, emotional eating |
| Davico et al., 2019 | Italy | 73 | Inpatient (Young People, All Genders) | To examine whether adolescents who present with AN+NSSI differ from those with only AN with respect to intelligence, psychopathology, global functioning, or temperament and character traits. | Cross-Sectional | Non-suicidal self-injury |
| Degortes et al., 2014 | Italy | 267 | Outpatient (Young People, All Genders) | To study the prevalence of childhood obsessive–compulsive traits in patients with lifetime AN, their unaffected sisters and healthy women | Cross-Sectional | Childhood obsessive-compulsive traits, body image distortion |
| Dellava et al., 2010 | USA | 326 | Community (Adult, Women) | To examine childhood measures of temperament that index anxiety proneness to determine their association with lifetime lowest BMI (lowest BMI) in a cohort of individuals with AN | Cross-Sectional | Eating pathology, childhood temperament characteristics |
| Dell'Osso et al., 2018 | Italy | 138 participants with EDs & 160 healthy controls | Community (Adults, All genders) | To assess the presence of subthreshold autism spectrum symptoms, by means of a recently validated instrument, in a sample of participants with EDs, particularly comparing participants with or without binge eating behaviours | Case-control | Autism spectrum psychopathology, eating pathology |
| Dougherty et al., 2020 | USA | 50 | Community (Adult, Women) | To investigate the association between emotional regulation (ER) flexibility and disordered eating in a non-clinical sample using a well-established ER choice task paradigm that focuses on the ability to flexibly select between distraction and reappraisal |  | Emotion regulation flexibility |
| Drieberg et al., 2019 | Australia | 231 | Outpatient (Young People, All Genders) | To investigate the relationships between anxiety/depression, perfectionism and eating disorder symptoms in children and adolescents with eating disorders | Cross-Sectional | Anxiety, depression, perfectionism |
| Farstad & McGeown, 2016 | Worldwide | N/A | Mixed (Adults, All genders) | (1) To provide a systematic review of the literature linking personality and EDs, with an emphasis on publications since 2004, (2) to conduct a meta-analysis of personality disorder (PD) prevalence rates among individuals with specific ED diagnoses, including previously little-studied anorexia nervosa, binge-eating/purging type (AN-BP) and binge eating disorder (BED), and (3) to synthesize the literature to highlight the extent of current knowledge on personality and EDs, existing gaps in understanding, and future research directions | Systematic Review/ Meta-Analysis (combined) | Perfectionism, neuroticism, avoidance motivation, heightened sensitivity to social rewards, extraversion, self-directedness, impulsivity, avoidant, obsessive-compulsive personality, borderline personality disorder, paranoid |
| Forman-Hoffmann et al., 2012 | USA | 1004 | Outpatient (Adult, Women) | To examine lifetime self-reported and diagnosed eating disorders (EDOs) and related attitudes and behaviours in a sample of Veterans Affairs (VA)-enrolled women veterans, (2) to examine the association between sexual trauma during various stages of the life course (childhood, during military service, and lifetime), post-traumatic stress disorder (PTSD), and EDOs. | Cross-Sectional | PTSD, sexual trauma |
| Fornaro et al., 2010 | Italy | 148 | Outpatient (Adult, Women) | To evaluate the impact of Eating Disorders (EDs) lifetime co-morbidity among female with Bipolar Disorders (BDs) and to compare clinical and cognitive features among EDs subgroups | Cross-Sectional | Bipolar disorder |
| Friborg et al., 2014 | Worldwide | 20 studies (1581) | Mixed (Adults, All genders) | To identify the proportion of comorbid personality disorders (PDs) in patients with eating disorder not otherwise specified (EDNOS) and binge eating disorder (BED) | Meta-Analysis | Personality disorder |
| Godart et al., 2015 | France | 271 | Mixed (Mixed cohorts, All genders) | To determine: (1) the frequency of mood disorders in all AN and BN subject groups, according to DSM-IV criteria; (2) whether mood disorders are significantly more frequent in ED patients than in the general population; (3) in cases with comorbidity, the relative chronology of onset of ED and mood disorder | Cross-Sectional | Mood disorder |
| Grilo et al., 2012 | USA | 105 | Outpatient (Adult, Women) | To examine the frequency and significance of comorbid posttraumatic stress disorder (PTSD) in ethnically diverse obese patients with binge eating disorder (BED) seeking treatment for obesity and binge eating in primary care | Cross-Sectional | PTSD |
| Grilo, White, & Masheb, 2009 | USA | 404 | Outpatient (Adult, All Genders) | To assess DSM-IV lifetime and current psychiatric disorder comorbidity in patients with binge eating disorder (BED) and to examine associations of comorbidity with gender, selected historical obesity-related variables, and current eating disorder psychopathology | Cross-Sectional | Psychiatric disorder, mood disorder, substance use disorder, anxiety disorder, |
| Holland, Bodell, & Keel, 2013 | USA | 1320 | Community (Adult, All Genders) | To identify psychological factors that predict onset and maintenance of eating disorders | Longitudinal (>10yr) | Perfectionism, interpersonal destruct, maturity fears |
| Howard et al., 2020 | Worldwide | 38 studies | Community (Adults, All genders) | To systematically appraise cross-sectional research that compared the cognitive performance of individuals in the acute phase of bulimia nervosa (BN) and/or anorexia nervosa (AN) to healthy controls on measures of impulsivity and compulsivity | Review (Systematic) | Impulsivity, compulsivity |
| Huke et al., 2013 | Worldwide | 8 studies | Community (Adults, All genders) | To conduct a systematic review investigating the prevalence of autism spectrum disorder in its entirety in eating disordered populations. | Review (Systematic) | Prevalence of autism spectrum disorder in ED population |
| Isomaa, Backholm, & Birgegard, 2015 | Sweden | 843 | Outpatient (Adult, All Genders) | To investigate the association between PTSD and ED severity, to focus on the mediating role of psychological distress for the above-mentioned association, and to assess the role of timing of trauma in relation to emergence of ED. | Cross-Sectional | PTSD, psychological distress |
| Jahrami et al., 2021 | Bahrain | 210 | Community (Mixed Cohort, All Genders) | To examine the risk of ED among children and adolescents with ADHD | Cross-Sectional | ADHD, gender, weight |
| Joyce et al., 2012 | Australia | 202 | Community (Adult, Women) | To investigate the mediating effect of shape and weight overvaluation and conditional goal setting on the relationship between perfectionism and eating pathology among women in the general community | Cross-Sectional | Perfectionism, conditional goal setting, shape and weight overevaluation |
| Kaisari, Dourish, & Siggs, 2017 | Worldwide | 72 studies | N/A | To evaluate the potential association between ADHD symptomatology and disordered eating behaviour | Review (Systematic) | ADHD symptoms, disordered eating behaviours |
| Lee-Winn et al., 2016 | USA | 10,028 | Community (Adolescents, All Genders) | To assess associations among maladaptive coping styles, combined neuroticism and impulsivity, and binge eating in the general United States adolescent population. | Cross-Sectional | Neuroticism, impulsivity, maladaptive coping styles |
| Levinson et al., 2017 | USA | 196 | Outpatient (Adult, All Genders) | To identify which symptoms of BN are central to the disorder and to test which symptoms of anxiety and depression are most strongly related to symptoms of BN | Cross-Sectional | Anxiety, depression, fear of weight gain, physical sensation symptoms, |
| Levinson et al., 2019 | USA | 254 | Outpatient (Young People, Women) | To test if cognitive-behavioural dimensions of OCD differed between AN and Atypical AN, and to detect which unique dimensions of OCD (e.g., obsessing, checking) were related to specific ED symptoms (e.g., drive for thinness, bulimia symptoms, body dissatisfaction, and overall eating pathology) | Cross-Sectional | OCD symptomology |
| Levinson, Byrne, & Rodebaugh, 2016 | USA | 300 | Community (Adult, Women) | To test if state shame and guilt were related to both social anxiety (SA) and bulimia nervosa (BN) symptoms (a) cross-sectionally and (b) prospectively over the course of two months | Repeated Measure (without follow-up) | Social anxiety disorder, shame, guilt, negative affect |
| Lilienthal, 2013 | USA | 139 | Community (Adult, Women) | To investigate the presence of a sign and domain effect in delay discounting of two novel hypothetical outcomes: the respondent’s body weight and her “looks” (e.g., complexion), and to explore the relationship between decision-making impulsivity in disorder-relevant contexts and one’s risk for low body esteem and anorexia nervosa | Cross-Sectional | Impulsivity |
| Lloyd et al., 2019 | Worldwide | 8 studies | Mixed (Young adults, All genders) | To establish whether anxiety predicts subsequent anorexia nervosa onset and maintenance. | Review (Systematic) | Anxiety |
| Lunde et al., 2009 | Norway | 201 | Inpatient (Adult, All Genders) | To study the relationship between eating disorders and major affective disorders in a sample of patients diagnosed with major depressive disorder (MDD) or bipolar disorder (BP) | Cross-Sectional | Affective disorders, affective temperaments |
| Mayer et al., 2009 | The Netherlands | 301 | Community (Adolescents, Girls) | To examine the associations between general risk factors and eating behaviour problems and to explore whether these variables show direct or indirect relations to disturbed eating. | Cross-Sectional | Body dissatisfaction, attachment style, self-esteem, social anxiety, depression |
| McDonald, Rossell, & Philipou, 2019 | Worldwide | 39 studies | Mixed (Adults, All genders) | To systematically review comorbid eating disorder (ED) diagnoses within bipolar disorder (BD) populations | Review (Systematic) | Bipolar disorder |
| McElroy et al., 2016 | USA | 1092 | Outpatient (Adult, All Genders) | To determine prevalence rates and clinical correlates of current DSM-5 eating disorders in patients with bipolar disorder (BP). | Cross-Sectional | Bipolar disorder |
| Mehl et al., 2019 | USA | 1153 | Community (Adult, Women) | To disaggregate the global measure of psychosocial functioning to examine which domain(s) and individual item(s) of psychosocial impairment showed the strongest relations to future onset of any eating disorder to advance knowledge of this general eating disorder risk factor | Cross-Sectional | Psychosocial impairment, loneliness |
| Melo et al., 2018 | Brazil | 120 | Outpatient (Adult, All Genders) | To identify night eating syndrome (NES) in euthymic bipolar disorder (BD) patients. | Cross-Sectional | Depression, anxiety, mania, functioning, physical activity, sleep quality, insomnia |
| Messer, Anderson, & Linardon, 2021 | USA, UK, Australia, Canada | 1101 | Community (Adult, All Genders) | To examine the unique role of mindfulness and self-compassion on eating disorder (ED) psychopathology and functional impairment, and compare levels of mindfulness and self-compassion between health controls and individuals categorized with bulimia nervosa (BN), and binge-eating disorder (BED) | Cross-Sectional | Self-compassion, mindfulness |
| Micali et al., 2011 | UK | 231 | Outpatient (Young People, All Genders) | To determine the prevalence of eating disorder (ED) at follow-up and clinical predictors in a longitudinal clinical sample of adolescents/young adults diagnosed with obsessive-compulsive disorder (OCD) in childhood | Longitudinal (>5yr) | OCD, family history of ED, |
| Mitchell et al., 2014 | USA | 492 | Outpatient (Adult, Women) | To describe rates of EDs and their correlates in a sample of female veteran primary care patients | Cross-Sectional | Military employment, PTSD |
| Navarro-Haro et al., 2015 | Spain | 68 | Outpatient (Adult, Women) | To examine the relationship of two emotion regulation strategies (i.e., expressive suppression and cognitive reappraisal) and dissociation with non-suicidal self-injury (NSSI) in women with borderline personality disorder (BPD) and comorbid eating disorder (ED) | Cross-Sectional | Emotion regulation, non-suicidal self-injury |
| Nazar et al., 2016 | Worldwide | 22 studies | Community (Adults, All genders) | To perform a meta-analysis of studies to investigate the risk of comorbidity with an ED (AN, BN, or BED) in ADHD individuals or eating disorder symptoms (binge eating or LOC eating) in ADHD paediatric samples. | Systematic Review/ Meta-Analysis (combined) | Risk of ADHD, ADHD symptoms, disordered eating behaviours |
| Orhan et al., 2011 | Turkey | 334 | Outpatient (Adult, All Genders) | To identify the rate of night eating syndrome (NES) in a depressed population. | Cross-Sectional | Depression, BMI, gender, education status |
| Pearson et al., 2014 | Worldwide | N/A | Mixed (Young adults, Women) | To provide an integrative review of existing risk factors and models for bulimia nervosa (BN) in young girls | Review (Narrative) | Negative mood, Negative urgency, Psychosocial learning |
| Pearson et al., 2016 | USA | 133 | Outpatient (Adult, Women) | To examine several empirically relevant dimensions of personality psychopathology in relation to specific risky behaviours, both alone (e.g., substance misuse) and in combination with other (e.g., substance misuse and self-harm), in a sample of women with BN who reported on behaviours in real time in their natural environments | Repeated Measure (without follow-up) | Substance abuse, |
| Peñas-Lledó et al., 2015 | Sweden | 615 | Community (Mixed Cohort, Girls) | To explore the cross-sectional and predictive effect of drive for thinness and/or negative affect scores on the development of self-reported anorexia nervosa (AN) and bulimia nervosa (BN) | Longitudinal (<5yr) | Drive for thinness, negative affect |
| Prefit, Candea, & Szentagotai-Tatar, 2019 | Worldwide | 96 studies (16795) | Mixed (Mixed cohorts, All genders) | To examine the associations between specific emotion regulation abilities (emotional awareness, emotional clarity) and strategies (acceptance of emotions, reappraisal, problem-solving, rumination, avoidance of emotions, and suppression), and eating pathology. | Systematic Review/ Meta-Analysis (combined) | Emotion regulation |
| Presnell et al., 2009 | USA | 496 | Community (Adolescents, Girls) | To clarify the temporal relations between depressive and bulimic symptoms using eight-year prospective longitudinal data from a community sample of female adolescents. | Longitudinal (>5yr) | Depression |
| Puccio et al., 2017 | Australia | 189 | Community (Adolescents, All Genders) | To explore the potentially longitudinal bi-directional effects of disordered eating symptoms with depression and anxiety | Longitudinal (<5yr) | Depression, anxiety |
| Reas, Pedersen, & Ro, 2016 | Norway | 672 | Inpatient (Young People, Women) | To investigate impulsivity-related personality traits using the Revised NEO Personality Inventory (NEO PI-R) in women diagnosed with cooccurring bulimia nervosa and borderline personality disorder (BN-BPD), borderline personality disorder (BPD no-BN), or major depressive disorder (MDD-only). | Cross-Sectional | Impulsiveness |
| Reyes-Rodriqguez et al., 2011 | USA, Canada, England, Germany | 753 | Outpatient (Young People, All Genders) | To describe the nature of traumatic events experienced and to explore the relationship between PTSD and anorexia nervosa (AN) in a sample of women | Cross-Sectional | PTSD |
| Robinson et al., 2020 | Europe | 1623 | Community (Adolescents, All Genders) | To characterise any association among disordered eating behaviours and other mental health disorders and to identify early associations with the development of symptoms over time. | Longitudinal (>5yr) | BMI, neuroticism, impulse control, addition-related behaviours, ADHD |
| Rojo-Moreno et al., 2015 | Spain | 993 | Community (Adolescents, All Genders) | To analyse both cross-sectional comorbidity and prospective longitudinal comorbidity (after two years) between eating disorders and other DSM-IV Axis I psychiatric disorders in a sample of adolescents from the community. | Longitudinal (<5yr) | Psychiatric disorders |
| Saraçlı et al., 2015 | Turkey | 423 | Outpatient (Adult, All Genders) | To investigate the prevalence and clinical correlations of night eating syndrome (NES) in a sample of psychiatric outpatients | Cross-Sectional | Body image, psychological distress, depressive disorder, impulse control disorder, nicotine dependency |
| Schaumberg  et al., 2020 | UK | 4864 | Community (Mixed Cohort, All Genders) | To examine whether social communication characteristics present in middle childhood (ages 8–14) were associated with eating disorder behaviours, cognitions, and diagnoses across adolescence (ages 14–18) in a large, population-based sample. | Longitudinal (>10yr) | Social communication characteristics (misattribution of faces as sad or angry, autistic-like social communication difficulties) |
| Sharpe et al., 2018 | USA | 1830 | Community (Young adults, All genders) | To examine the relative importance of dissatisfaction, overvaluation, and preoccupation with body weight and shape in predicting increases in disordered eating behaviours and depressive symptoms from adolescence through to early adulthood | Longitudinal (>10 years) | body image, depressive symptoms, disordered eating |
| Shivola et al., 2009 | Finland | 1318 | Community (Adolescents, All Genders) | To analyse the developmental relationships of adolescent-onset Axis I mental disorders and eating disorders | Repeated Measure (with follow-up) | Generalised anxiety disorder, major depressive disorder |
| Skinner et al., 2012 | USA | 4798 | Community (Mixed Cohort, All Genders) | To investigate the temporal relationship between depressive symptoms and overeating and binge eating among adolescent and young adult females in the United States | Longitudinal (<5yr) | Depression |
| Speranza et al., 2011 | France | 102 | Outpatient (Young People, All Genders) | To explore the relationships between alexithymia features and treatment options provided by professionals in a naturalistic prospective study of eating disorders | Longitudinal (<5yr) | Alexithymia |
| Stice et al., 2017 | USA | 1272 | Community (Mixed Cohort, Girls) | To examine risk factors that predict future onset each type of eating disorder and core symptom dimensions that crosscut disorders | Cross-Sectional | Negative affect, functional impairment, thin-ideal internalisation, body dissatisfaction, dieting, overeating, mental health care, positive thinness expectations, denial of cost of pursuing the thin ideal, and fasting, low BMI |
| Stice & Van Ryzin, 2019 | USA | 496 | Community (Adolescents, Girls) | To test the temporal sequencing of the emergence of risk factors hypothesised in a multivariate etiologic model of eating disorder development. | Repeated Measure (without follow-up) | Pressure to be thin, thin-ideal internalisation, body dissatisfaction, dieting, negative affect |
| Swinbourne et al., 2012 | Australia | 152 | Outpatient (Adult, Women) | To investigate the prevalence of comorbid eating and anxiety disorders in women presenting for inpatient and outpatient treatment of an eating disorder and women presenting for outpatient treatment of an anxiety disorder | Cross-Sectional | Anxiety disorders |
| Thiebaut et al., 2018 | Worldwide | 79 studies | Outpatient (Mixed cohorts, All genders) | To assess the association between sub-types of bipolar disorder (BD) (types I and II) and sub-types of eating disorders (EDs) (Anorexia Nervosa, Bulimia Nervosa, Binge-eating disorders) as well as their relative order of occurrence | Review (Systematic) | Bipolar disorder |
| Thiebaut et al., 2019 | France | 261 | Outpatient (Young People, All Genders) | To investigate the association between a dual diagnosis and severity in terms of clinical, neuropsychological dimensions and daily functioning | Cross-Sectional | Bipolar disorder |
| Thornton et al., 2011 | Sweden | 6086 | Community (Adult, Women) | (1) To document comorbidity patterns of AN and generalised anxiety disorder (GAD) in a large population-based sample of Swedish twins; (2) to explore the relation among AN, GAD, and BMI; and (3) to assess whether fasting and excessive exercise are more commonly endorsed by individuals with AN and GAD than those with AN or GAD only or the referent group of individuals with neither AN nor GAD. | Cross-Sectional | Generalised anxiety disorder |
| Thornton et al., 2016 | Sweden | 6899 | Community (Adult, Women) | To clarify the role of genetic and environmental factors in anorexia nervosa, major depressive disorder, and suicide attempts. | Review (Systematic) | Shared genetic factors |
| Utschig et al., 2010 | USA | 210 | Community (Adult, Women) | To investigate the role of fear of negative evaluation (FNE) in the context of the dual pathway model and its relationships with previously established risk factors | Cross-Sectional | Fear of negative evaluation, body dissatisfaction, negative affect, dieting |
| Vansteelandt, Probst, & Pieters, 2013 | Belgium | 57 | Inpatient (Young People, All Genders) | To examine differences in affective variability in eating disorders using an ecological momentary assessment (EMA) protocol | Repeated Measure (without follow-up) | Affect, affect variability |
| von Lojewski, Fisher, & Abraham, 2013 | Australia | 132 | Inpatient (Adult, All Genders) | To use the IPDE to study the DSM-IV PDs among the different ED diagnostic groups: (1) restrictive AN (AN-R), binge-purging AN (AN-BP), BN and ED not otherwise specified (EDNOS) and (2) for the different ED behaviours: self-induced vomiting, laxative use, objective binge eating and excessive exercising | Cross-Sectional | Personality disorder |
| Waxman, 2009 | Worldwide | 12 studies (1553) | Mixed (Mixed cohorts, All genders) | To conduct a systematic review of the current literature that examines impulsivity in individuals with eating disorders (ED) | Review (Systematic) | Impulsivity |
| Wolz, Granero, & Fenandez-Aranda, 2017 | Spain | 315 | Outpatient (Adult, All Genders) | To test a comprehensive model under control of eating disorder severity, in order to find independent predictors of food addiction | Cross-Sectional | Emotion regulation, negative urgency, self-directedness |
| Zhang et al., 2020 | Europe | 1386 | Community (Adolescents, All Genders) | To investigate early psychopathological and neuroanatomical risk factors for the development of disordered eating behaviours and comorbid mental health problems. | Longitudinal (<5yr) | Depression, ADHD, conduct disorder, grey matter volumes |
| **Socio-economic status** | | | | | | |
| Ahrén et al., 2013 | Sweden | 249,884 | Community (Mixed Cohort, All Genders) | To examine how parental characteristics and other aspects of family background were associated with the development of eating disorders (ED) in males and females | Longitudinal (>10yr) | Family socioeconomic status, parental age, family composition |
| Barry, Sonneville, & Leung, 2021 | USA | 851 | Community (Young Adult, All Genders) | To explore the relation between food insecurity and screening positive for an eating disorder among students attending a large, public Midwestern university. | Cross-Sectional | Food insecurity |
| Bould et al., 2016 | Sweden | 55059 | Community (Mixed Cohort, All Genders) | To investigate the rates of ED in female students between schools, and its association with parents with post-high school education after accounting for individual characteristics | Longitudinal (>5yr) | Proportion of female students, parent education |
| Goodman, Hashmati, & Koupil, 2014 | Sweden | 2138353 | Community (Mixed Cohort, All Genders) | To investigate which facets of parent and grandparent socio-economic position (SEP) are associated with eating disorders (ED), and how this varies by ED subtype and over time | Longitudinal (>10yr) | Parent and grandparent educational level, parent social class and income |
| Lydecker & Grilo, 2019 | USA | 873 | Community (Adult, All Genders) | To examine the relationship between food insecurity and bulimia nervosa | Cross-Sectional | Food insecurity |
| Mulders-Jones et al., 2017 | Australia | 6041 | Community (Adult, All Genders) | To ascertain the socio-geographic and socioeconomic distribution of disordered eating, using a population-based sample of adults in South Australia | Cross-Sectional | Socioeconomic status |
| West et al., 2019 | USA | 2179 | Community (Mixed Cohort, All Genders) | To examine the prevalence of several risk factors for binge eating by socioeconomic status (SES) and SES as a potential moderator of these risk factors. | Longitudinal (>5yr) | Socioeconomic status, overweight/obesity, body dissatisfaction, family weight teasing, dieting, food insecurity |
| **Body image, social influences, and gender** | | | | | | |
| Akgül et al., 2016 | Turkey | 60 | Inpatient (Young People, Men) | To describe not only medical and psychiatric characteristics, but also cultural features of adolescent males with an ED. | Longitudinal (>5yr) | Family structure, stressful life events, psychiatric disorder, family psychopathology history |
| Arcelus, Witcomb, & Mitchell, 2014 | Worldwide | 33 studies | Community (Young adults, All genders) | To systematically compile and analyse the rates of eating disorders in dancers. | Systematic Review/ Meta-Analysis (combined) | Dancing |
| Arduini, Lorio & Patacchini, 2019 | USA | 3535 | Community (Adolescents, All Genders) | To investigate whether the development of eating disorders, in the form of purging, is influenced by peers' body size through interpersonal comparisons. | Longitudinal (<5yr) | BMI, body comparisons |
| Bachner-Melman et al., 2009 | Israel | 574 | Community (Young adults, Women) | To compare the protective and acquisitive self-presentation styles of a group of women with anorexia nervosa (AN) with that of a group of female controls, and (2) to examine the hypothesis that protective self-monitoring would be positively associated with the degree of disordered eating in women, and that this association would be mediated by sociocultural attitudes towards appearance | Cross-Sectional | Protective self-presentation style, sociocultural attitudes towards appearance |
| Blashill, 2011 | Worldwide | 24 studies | Community (Young adults, All genders) | To review relationships between gender roles and (a) eating pathology, (b) body dissatisfaction, and (c) muscle dissatisfaction among men via meta-analysis. | Meta-Analysis | Gender roles |
| Bristow et al., 2020 | Worldwide | 12 studies | N/A | To review the existing literature on public health messages and eating-disorder-specific related outcomes | Review (Systematic) | Anti-obesity public health messages |
| Brook et al., 2019 | USA | 260 | Community (Mixed Cohort, All Genders) | To assess the prevalence of factors associated with Low energy availability (LEA), menstrual dysfunction (if female), and low BMD in an elite para athlete population, and to describe the differences among sex, disability type, and sport type in health issues stemming from LEA | Cross-Sectional | Para athletes |
| Calzo et al., 2017 | Worldwide | N/A | N/A | To summarize findings regarding (1) theory and mechanisms underlying sexual orientation disparities, (2) epidemiologic surveillance, and (3) treatment and prevention among diverse sexual orientation subgroups | Review (Narrative) | Sexual minorities |
| Dakanalis et al., 2015 | Italy | 405 | Community (Adult, Men) | To examine whether Poor Impulse Control (PIC), social anxiety (SA) and internalization of media ideals moderated the BD-ED symptomatology relationship among college men through latent moderated structural (LMS) equation modelling | Cross-Sectional | Impulse control, social anxiety, internalisation of media ideals |
| Dakanalis et al., 2017 | Italy | 2713 | Community (Adult, Women) | To examine the influence and utility of self-objectification and another four traditionally accepted vulnerability factors (i.e. body dissatisfaction, appearance-ideal internalization, dieting and negative affectivity) in predicting both the onset and maintenance of women’s (DSM-5) EDs at 4-year follow-up | Longitudinal (<5yr) | Appearance-ideal internalisation, body dissatisfaction, self-objectification, dieting, negative affectivity |
| Davison, Marshall-Fabien, & Gondara, 2014 | Canada | 5116 | Community (Mixed Cohort, All Genders) | To examine sex differences and eating disorder risk among psychiatric conditions, compulsive behaviours (i.e., gambling, suicide thoughts and attempts) and substance use in a nationally representative sample | Cross-Sectional | Sex differences |
| Day et al., 2011 | UK | 79 | Outpatient (Young People, All Genders) | To compare the clinical features and antecedent factors in a group of patients with an early onset of BN and EDNOS-BN with those developing their disorder at a more typical age | Cross-Sectional | Menarche age |
| Espinoza, Penelo, & Raich, 2010 | Spain | 128 | Community (Adolescents, All Genders) | To assess sociocultural and individual risk factors for eating and body image disturbances in Spanish adolescents | Repeated Measure (without follow-up) | [Eating attitudes, influence of the body shape model, extreme weight-control behaviours, body image and BMI](https://www.sciencedirect.com/topics/medicine-and-dentistry/body-mass-index) |
| Evans et al., 2017 | England | 516 | Community (Children, All Genders) | To identify within-time associations of eating disorder symptoms with measures of body dissatisfaction, depressive symptoms, and BMI all at 12 years of age, and to identify prospective predictors of eating disorder symptoms at 12 years of age by gender, taking into account prior eating disorder symptoms at 9 years of age | Longitudinal (>5yr) | Dietary restraint, depression, body dissatisfaction, BMI |
| Favaro et al., 2009 | Italy | 2459 | Outpatient (Adult, All Genders) | To explore the time trends in age at onset of anorexia nervosa and bulimia nervosa | Longitudinal (>10yr) | Year of birth |
| Forrest et al., 2018 | USA | 1084 | Community (Mixed Cohort, Girls) | To estimate ED symptom networks among people with anorexia nervosa (AN) and bulimia nervosa (BN) and among a combined group of people with AN and BN | Modelling (Statistical) | Shape and weight overevaluation, fearing weight gain, desiring weight loss, restraint, shape and weight preoccupation |
| Francisco, Narciso & Alarcao, 2013 | Portugal | 725 | Community (Adolescents, All Genders) | To compare female and male athletes, non-elite athletes and controls in terms of potential risk/protective factors for the development of eating disorders, levels of disordered eating, and the relationship between risk/protective factors and disordered eating. | Cross-Sectional | Athlete (elite vs. non-elite), social pressure, body dissatisfaction, parental influences, self-esteem |
| Gigi, Bachner-Melman, & Lev-Ari, 2016 | Israel | 262 | Community (Adult, Men) | To investigate whether the association between susceptibility to social messages and attitudes toward eating, weight and shape might explain, at least partially, the high levels of disordered eating consistently observed in gay and bisexual men, and whether gay and bisexual men are more susceptible than heterosexual men to social messages in general, or specifically to messages about physical appearance | Cross-Sectional | Body image, internalisation of attitudes toward appearance, influence of advertisements focused on physical appearance |
| Glashouwer et al., 2019 | Worldwide | 46 studies (4928) | Mixed (Mixed cohorts, All genders) | To systematically review the existing empirical evidence concerning the role of the cognitive-affective, perceptual, and behavioural components of body image disturbance in AN | Review (Systematic) | Body dissatisfaction |
| Goldschmidt et al., 2016 | USA | 1827 | Community (Young adults, All genders) | To characterize the longitudinal stability of four different eating-related categories (no overeating, overeating, binge eating, and binge eating disorder [recurrent binge eating with associated distress]) within a population-based sample followed over 10 years during the transition from adolescence to young adulthood | Longitudinal (>10 years) | Anthropometrics, eating behaviours, psychosocial factors |
| Gonzales & Blashill, 2021 | USA | 962 | Community (Adult, All Genders) | To assess the occurrence of probable EDs, ED symptoms, probable BDD, BDD symptoms, drive for muscularity, and APED misuse in an ethnically/racially diverse sample of sexual minorities in the U.S | Cross-Sectional | Race, gender, sexual minority |
| Gorrell et al., 2021 | USA | 13658 | Outpatient (Young People, All Genders) | To investigate patterns of ED symptomology among youth and adults (N = 13658) who telephoned treatment centres in the United States when seeking clinical support for ED symptoms | Cross-Sectional | Race, gender |
| Griffiths et al., 2018 | Australia, USA, UK, Canada | 228 | Outpatient (Adult, All Genders) | To evaluate a model explaining how exposure to thinspiration and fitspiration relates to eating disorder symptom severity among individuals with eating disorders | Cross-Sectional | Exposure to thinspiration and fitspiration media, physical appearance comparisons |
| Gulliver et al., 2015 | Australia | 224 | Community (Young adults, All genders) | To investigate Australian elite athletes’ symptoms of general psychological distress and common mental disorders | Cross-Sectional | Athlete |
| Hausenblas et al., 2013 | Worldwide | 33 studies | Community (Young adults, All genders) | To provide a statistical summary of the laboratory research examining the acute exposure to the media's portrayal of the ideal physique on eating disorder symptoms, along with body-image concerns, negative affect, and self-esteem, (2) to examine moderator effects, such as participant, media, delivery, and design features that might moderate intervention effects, and (3) to discuss theoretical, methodological, and statistical limitations of the literature and explore promising directions for future research in light of the findings | Systematic Review/ Meta-Analysis (combined) | Media exposure to thin ideal |
| Hazzard et al., 2019 | USA | 12040 | Community (Adult, All Genders) | To examine longitudinal associations between binge eating-related concerns (i.e., cognitions associated with binge eating, such as embarrassment over amount eaten and fear of losing control overeating) and depressive symptoms among U.S. young adults and assess whether associations differ by race/ethnicity | Longitudinal (>5yr) | BMI, depression, race |
| Hinojo-Lucena et al., 2019 | Worldwide | 12 studies for systematic review; 10 studies for meta-analysis | Community (Young adults, All genders) | To determine problematic internet use (PIU)-related eating disorders in students from the systematic review of the literature and (ii) to analyse the incidence of PIU in eating disorders through meta-analysis of the literature. | Systematic Review/ Meta-Analysis (combined) | Problematic internet use |
| Jett, LaPorte, & Wanchisn, 2010 | USA | 90 | Community (Adult, Women) | To assess whether exposure to pro-eating disorder (pro-ED) websites influences college women’s eating behaviours |  | Pro-ED website exposure |
| Klump et al., 2018 | USA | 964 | Community (Mixed Cohort, Girls) | To examine the effects of estrogen on genetic influences on binge eating during puberty in preadolescent and adolescent female twins | Cross-Sectional | Oestradiol levels |
| Klump, 2013 | Worldwide | N/A | N/A | To review data from human and animal studies in support of puberty as a critical risk period for eating disorders and evaluate the evidence for hormonal contributions | Review (Narrative) | Puberty |
| Kostrzewa, Eijkemans, & Kas, 2013 | UK | 778 | Community (Adult, Women) | To examine the relation between the expression of excessive exercise and the risk of obtaining an ED diagnosis in a sample of women representing the general population | Cross-Sectional | Excessive exercise |
| Kwan & Gordon, 2018 | USA | 187 | Community (Adult, All Genders) | To examine the role of acculturative stress and perceived discrimination on eating disorder symptoms among ethnic minority groups | Cross-Sectional | Acculturative stress, perceived discrimination |
| Mabe, Forney & Keel, 2014 | USA | Study 1: 960, Study 2: 84 | College (Adult, Women) | To assess whether Facebook use causes temporal changes in eating disorder risk factors, specifically weight/shape concerns and anxiety. | Cross-Sectional | ED risk factors |
| Mantilla, Bergensten, & Birgegard, 2014 | Sweden | 1337 | Outpatient (Young People, All Genders) | To identify associations between ED symptoms and distinct aspects of self-image in adolescents, and to compare these associations in a clinical sample versus a non-clinical sample, and in boys versus girls | Cross-Sectional | Self-image, gender differences |
| Martinsen & Sungodt-Borgen, 2013 | Norway | 966 | Community (Adolescents, All Genders) | To examine the prevalence of eating disorders among female and male adolescent elite athletes and nonathletic controls. | Cross-Sectional | Athletic status, weight-sensitive sport groups, athlete gender |
| McNicholas et al., 2012 | Ireland | 3031 | Community (Adolescents, All Genders) | To investigate associations between pubertal status and disordered eating and pubertal timing and disordered eating in male and female Irish adolescents. | Cross-Sectional | Puberty, disordered eating |
| Neumark-Sztainer et al., 2009 | USA | 412 | Community (Adolescents, All Genders) | To identify predictors of prevalence and incidence of disordered eating (binge eating and extreme weight control behaviours) among overweight adolescents. | Longitudinal (>5yr) | Exposure to weight loss magazines, weight importance, poor eating patterns |
| Neumark-Sztainer et al., 2011 | USA | 1257 | Community (Young Adult, All Genders) | To examine associations between participating in mind-body activities (yoga/Pilates) and body dissatisfaction and disordered eating (unhealthy and extreme weight control practices and binge eating) in a population-based sample of young adults | Cross-Sectional | Yoga/Pilates |
| O'Brien et al., 2017 | USA | 47,759 | Community (Adult, Women) | To assess a range of potential causes and consequences of eating disorders in hopes of informing public health practice and future research endeavours | Repeated Measure (with follow-up) | Race, parent education, recent birth, ED sister, smoking, underweight, depression, later first birth, bleeding and nausea during pregnancy, miscarriage |
| Phillipou, Castle, & Rossell, 2019 | Worldwide | 15 studies | Mixed (Adults, All genders) | To review those studies that have directly compared groups of individuals with anorexia nervosa (AN) and body dysmorphic disorders (BDD), to determine similarities and differences in presentation between the two conditions. | Review (Systematic) | Body dissatisfaction |
| Prnjak et al., 2021 | Australia | 1327 | Community (Adolescents, All Genders) | To investigate unique associations between aspects of body image and ED onset, distress, and quality of life in a community sample of adolescents prospectively after one year. | Longitudinal (<5yr) | Body image |
| Rodgers & Melioli, 2016 | Worldwide | 67 studies | Mixed (Mixed cohorts, All genders) | To review the literature examining the relationship between the use of Internet and social media and body image and eating concerns | Review (Narrative) | Disordered eating, body dissatisfaction/image, social comparison, internet addition, drive for thinness, fat stigmatisation |
| Rodgers et al., 2017 | USA | 2271 | Community (Adolescents, All Genders) | To explore interactions between ethnicity/race and overweight status on disordered eating behaviours in a population-based adolescent sample. | Cross-Sectional | Overweight, Minority status |
| Sepulveda, Carrobles, & Gandarillas, 2010 | Spain | 2551 | Community (Adult, All Genders) | To examine bio-socio-demographic and psychopathological variables by gender comparing high and low EDI scorers, to identify socio-demographic, psychopathological, family and lifestyle characteristics associated with unhealthy eating patterns in a college population by gender, and to explore a multifactorial model by gender including factors associated with a population that presents unhealthy eating patterns | Cross-Sectional | Gender, dieting, body dissatisfaction, levels of psychopathology, self-esteem |
| Shagar et al., 2019 | Australia, Malaysia | 820 | Community (Adult, Women) | To test a subsection of the Tripartite Influence Model, investigate whether sociocultural influences (family, peers, media) lead to thin ideal internalization, which in turn lead to body dissatisfaction (BD) and subsequently restrained eating and bulimic behaviours in both Australian and Malaysian female emerging adults | Cross-Sectional | Sociocultural influences, thin ideal internationalisation, body dissatisfaction |
| Sidor et al., 2015 | Europe (Romania, Hungry, Germany, Ukraine, Serbia, Slovakia) | 706 | Community (Adolescents, All Genders) | To assess significant gender differences in the association of anorexia nervosa and bulimia nervosa symptoms with depression and generalised anxiety symptoms in a community sample of adolescents. | Cross-Sectional | Gender, depression, anxiety |
| Sungodt-Borgen & Torstviet, 2010 | Worldwide | N/A | Community (Young adults, All genders) | To present an overview of the following aspects of disordered eating and eating disorders: (1) the disordered eating continuum, its model and definitions, (2) the prevalence and trends in the prevalence over time, (3) risk factors, (4) health and performance consequences, (5) how to approach athletes with symptoms of disordered eating, and finally (6) preventive strategies, in high-intensity sport (HIS) athletes | Review (Narrative) | Dieting, Athlete |
| Tan et al., 2016 | Worldwide | N/A | Community (Young adults, All genders) | To report on a quantitative and qualitative study into disordered eating and eating disorders, in which a range of conceptual and ethical difficulties raised clear problems for research, diagnosis, and treatment. | Review (Narrative) | Athlete (gymnast) |
| Thompson, Petrie & Anderson, 2017 | USA | 325 | Community (Young Adult, Women) | To determine whether the prevalence of eating disorder classifications (i.e., clinical eating disorder, subclinical eating disorder, and asymptomatic) and pathogenic weight control behaviours (e.g., bingeing, vomiting) change over a five-month sport season | Repeated Measure (without follow-up) | Athlete |
| Uniacke et al., 2021 | USA | 287 | Community (Mixed Cohort, All Genders) | To examine how identity development and minority stress relate to the presence of disordered eating behaviours and cognitions in transgender and gender nonbinary individuals, and improvement at one-year follow-up | Longitudinal (<5yr) | Transgender congruence, receipt of gender-affirming care, minority stress |
| Wheatley et al., 2012 | Worldwide | N/A | Community (Adults, Women) | To challenge the prevailing concept of female athlete triad and its relevance to athletes and the general population | Review (Critical) | Female Athlete triad |
| Wooldridge, Mok, & Chiu, 2014 | Worldwide | 12 forums | Community (Adult, Men) | To conduct a qualitative content analysis of male participation in pro-ana forums in an effort to learn more about male participation in these forums. | N/A | Participation in pro-eating disorder websites |
